# Inference of Splicing Regulatory Activities by Sequence Neighborhood Analysis

**DOI:** 10.1371/journal.pgen.0020191

**Published:** 2006-11-24

**Authors:** Michael B Stadler, Noam Shomron, Gene W Yeo, Aniket Schneider, Xinshu Xiao, Christopher B Burge

**Affiliations:** 1 Department of Biology, Massachusetts Institute of Technology, Cambridge, Massachusetts, United States of America; 2 Crick-Jacobs Center for Computational and Theoretical Biology and Laboratory of Genetics, The Salk Institute, La Jolla, California, United States of America; RIKEN Genomic Sciences Center, Japan

## Abstract

Sequence-specific recognition of nucleic-acid motifs is critical to many cellular processes. We have developed a new and general method called Neighborhood Inference (NI) that predicts sequences with activity in regulating a biochemical process based on the local density of known sites in sequence space. Applied to the problem of RNA splicing regulation, NI was used to predict hundreds of new exonic splicing enhancer (ESE) and silencer (ESS) hexanucleotides from known human ESEs and ESSs. These predictions were supported by cross-validation analysis, by analysis of published splicing regulatory activity data, by sequence-conservation analysis, and by measurement of the splicing regulatory activity of 24 novel predicted ESEs, ESSs, and neutral sequences using an in vivo splicing reporter assay. These results demonstrate the ability of NI to accurately predict splicing regulatory activity and show that the scope of exonic splicing regulatory elements is substantially larger than previously anticipated. Analysis of orthologous exons in four mammals showed that the NI score of ESEs, a measure of function, is much more highly conserved above background than ESE primary sequence. This observation indicates a high degree of selection for ESE activity in mammalian exons, with surprisingly frequent interchangeability between ESE sequences.

## Synopsis

Gene expression involves a series of steps in which specific short DNA or RNA segments are recognized by nucleic acid–binding proteins. One step that is particularly prominent and complex in humans and other vertebrates is the removal of introns and the ligation of exons in the process of pre-mRNA splicing. To better understand the sequences in exons that regulate this process, the authors have developed a method termed Neighborhood Inference that predicts the splicing regulatory activity of RNA segments based on the known splicing enhancer or silencer activity of other segments that have closely neighboring sequences. This method is applied to predict hundreds of new exonic splicing regulatory elements, as well as splicing-neutral sequences. A number of these predictions were validated experimentally, indicating that the number of exonic splicing regulatory sequences is larger than previously suspected. Neighborhood Inference scoring is also used to show that selection on exonic splicing enhancers (ESEs) frequently allows conversion of one ESE sequence to another over evolutionary time periods, suggesting that ESEs are, to at least some degree, interchangeable in constitutively spliced exons. The methods described may also find application in the study of other biomolecular processes that involve sequence-specific nucleic acid–binding proteins.

## Introduction

The basic cellular processes of transcription, translation, and pre-mRNA splicing all rely extensively on sequence-specific recognition of short nucleic-acid segments to achieve specificity and regulation. Studies of the specificity of these processes typically yield sets of sequence elements that are bound by a given protein or complex, or that share a common activity. Protein-binding sites on DNA or RNA are typically modeled by some form of position-specific scoring matrix (PSSM) model [1], constructed from aligned sets of experimentally determined binding sequences. Hundreds of such models are collected in databases such as TRANSFAC and JASPAR [2,3]. However, PSSMs cannot be directly derived from heterogeneous sets of binding sites, such as those identified in screens based on activity [4–8]. Derivation of PSSMs from such data typically requires clustering and alignment steps, as performed by algorithms implemented in MEME [9], the Gibbs Motif Sampler [10], and PROCSE [11]. Even binding sites obtained for a single protein factor may be heterogeneous and only suboptimally modeled by a single PSSM, e.g., for a transcription factor whose binding is influenced by participation in different complexes [12]. Although PSSMs have proven useful in numerous applications, they are often used in cases where the underlying assumption of independence between positions remains untested, and this assumption has proven incorrect in some well-characterized protein–DNA interactions [13,14]. Using simulations it has been shown that hundreds or even a few thousand binding sites are necessary to accurately model a given binding site that violates independence between positions, while experimental approaches typically used in the past yield only ~20–70 sites [14]. While PSSMs and the method presented here focus on accurate modeling of binding sites on nucleic acids, whether or not any given site is functional may also frequently depend on sequence context.

**Figure 1:**
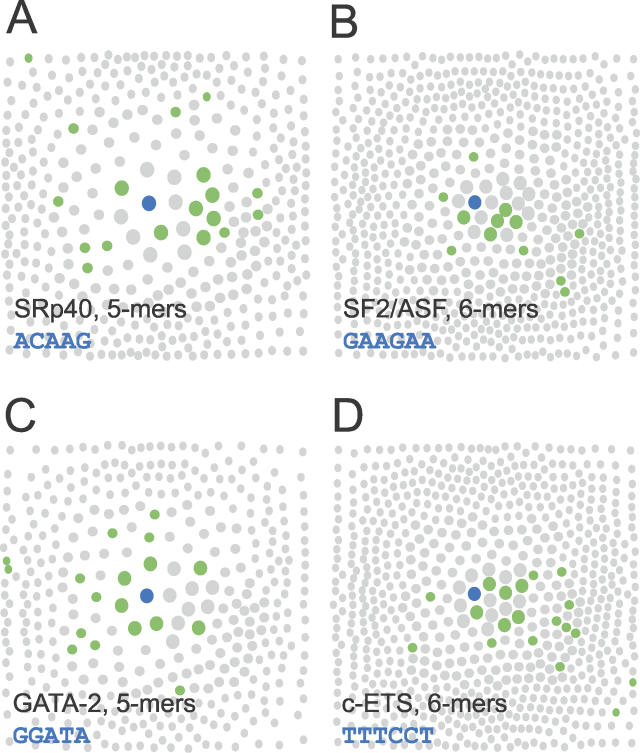
Visualization of Sequence Neighborhoods with Experimental Binding Site Sequences Circles represent known binding site sequences (green) or related sequences (gray) in the sequence neighborhood of the consensus binding site sequence (blue). Large, medium, and small circles correspond to sequences with one, two, and three mismatches, respectively, compared to the consensus sequence. (A) SRp40 binding pentamers [24] around the consensus ACAGG (total of 376 pentamers shown). (B) SF2/ASF binding hexamers [27] around GAAGAA (total of 694 hexamers shown). (C) GATA-2 binding pentamers [25] around GGATA (total of 376 pentamers shown). (D) c-ETS binding hexamers [26] around TTTCCT (total of 694 hexamers shown).

We have designed an algorithm called Neighborhood Inference (NI) that exploits the observation that the sites bound by DNA- and RNA-binding proteins tend to cluster tightly in sequence space (Figure 1). NI predicts the activity of sequences using the local density of known sites in sequence space, effectively extrapolating from a set of known elements that may not be comprehensive. In contrast to standard approaches, NI does not require that the set of known sites be aligned or even homogeneous (i.e., contain binding sites for only a single protein), as multiple potentially overlapping motifs can be modeled simultaneously, including both positively and negatively acting elements.

As an application, NI was used to model two classes of RNA elements involved in the regulation of pre-mRNA splicing: exonic splicing enhancers (ESEs) and exonic splicing silencers (ESSs). These elements act in concert with the classical 5′ and 3′ splice site motifs and the branch signal in the recognition of exons in metazoans, and play important roles in regulation of alternative splicing [15,16]. Most ESEs are recognized by members of the serine/arginine-rich (SR) protein family [17], which recruit the spliceosomal machinery to define exon locations and promote usage of nearby splice sites. In contrast, most known ESSs are thought to interact with members of the heterogeneous nuclear ribonucleoprotein protein family, which can act to repress recognition of adjacent splice sites [7,18–20]. ESEs are thought to be present in a great majority of all human exons [21,22], and ESSs also appear to be very widespread, especially in alternatively spliced exons [7,8,20]. Splicing regulatory sequences sometimes exert variable regulatory effects on splicing, depending on their relative locations in the exon [23].

Here we show that NI can predict the splicing activity of arbitrary RNA oligomers from heterogeneous sets of known regulatory sites, and that combined usage of functionally antagonistic ESE and ESS sets mutually improves their prediction. The validity of the predictions made by the NI approach is supported by multiple computational and experimental lines of evidence, leading to the conclusion that the number of exonic splicing regulators is substantially larger than previously anticipated. Because the assumption that similarly acting regulatory sites tend to cluster in sequence space is likely to hold very generally beyond splicing regulation, it will be interesting to see how well the NI approach performs when applied to *cis-*regulators of transcription, polyadenylation, and other processes.

## Results/Discussion

### Representation and Clustering of Binding Sites in Sequence Space

The motivation for our approach comes from considering the known binding sites for a sequence-specific DNA- or RNA-binding factor in the context of the set of all similar sequences of the same length, which we refer to as the sequence neighborhood. The pattern of relatedness among the binding sites for a factor can be visualized using a “local sequence neighborhood diagram” (Figure 1), in which distinct nucleic-acid sequences of a given length are represented as circles whose sizes and relative positions reflect the degree of similarity between the corresponding sequences. Known binding sites for representative nucleic acid–binding proteins that have well-characterized in vitro binding specificities—the splicing factors SRp40 and SF2/ASF and the transcription factors GATA-2 and c-ETS [24–27]—are shown in Figure 1. The degree of clustering observed for the known binding sites of each factor, in a region of sequence space containing only a small fraction of the total set of pentamers or hexamers, suggests the use of proximity in sequence space to known sites as a predictor of binding activity. Some aspects of this proximity effect could be captured by modeling the binding specificity of each of these factors by a PSSM. However, PSSM models are directly applicable only to sequence sets that are sufficiently homogeneous and can be reliably aligned.

### Inference of Activity from Sequence Neighborhood

**Figure 2:**
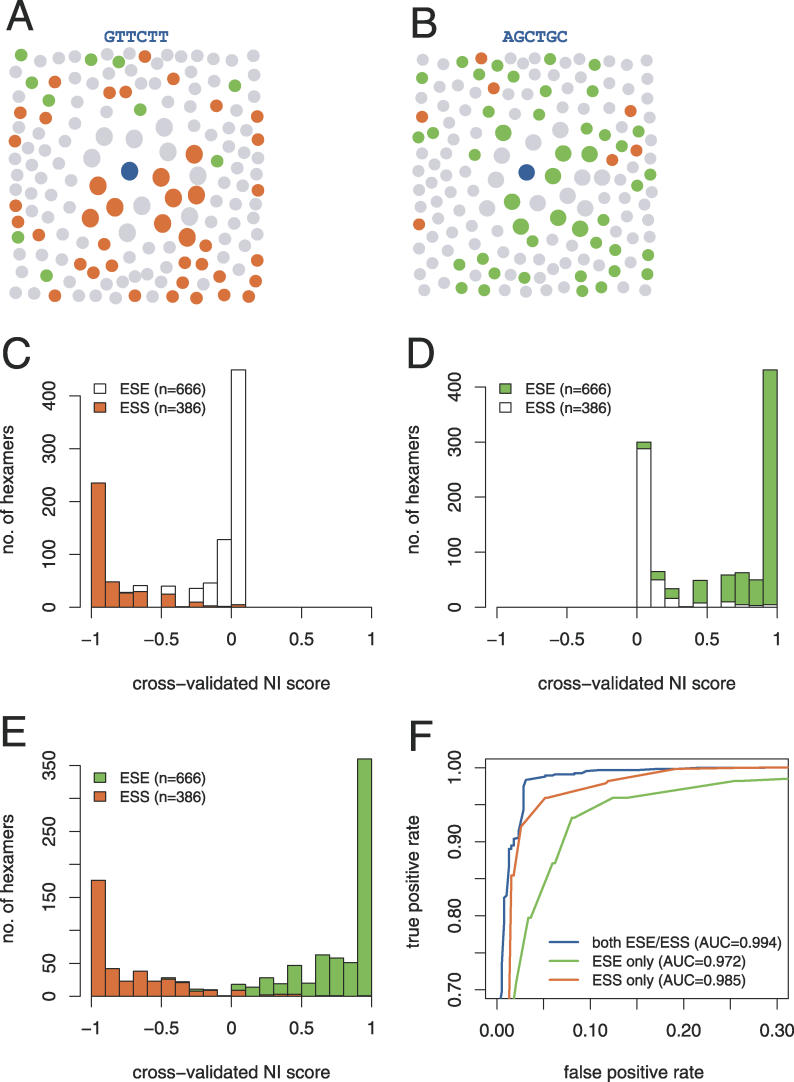
Cross-Validation Analysis of NI Scoring (A) Local sequence neighborhood diagram for sample predicted ESS sequence (GTTCTT, blue dot), showing trusted ESSs (orange) and ESEs (green) in neighborhood. (B) Similar diagram for predicted ESE sequence AGCTGC. (C–E) Histograms of 10-fold cross-validated NI scores, using only trusted ESS (C), only trusted ESE (D), or both ESS and ESE hexamers (E) as training data. (F) Comparison of NI performance in different cross-validation experiments. False-positive and true-positive rates defined in Materials and Methods.

In recent years, large-scale screens for ESE and ESS elements have been conducted, resulting in the identification of substantial sets of oligonucleotides that are predicted with fairly high confidence to have ESE or ESS activity. These sequence sets are heterogeneous, each likely containing binding sites for at least a dozen distinct splicing factors, and are not readily modeled by standard motif-modeling methods. To more accurately model the set of human exonic regulatory elements, and to identify additional elements missed by these screens, we used the NI approach, which estimates the potential activity of sequences based on the density of known sites contained in a local sequence neighborhood. In this approach, the set of sequences at different “distances” (measured in terms of substitutions and/or shifts) from a given query sequence is evaluated, and a score is assigned that reflects the nature of the known sequences in the neighborhood (positive if mostly ESEs, negative if mostly ESSs, and near zero otherwise). The density of known elements in the neighborhood is also considered, with higher densities giving rise to scores of higher magnitude, and the raw score is normalized to the range −1 to +1. For example, using the ESE and ESS datasets described below, the sequence GTTCTT was assigned the highly negative NI score of −0.999 because its local sequence neighborhood contains a very high density of known ESS sequences and few ESEs (Figure 2A). Conversely, the sequence AGCTGC was assigned the highly positive NI score of 0.997 because of its proximity to a high density of known ESEs and relatively few ESSs (Figure 2B). Importantly, this method does not require the sets of positive and negative sequences to be homogeneous, e.g., binding sites for a single factor, and does not make explicit assumptions about statistical independence between positions in sites.

In concept, NI is related to the smoothing technique used in the primary sequence ranking method described by Aalberts and coworkers for 5′ splice site prediction [28]. NI applies to a situation in which input sequences are known to have or not have a particular biological activity. The primary sequence ranking method applies to a different situation in which the same sequence is known to have activity in one context but not another. In the primary sequence ranking method, sequences are ranked based on the ratio of the number of times they appear in training sets of real and decoy sites, and these ratios are smoothed by adding pseudocounts based on the corresponding ratios for sequences that are one or two mutations away.

Application of the NI approach requires designation of a set of trusted “positive” (e.g., ESE) oligonucleotides, and can optionally make use of a second set of trusted “negative” (e.g., ESS) sequences. For this purpose, we combined the results of three recent large-scale screens for exonic splicing regulatory elements [7,8,22]. In the first study, the RESCUE-ESE method identified candidate ESEs as hexanucleotides that are significantly overrepresented in exons versus introns and in exons with weak splice sites relative to exons with strong splice sites, and ESE activity was confirmed for a representative set of predicted ESEs using a splicing reporter assay [22]. In the second study, candidate ESE and ESS octamers called PESEs and PESSs were identified based on the relative frequency of octanucleotides in internal noncoding exons versus unspliced pseudo exons and 5′ untranslated regions; again, a subset were confirmed to have the predicted activity using splicing reporters [8]. In the third study, a cell fluorescence-based screen called FAS-ESS was used to recover decanucleotides with ESS activity from a library of random decamers introduced stably into a splicing reporter gene in cultured cells [7]. These studies all appeared to have relatively low rates of false positives based on splicing reporter assays, but had unknown rates of false negatives. From each of these sets of oligonucleotides, overrepresented hexamers were derived. The sets of RESCUE-ESE and PESE hexamers were combined to produce a set of 666 trusted ESEs, and the FAS-ESS and PESS hexamers were combined to produce a set of 386 trusted ESSs. These datasets are summarized in Table 1.

**Table 1:**
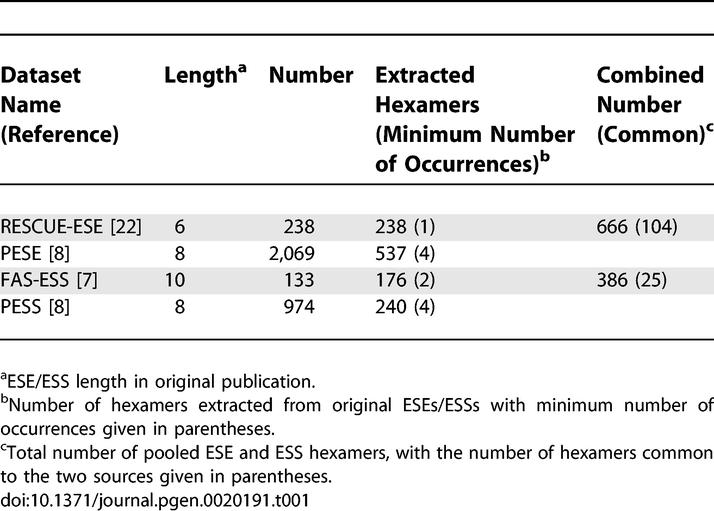
Trusted ESE/ESS Hexanucleotides

NI was applied systematically to predict exonic splicing regulatory elements using different trusted sets as input. Initially, the predictive power of NI scoring based on single sets of trusted sequences (either ESSs or ESEs) was compared to that achieved with both sets together, using cross-validation analysis. Because no datasets or systematic screens for sequences that are inactive in splicing have been reported to date, accuracy was assessed exclusively on the sets of known ESS and ESE hexamers, with the ESEs effectively functioning as negatives for assessment of ESS prediction, and vice versa. With the trusted ESS hexamers alone as input (resulting in a score range of −1 to zero), the NI score distributions for the known ESSs and ESEs were plotted (Figure 2C), using 10-fold cross-validation scoring, in which the known ESSs were broken into ten equal groups and each group was scored by NI using the remaining nine groups as trusted ESSs. These ESS-based scores gave very good separation between the known ESSs and ESEs, with a substantial majority of known ESSs being assigned scores below the lowest-scoring ESE hexamer (Figure 2C), suggesting that the NI approach is quite accurate in this “single-set” mode. Using the trusted ESEs as input (resulting in an NI score range of zero to one), 10-fold cross-validation scoring also resulted in fairly good discrimination of these two sequence sets (Figure 2D), although the separation was not as crisp as for the ESS-based scoring. The reasons for the improved NI classification using ESSs rather than ESEs as input are not entirely clear; perhaps the ESS set exhibits better clustering properties or is of higher quality than the ESE set.

Excellent separation of ESEs from ESSs was achieved when both ESSs and ESEs were used as input in 10-fold cross-validated NI scoring (Figure 2E), with almost all ESEs scoring above zero and almost all ESSs scoring below zero. These results suggest that the NI method has excellent potential for predicting splicing regulatory activity. Comparison of the classification performance of these three approaches using receiver–operator curve analysis (Figure 2F) confirmed that using both sets gave better discrimination than using the ESS set alone (area under curve [AUC] = 0.994 versus 0.985), which in turn was better than using the ESE set alone (AUC = 0.972), over essentially the entire range of false-positive levels. Similar results were obtained using 2-fold cross-validation (Figure S1). It is important to keep in mind that all of the accuracy measurements shown in Figure 2 assess the ability of the methods to discriminate ESSs from ESEs (and ESEs from ESSs), not their ability to discriminate “splicing-active” from “splicing-neutral” sequences. The latter classification problem is not readily assessed through cross-validation analysis because it would require a large dataset of high-confidence splicing-neutral sequences and such a dataset has not yet been determined. Instead, experimental tests were used to assess this level of classification, as described below.

Application of NI scoring to the entire set of 4,096 hexamers using the trusted ESS and ESE hexamers as input led to identification of hundreds of candidate novel ESS and ESE hexamers at score cutoffs for which the numbers of misclassifications of ESEs/ESSs in the cross-validation analyses were negligible (Table 2). For example, using NI score cutoffs of ≥0.80 for prediction of ESEs and ≤−0.80 for prediction of ESSs gave over 300 new candidate ESE hexamers and over 100 new candidate ESSs. The complete set of NI scores for all hexanucleotides is given in Table S1.

**Table 2:**
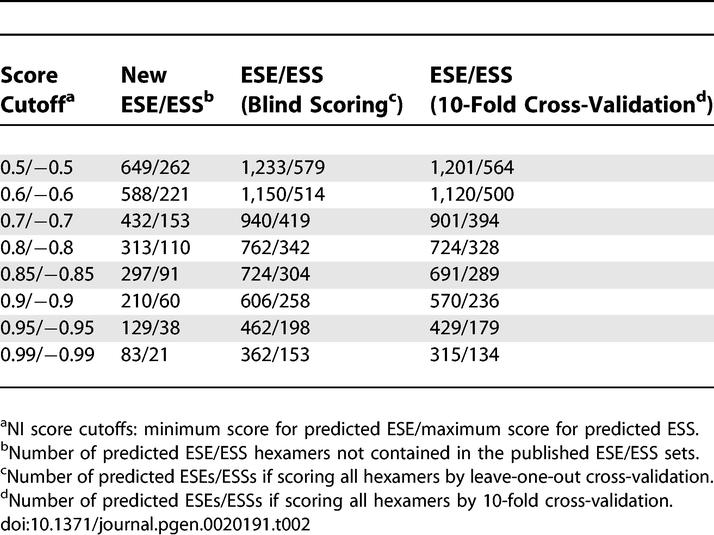
NI Prediction of ESE/ESS Hexamers

### Experimental Validation of NI Predictions

To further assess the accuracy of NI predictions, we asked whether NI scoring could predict the magnitudes of the effects on splicing of point mutations introduced into exons. For this analysis, pairs of tested wild-type and mutant exon sequences with corresponding exon-inclusion levels measured following transient transfection into cultured cells were extracted from three published studies of splicing regulatory sequences [7,8,22]. For each wild-type/mutant pair, the maximum change in NI score (over all corresponding pairs of altered hexamers) was compared to the experimentally determined change in exon-inclusion level, measured directly by quantitative RT-PCR in two of the studies or indirectly by the percentage of green fluorescent protein–expressing cells in a third (Figure S3). A highly significant correlation was observed between the change in NI score and the measured change in exon-inclusion level (*r* = 0.661, *p* = 1.9 × 10^−6^), providing support for the ability of NI scoring to predict changes in splicing regulatory activity resulting from point mutations.

**Figure 3:**
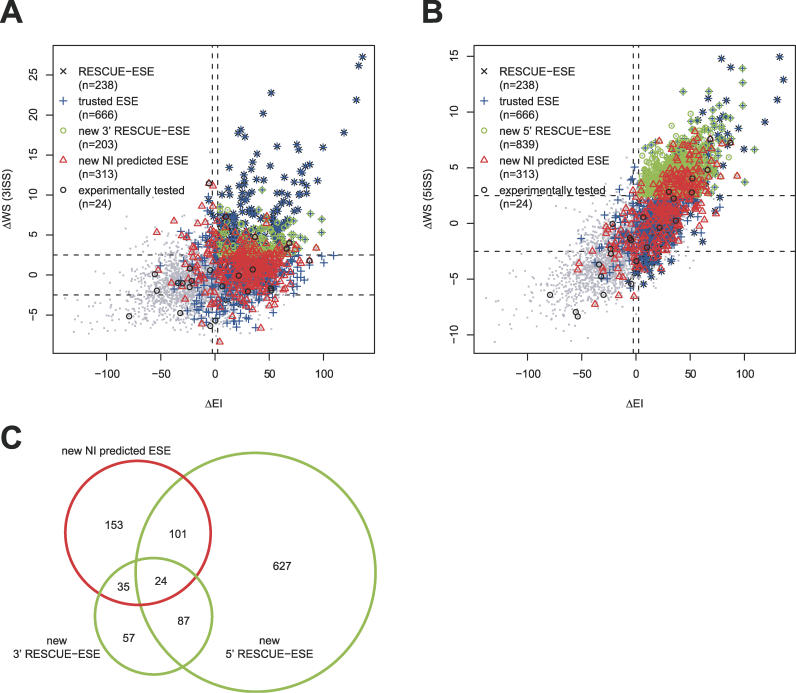
Comparison of NI- and RESCUE-ESE-Predicted ESEs (A and B) For each hexamer, the scatter plots show the enrichment in exons versus introns (ΔEI, *x*-axis), and the enrichment in exons with weak versus exons with strong splice sites (ΔWS, *y*-axis), as described by the original RESCUE-ESE method [22]. ΔWS values for 3′ splice sites are shown in (A), and for 5′ splice sites in (B). “RESCUE-ESE” were predicted to have ESE activity for at least one splice site [22], “trusted ESE” were used as NI training data, “new 3′/5′ RESCUE-ESE” fulfill the conditions ΔEI ≥ 2.5 and ΔWS ≥ 2.5, but were not in the original RESCUE-ESE, “new NI predicted ESE” have NI scores ≥ 0.8, and “experimentally tested” were selected for testing in a splicing reporter construct (Figure 4). Because over 4,000 points are plotted, in dense regions of the plot many symbols may not be clearly visible because of other overlapping symbols. Note also that the use of small gray dots for non-ESE hexamers in the figure may cause this group to appear less numerous than it actually is. Alternative versions of (A) and (B) with altered plotting order are provided in Figure S4. (C) Area-proportional Venn diagram with overlaps between sets with newly predicted ESEs from (A) and (B).

Previously, statistical enrichment of hexanucleotides in exons versus introns and in exons with weak splice sites relative to exons with strong splice sites has been used to predict ESEs with the RESCUE-ESE method [22]. For comparison, similar enrichment scores were determined for all hexamers including the new NI-predicted ESEs using updated sequence sets and splice site scoring methods (Figure 3).

**Figure 4:**
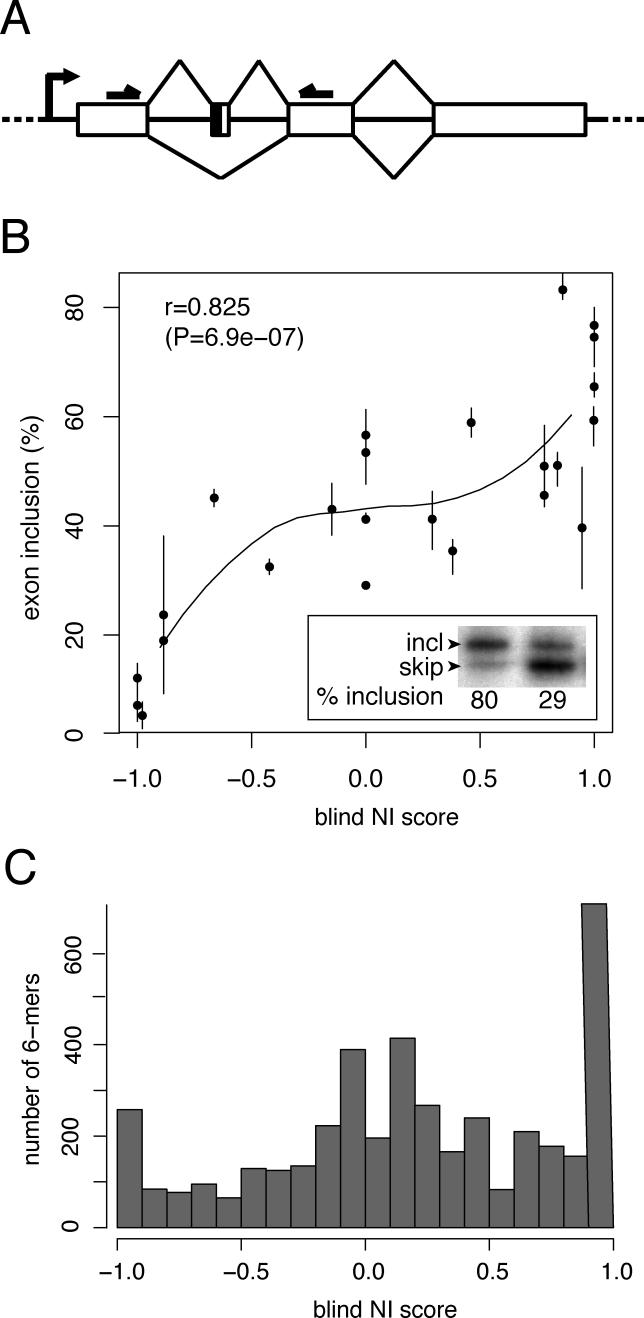
Prospective Tests of NI Predictions (A) Schematic representation of the splicing reporter construct and primers (horizontal arrows) used to quantify exon inclusion. Exons are represented as boxes. The vertical line in exon two indicates the cloning site for test sequences. (B) For each tested hexamer sequence, the NI score (*x*-axis) is plotted against the corresponding exon inclusion percentage (*y*-axis) determined by radioactive RT-PCR. All sequences were tested at least twice, and circles represent average values, with minimum and maximum values indicated by vertical lines. The curved line represents a local polynomial fit to the data. The insert shows a representative gel image for two arbitrarily chosen test sequences (AATCGC, 29% inclusion, and GACGAG, 80% inclusion). (C) Distribution of NI scores for all 4,096 DNA hexanucleotides using “blind” (leave-one-out) cross-validated scoring.

Old and new RESCUE-ESE hexamers overlapped substantially, with ~85% of original RESCUE-ESEs also predicted in the new analysis (Figure 3A and 3B). The updated RESCUE-ESE hexamers are more numerous than the set identified in 2002, presumably because of the increased statistical power resulting from improvements in the quality and quantity of exon and intron data available, and improved splice site models.

The new NI-predicted ESEs show a clear tendency to be enriched in exons versus introns, and to a lesser degree also in exons with weak splice sites versus exons with strong splice sites. However, a subset of NI-predicted ESEs are enriched in introns, in exons with strong splice sites, or both, as are some of the training hexamers (“trusted ESEs”) derived from the PESE method. About half of the NI-predicted ESEs were also contained in the updated RESCUE-ESE set. However, a substantial number of hexamers were predicted by only one of the two methods, raising the possibility of using both methods in conjunction to increase sensitivity and/or specificity. A total of 27 NI-predicted ESEs and five NI-predicted ESSs were also found in the set of exonic regulatory sequences identified by Goren and colleagues based on overrepresented and conserved dicodons in orthologous human and mouse exons [23].

To test the ability of NI scoring to identify novel ESEs and ESSs and to discriminate them from exonic splicing-neutral sequences, 24 previously uncharacterized hexanucleotides, chosen to cover the entire spectrum of NI scores from −1 to +1, were inserted into a splicing reporter construct (Figure 4A). The selected hexamers span a range of RESCUE-ESE enrichment values (black circles, Figure 3A and 3B). The splicing reporter construct was engineered to give close to 50% inclusion when inserted with random sequences, a level from which either enhancement or repression could be detected. The effects on splicing of the test exon were assayed by measuring the inclusion level of the test exon by radiolabeled RT-PCR following transfection of the reporter into cultured cells (Figure 4B). The level of exon inclusion varied dramatically as a function of the NI score of the inserted hexamer, giving very high positive correlation (*r* = 0.825, *p* = 6.9 × 10^−7^) between the NI scores of hexamers and the resulting level of inclusion of the test exon. The effects on splicing appeared to divide roughly into three score regions: below −0.8, between −0.8 and 0.8, and above 0.8. All tested sequences with NI scores in the middle region, between −0.8 and 0.8, had modest effects or no effects on splicing, resulting in roughly background levels of exon inclusion in the range of ~30%–60%, suggesting that sequences in this score interval are mostly splicing-neutral, at least in this exonic context, in the sense that they lack significant ESE or ESS activity. At the extremes, all five tested sequences with NI scores below −0.8 had significant ESS activity, reducing the average inclusion level to ~25% or below, and four of the seven tested sequences with NI scores above 0.8 had significant ESE activity, producing average inclusion levels above 60%. These predicted ESEs include two that were also predicted by the updated RESCUE-ESE analysis and two that were predicted uniquely by the NI method (AAACTG and AACTTA). These results confirm the ability of NI scoring to predict new ESS and ESE hexamers at the given cutoffs, and suggest that most or all hexanucleotides with NI scores below −0.8 are ESSs and that many or most of those with NI scores above 0.8 are ESEs. We note that here we have tested only one construct and one cell line. The activity of some of these elements may be context-dependent, a feature that is not modeled by NI. Some elements may only be active in certain tissues when bound by tissue-specifically expressed splicing factors, or at certain locations when bound by factors that need to be properly positioned relative to other components of the splicing machinery. Altogether, NI scoring appears able to partition hexamers into three subsets, containing predominantly ESSs, predominantly splicing-neutral sequences, and predominantly ESEs, as suggested by the trimodal distribution of NI scores for all hexamers (Figure 4C).

As expected, the densities of new NI-predicted ESEs and ESSs in exons and introns near splice sites followed the same pattern as for the trusted ESE and ESS sets, respectively, with ESEs occurring more frequently in exons than introns, and ESSs being more abundant in introns than exons. The shape of the density distribution was also somewhat similar between predicted and trusted ESEs and between predicted and trusted ESSs (Figure S2).

### Predicted ESE Hexamers Exhibit Sequence and Functional Conservation

**Figure 5:**
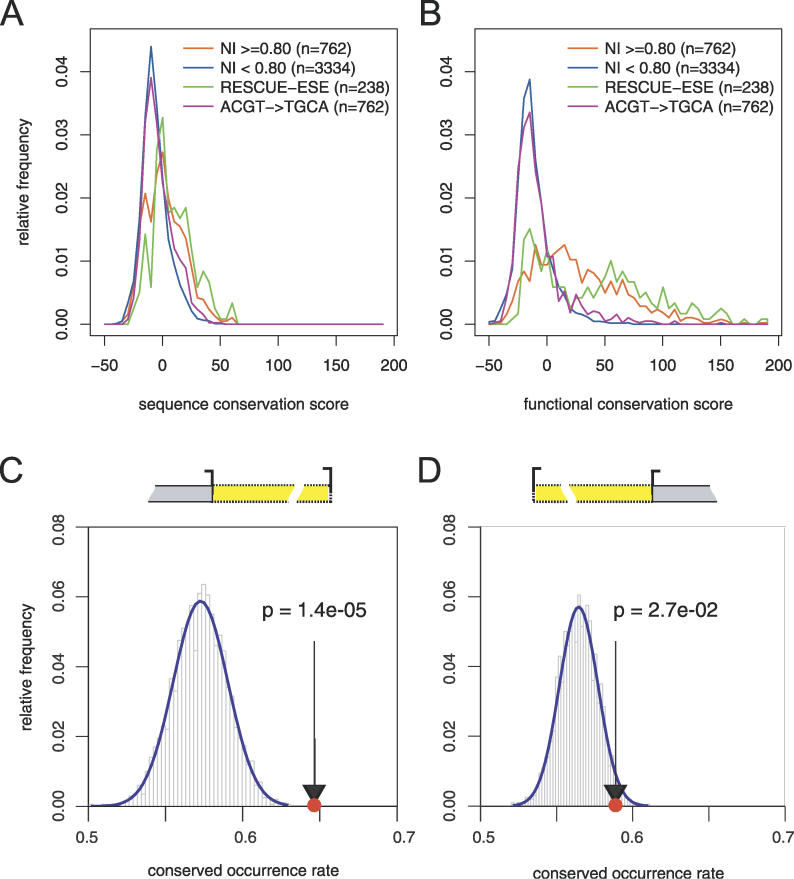
Conservation of Predicted ESE and ESS Hexamers (A and B) Phylogenetic conservation of NI-predicted ESEs was measured across human, mouse, rat, and dog genomes. Conservation of predicted ESE hexamers (orange curve, NI score ≥ 0.8) was compared to the conservation of all other hexamers (blue curve, NI score < 0.8), RESCUE-ESE hexamers (green line), or a control set of equal size (purple curve, randomized by nucleotide substitution as described in text) in a histogram of conservation scores, either measuring conservation of sequence (A) or conservation of function (B) as predicted by NI. (C and D) Conservation of NI-predicted ESSs was estimated using the COR measure as described in the Materials and Methods. The histograms represent COR values obtained for control sets of hexamers, and the red circle indicates the value for predicted ESS hexamers located between alternative 5′ splice sites (C) or between alternative 3′ splice sites (D). The analyzed exonic region between alternative splice sites is illustrated by a yellow box, with splice sites represented by brackets pointing to the left and right for 5′ and 3′ splice sites, respectively.

When the RESCUE-ESE method was applied to exon/intron datasets from mouse and other vertebrates, the resulting sets of candidate ESE sequences were quite similar to the set originally identified in human [29]. This observation, together with the high degree of conservation of domain organization among SR proteins across vertebrates [29], suggests that most or all hexamers with ESE activity in human are likely to have this activity in mouse as well. In general, most exons are expected to experience strong selection to preserve the core splice site and ESE sequences required for efficient and accurate splicing [30], and there is evidence from population genetic data of fairly strong selection to conserve RESCUE-ESE hexamers in constitutive human exons [31,32]. Thus, functional ESEs that occur in human exons are likely to be preferentially conserved in orthologous mammalian exons. The degree of conservation of candidate ESEs and other hexamers in orthologous exons of human, mouse, rat, and dog was assessed using a “sequence conservation rate” metric (Figure 5). As expected, the sequence conservation rates for the set of RESCUE-ESE hexamers were on average substantially higher than for hexamers overall. For the set of NI-predicted ESE hexamers, using a score cutoff of 0.8 and leave-one-out (blind) scoring, a shift toward higher conservation rates, almost as great as those seen for the RESCUE-ESE hexamers, was observed. Higher conservation of NI-predicted ESEs was observed whether comparing to all hexamers or to a control set of hexamers constructed by changing each adenine, cytosine, guanine, and thymine in the NI-predicted ESEs to thymine, guanine, cytosine, and adenine, respectively, yielding a set of identical size, sequence complexity, and cytosine/guanine content. The increased conservation supports the common in vivo activity of many NI-predicted ESEs in endogenous human exons.

The degree to which different ESE sequences can substitute for one another in evolution is not generally known. Previously, analysis of single nucleotide polymorphism data obtained evidence of fairly strong selection against “ESE-disrupting” changes, i.e., mutations that change RESCUE-ESE hexamers to non-RESCUE-ESE hexamers. Slightly weaker selection was observed against “ESE-altering” changes, i.e., mutations that change one (or more) RESCUE-ESE hexamer into another. To ask whether ESEs are functionally interchangeable over longer periods of evolutionary time, we studied ESE evolution in mammalian exons using a measure that we call functional conservation rate. Instead of requiring perfect conservation at the sequence level, this measure tallies the fraction of aligned hexamers in orthologous human/mouse/rat/dog exons at which the NI scores of the hexamers present in the other mammals differ by no more than a small amount from the NI score of the human hexamer (Figure 5B). The functional conservation rates for the RESCUE-ESE and NI-predicted ESE sets were found to be much higher than the rates of sequence conservation measured above (Figure 5A), and deviated to a much greater degree from the level of functional conservation measured for the control sets: for sequence conservation, the difference in means between predicted ESEs and control hexamers is less than 0.4 standard deviations, while for functional conservation, it is about 1.3 standard deviations. This result indicates that, over the longer periods of evolutionary time separating the divergence of rodents, canids, and primates, one ESE hexamer has frequently been substituted for another, suggesting a much greater degree of functional interchangeability between ESE hexamers than was previously appreciated. The high level of functional conservation suggests that ESEs tend to remain at particular positions in mammalian exons over long periods of evolutionary time, even as the precise ESE sequences present change.

### Predicted ESSs Are Conserved in Alternative Splice Site Exons

Unlike ESEs, which are highly conserved in constitutive exons (and exons overall), ESSs are not expected to be conserved in constitutive exons, which are presumably under selection primarily for efficient and accurate inclusion in the processed mRNA. Consistent with this expectation, hexamers of the FAS-ESS set have lower than average conservation rates in bulk exons (data not shown). Recently, Wang and coworkers showed that ESS oligonucleotides commonly influence splice site choice when placed between competing 5′ splice sites or between competing 3′ splice sites [20], and also regulate splice site usage in natural alternative 5′ splice site exons (A5Es) and alternative 3′ splice site exons (A3Es). They also observed that hexanucleotides of the FAS-hex3 set are preferentially conserved when located between alternative splice sites of orthologous human/mouse A5Es and A3Es, using a non-alignment-based conservation measure called conserved occurrence rate (COR). Applying this metric to the same sets of orthologous A5E and A3E pairs, we found that NI-predicted ESSs exhibit elevated rates of conservation relative to control sets of hexamers when located between alternative 5′ splice sites (Figure 5C; *p* = 1.4 × 10^−5^) or between alternative 3′ splice sites (Figure 5D; *p* = 2.7 × 10^−2^). This observation provides additional support for the common in vivo activity of NI-predicted ESS hexamers and suggests that, like other ESSs, they may commonly play a role in regulation of splice site choice.

### Perspectives

The large numbers of new ESEs and ESSs predicted at NI score cutoffs of 0.8 and −0.8, respectively, indicate that the number of exonic splicing regulatory elements is substantially larger than previously anticipated. The activity of many of these elements may be context-dependent. Some elements may only be active in certain tissues when bound by tissue-specific splicing factors, or at certain locations within a gene when bound by factors that need to be properly positioned relative to other components of the splicing machinery. Yet, the existence of such a large number of hexanucleotides with splicing regulatory activities has consequences for studies of related phenomena such as nonsense-mediated mRNA decay, implying that it may be challenging to design mutations in such a way that they influence only the process under study without having potentially confounding effects on pre-mRNA splicing. Already, this has proven to be an important issue, with a number of mutations that introduce premature termination codons in transcripts found to also have effects on splicing, often contributing to exon skipping by disrupting ESEs and/or creating ESSs [33,34]. NI scoring may prove useful in the design and interpretation of such experiments: not only can it be used to identify comprehensive sets of candidate ESE and ESS sequences, but it can also reliably predict splicing-neutral sequences (Figure 4). The identification of splicing-neutral sequences in human exons might also have utility in genetic engineering and/or synthetic biology applications when it is desired to introduce sequence changes to an exon without altering existing splicing regulation. NI scoring might also contribute to integrated models of pre-mRNA splicing, such as the ExonScan splicing simulation algorithm [7].

The NI approach itself may also have other applications. For modeling of regulatory sequence elements, NI may have advantages over traditional methods such as PSSMs, which have been used to predict ESEs [35], including its applicability to heterogeneous training data, thus eliminating the need for clustering or even alignment of input sequences. The NI approach does not make prior assumptions about the nature of the set of functionally active sequences except that local sequence neighborhoods are dense in functionally similar sites. Of course, the NI approach requires a relatively large set of training data to make accurate predictions. When only a small sample of binding sites are known (or in the rarer case where a larger sample of sites is known and the independence assumptions are shown to hold), PSSMs are probably a better choice.

PSSMs are based on simple physical principles that lead to a model of additive position- and residue-specific energies. The NI approach is based on two principles that have both evolutionary and physical aspects. The first is that nucleic acid–binding proteins are evolutionarily designed to bind to a set of oligonucleotides that are close to each other in sequence space. This idea is related to but distinct from the principles that underlie PSSMs, and is also supported by published data. The second principle is that the binding specificities of antagonistic classes of nucleic acid–binding proteins (e.g., splicing activators and splicing repressors) will tend to avoid binding closely related sequences. This principle is not directly related to principles underlying PSSMs. Whether the differing principles behind PSSMs and NI favor one over the other may depend on the application.

In the current study, we describe the NI approach in its simplest form, based simply on counts of sequence neighbors, which allows the accurate prediction of novel ESE and ESS motifs. The method could easily be extended to use more quantitative information in the modeling of regulatory sequences. For instance, individual sequences of known activity could be weighted according to their abundance in experimentally determined sequence sets, according to their binding affinity, or according to phylogenetic conservation, which is likely to improve prediction accuracy [36]. Alternatively, a more complicated distance measure could be used, e.g., assigning different weights to mismatches at different positions. With new high-throughput technologies such as genome tiling arrays and their application in chromatin immunoprecipitation experiments [37], increasing amounts of experimental data on regulatory sites will become available, and may provide opportunities for application of NI or related methods to the identification of enhancers and silencers of transcription, translation, or other processes.

## Materials and Methods

### Trusted regulatory element data.

Sets of trusted ESEs and ESSs were obtained from published sources. The trusted ESE set was based on the complete set of 238 RESCUE-ESE hexamers [22] and hexamers that occurred at least four times in the set of PESE octamers [8]. The trusted ESS set was based on the FAS-hex2 set of 176 ESS hexamers [7] and hexamers that occurred at least four times in the set of PESS octamers [8]. The cutoff of four occurrences for the PESE/PESS sets was chosen as the lowest cutoff that produced no overlap between ESE and ESS hexamers derived from PESE and PESS octamers, respectively. The five hexamers that were in common between these initial ESS and ESE sets were omitted from the final sets. A total of 12 RESCUE-ESE, 12 PESS, and eight PESE sequences were tested experimentally in the original studies using splicing reporter constructs. All FAS-ESS decamers were identified experimentally; 14 FAS-ESS decamers were further tested in a heterologous exon construct, and six FAS-ESS hexamers were also tested singly and in overlapping pairs. Sequence neighborhoods were visualized using GraphViz (http://www.research.att.com/sw/tools/graphviz) and its Perl bindings (http://search.cpan.org/~lbrocard/GraphViz-2.02).

### NI scoring function and cross-validation.

The training data for NI consist of two lists, A and B, containing nucleotide sequences of length *k* (*k*-mers) that are known members of two antagonistic classes of regulatory elements (e.g., ESE and ESS hexamers). For some applications, a single list (A) can be used, i.e., the list B can be empty. The distance *d_ij_* between a pair of *k*-mers *k_i_* and *k_j_* was measured as the minimum of the Hamming distance over all possible shifts relative to each other plus the size of the shift, defined as

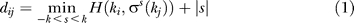
where σ*^s^*(*k_j_*) represents the *k*-mer *k_j_* shifted to the left by *s* bases (where negative values of *s* correspond to shifts to the right by |*s*| bases), and *H* is the ordinary Hamming distance (number of mismatches) between the two (possibly shifted) *k*-mers. No gaps are allowed. For example, the distance between the hexamers GAAGGC and AGAATG would be two, representing the number of mismatches (one, underlined) plus the size of the shift (one base) in the optimal alignment of these two hexamers.

This distance measure is related to H-measure [38] and has been used previously [7,22]. This distance measure was found to be similar in specificity, but more sensitive in cross-validation experiments than ordinary Hamming distance. The sequence neighborhood *N_i_* of a *k*-mer *k_i_* was defined as the set of all *k*-mers *k_j_* with 0 < *d_ij_* ≤ *d*_max_. The raw score *s_i_* of *N_i_* was defined as

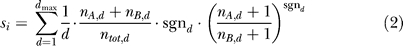
where *n_tot,d_* is the total number of *k*-mers *n_j_* in *N_i_* with *d_ij_* = *d,* and *n_A,d_* and *n_B,d_* are the numbers of *k*-mers *n_j_* in *N_i_* with *d_ij_* = *d* that are contained in the lists A and B, respectively. Using (*n_A,d_* + *n_B,d_*)/*n_tot,d_* as a multiplier rewards neighborhoods that contain many *k*-mers of known class and normalizes unequal neighborhood sizes, while the factor 1/*d* reduces contributions from more distant *k*-mers in a neighborhood. If *n_A,d_* > *n_B,d_,* sgn*_d_* is one; if *n_A,d_* < *n_B,d_,* sgn*_d_* is −1; otherwise sgn*_d_* is zero. For convenience, a normalized score *S_i_* in the interval [−1, +1] was obtained by sigmoid transformation of *s_i_:*

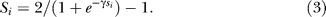
By definition, *k*-mers from lists A and B used in a particular analysis have normalized scores *S_i_* of +1 and −1, respectively. For prediction of ESE/ESS hexamers, we chose *d*_max_ = 1 and γ = 1.3 based on their empirical performance in cross-validation experiments. *N*-fold cross-validation was performed by randomly splitting the sets of trusted elements into *N* parts, and scoring each of these parts by using the remaining *N* − 1 parts as training data, i.e., using the remaining *N* − 1 parts to define the A and B sets for this analysis.

As no trusted set of splicing-neutral sequences was available, trusted ESEs were defined as positives and trusted ESSs as negatives for assessment of cross-validation accuracy. (Accuracy could also be measured by defining ESSs as positives and ESEs as negatives, with the result that the sensitivity and specificity values below would be reversed.) By focusing only on the “trusted” sequences, this approach avoids having to make assumptions about the splicing regulatory activity of the remaining sequences that are neither trusted ESEs nor trusted ESSs. Accuracy of cross-validated NI predictions was then calculated for various score cutoffs *c,* leading to the following definitions: true positive rate (sensitivity) = TP/(TP + FN), where TP is the number of trusted ESEs with *S_i_* ≥ *c,* and FN is the number of trusted ESEs with *S_i_* < *c;* and false positive rate (1 − specificity) = 1 − TN/(TN + FP), where TN is the number of trusted ESSs with *S_i_* < *c,* and FP is defined as the number of trusted ESSs with *S_i_* ≥ *c.*

### Retrospective analysis of NI predictions.

Pairs of mutant and wild-type sequences were extracted from the literature together with experimental measures of their splicing regulatory activity. The effect of mutations on splicing regulators was predicted by assigning a score Δ_max_ to each pair of sequences, calculated as the NI score difference of largest magnitude between corresponding hexamers in the wild-type and the mutant sequences. For example, if the wild-type sequence was AACCGGGTTAA, and the mutant was AACCGAGTTAA (the mutant base is underlined), then the magnitude of Δ_max_ would be defined as the maximum of the absolute differences: |NI(AACCGG) − NI(AACCGA)|, |NI(ACCGGG) − NI(ACCGAG)|,…, |NI(GGTTAA) − NI(AGTTAA)|, where NI(X) represents the NI score of the hexamer X, and the sign of Δ_max_ would be the sign of the difference with largest absolute value. Using mean or median score differences instead of Δ_max_ had only minor effects on the results. Δ_max_ was chosen because it seems more likely to capture the change in activity resulting from mutating the core of a motif, whose location is not known in most cases.

### Hexamer enrichment scores.

Enrichment scores of hexamers in exons versus introns (ΔEI) and close to weak versus strong splice sites (ΔWS) were calculated as described [22], using an updated set of sequences, and a maximum entropy model to score splice sites instead of the original weight matrix model [39]. In brief, sequences of constitutive internal exons and major spliceosome (U2-type) introns longer than 60 nucleotides were collected. Four additional sets of sequences were generated from exons, containing the sequences with the weakest (lowest 25%) or strongest (highest 25%) of 3′ and 5′ splice site scores. At most 200 nucleotides beyond the splice site were analyzed in each sequence, and splice site regions (−5 to +5 for the 5′ splice site and −20 to +5 for the 3′ splice site) were excluded from analysis. Enrichment scores were then calculated for each hexamer according to the following formula:

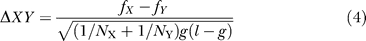
with

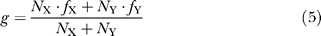
where X and Y are the two sets to be compared (exons/introns, weak/strong 3′ splice site exons, or weak/strong 5′ splice site exons), *f*_X_ is the frequency of a hexamer in set X, defined as number of occurrences per nucleotide, and *N*_X_ is the number of nucleotide positions that were analyzed in set X. For classification of putative ESEs, a minimum cutoff value of 2.5 was used for both ΔEI and ΔWS.

### Splicing reporter assay.

The splicing reporter construct, termed NhexT (for NI hexamer test), was derived from plasmid 4.11.12muC [4], kindly provided by T. Cooper. This construct is a β-globin–derived minigene from which the translation start codon has been deleted to avoid reading frame–related effects on mRNA stability. The second exon in this construct is a test exon whose inclusion rate depends on its length and ESE/ESS content. This exon was shortened from 34 to 30 nucleotides in order to obtain an exon inclusion rate of close to 50% in our HeLa cell line. Cloning of test hexamers into the reporter construct was carried out as follows. Oligonucleotide primers containing the desired mutations were used to amplify the mutation-containing replica of a methylated wild-type plasmid. After digestion with the methylation-sensitive restriction enzyme DpnI, the PCR products were transformed into Escherichia coli DH5α strain. Colonies were picked and cultured, plasmids were purified, and inserts were confirmed by sequencing. Thus, the test hexamer replaced a six-base segment in the test exon at positions 7–12, resulting in no change in exon length. As expected, no correlation was observed between presence of potential termination codons in the test sequence and the corresponding exon two inclusion level.

Primers used for this analysis were as follows. For mutagenesis of the NhexT minigene, forward primer: 5′-ATTTTCCCACCCTTAGGTCGACNNNNNNTACCGCGAATGG-3′ (N denotes inserted nucleotides—unique for each inserted hexamer), reverse primer: 5′-GTCGACCTAAGGGTGGGAAAATAGACCAATAGGC-3′. For PCR and sequencing, forward primer: 5′-AGAACCTCTGGGTCCAAGGGTAG-3′, reverse primer: 5′-CATTCACCACATTGGTGTGC-3′.

### Transfection, RNA isolation, and RT–PCR amplification.

HeLa cells were cultured in Dulbecco's modified Eagle's medium, supplemented with 4.5 g/ml glucose and 10% fetal bovine serum (HyClone, South Logan, Utah, United States). Cells were cultured in six-well plates at 37 °C in a humidified atmosphere with 5% CO_2_. Cells were grown to 80% confluence and transfection was performed using Lipofectamine 2000 (Invitrogen. Carlsbad, California, United States) and 0.5 μg of plasmid DNA according to manufacturer's protocol. Cells were harvested after 48 h and total RNA was extracted using Trizol (Invitrogen), followed by a 1-h treatment with RNase-free DNase. Reverse transcription was performed with SuperScriptIII (Invitrogen) on 2 μg of total RNA for 1 h at 50 °C. The spliced mRNA products derived from the expressed minigene were detected by RT-PCR, using primers described by Coulter and coworkers [4] and a mix of cold dNTPs and ^32^P-dCTP. Amplification was performed with Taq DNA polymerase and supplied buffer (Invitrogen) for 20 cycles, with temperatures of 94 °C, 55 °C, and 72 °C for 30 s each. The products were resolved by 10% polyacrylamide gel electrophoresis in TBE buffer. Agarose gel electrophoresis was used for the purpose of extracting PCR products for confirmation by DNA sequencing. Quantitation of the level of inclusion of exon two was carried out using a 445 SI Phosphorimager (Molecular Dynamics, Sunnyvale, California, United States). Exon inclusion level was calculated as the background-corrected integrated intensity of the exon two–including band divided by the sum of the intensities of the exon two–including and exon two–skipping bands.

### Conservation of ESEs.

Conservation was measured by comparing all occurrences of a *k*-mer sequence in Ensembl (release 27)–annotated coding sequences [40] of the human genome to homologous sequences in the mouse, rat, and dog genomes as defined by available multi-genome alignments (eight-way multiple alignment on hg17, July 2004, downloaded from http://hgdownload.cse.ucsc.edu/goldenPath/hg17/multiz8way) [41,42]. Conservation of ESEs was measured across mammalian genomes using two measures: sequence conservation score (normalized fraction of aligned sequences perfectly conserved) and functional conservation score (normalized fraction of aligned sequences with conserved NI score), described below.

The conservation rate for a *k*-mer was defined as *c*_rate_ = *n*_cons_/*n*_total_, where *n*_cons_ was the number of occurrences of the *k*-mer in aligned positions perfectly conserved across human/mouse/rat/dog exons, and *n*_total_ was the total number of human exonic occurrences in the whole-genome alignment. *c*_rate_ was normalized to obtain the conservation score *c*_score_ as described by Xie and coworkers [43], so that *c*_score_ represents the number of standard deviations by which the observed conservation rate of a *k*-mer exceeds the expected conservation rate obtained by random sampling of 1,000 genomic locations of the given *k*-mer. By including occurrences of the *k*-mer in all three phases in these calculations (i.e., 123/123, 23/123/1, and 3/123/12, where numbers represent positions within codons, and slashes represent boundaries between codons), biases related to protein coding function were diluted. Using this simple measure, the RESCUE-ESE hexamers were among the most conserved and the FAS-hex2 hexamers were among the least conserved hexamers in constitutive exons, suggesting that for this application protein coding effects are sufficiently diluted to reveal effects related to splicing with this measure.

To calculate the functional conservation rate, we considered a human *k*-mer with NI score *S_h_* to be functionally conserved if the NI scores *S_i_* of aligned *k*-mers from the other mammals (mouse, rat, and dog) all fulfilled the condition: |*S_h_* − *S_i_*| ≤ 0.1. The functional conservation score was derived from the functional conservation rate by the same procedure as for the sequence conservation score, i.e., sampling 1,000 random genomic locations of the given *k*-mer and asking what fraction of them were functionally conserved, i.e., had NI scores within 0.1 of the given *k*-mer.

### Conservation of ESS hexamers in alternative splice site exons.

Sets of putative orthologous A5Es and A3Es in the human, dog, mouse, and rat genomes were obtained from a recently published study [20]. A total of 1,074 A5Es and 1,318 A3Es passed the filtering procedures used in that study. To analyze the conservation of ESS hexamers in A5E and A3E extension regions, we used the COR measure, introduced by [20] and described below for completeness. The COR measure is defined as

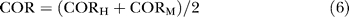
where COR_H_ and COR_M_ are measures of conservation of human and mouse oligonucleotide sequence sets, respectively. COR_H_ and COR_M_ are defined as follows:

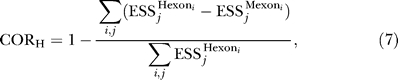
where the upper sum is taken over all *i*, *j* pairs such that

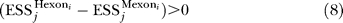
and

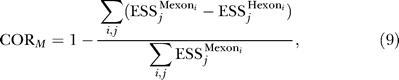
where the upper sum is taken over all *i*, *j* pairs such that

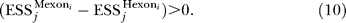
Here, 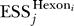 represents the number of occurrences of the *j*th ESS hexamer in the *i*th human exon region, and 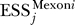 represents the number of occurrences of this hexamer in the corresponding region of the mouse ortholog of the *i*th human exon. In Figure 5C the regions under consideration were the regions between alternative 5′ splice site pairs in sets of orthologous A5E human/mouse exon pairs. Figure 5D shows the analysis of the regions between alternative 3′ splice site pairs. The difference-in-occurrence counts are summed as indicated over all ESS hexamers and all pairs of orthologous exons. Note that this definition is “alignment-independent” in that, in order to achieve the maximum COR value of 1.0, it is sufficient that the counts of the set of hexamers be the same in the corresponding human/mouse exon regions, but it is not required that these hexamers be aligned. This definition is related to alignment-based metrics such as the “conservation rate” [43], but may be more appropriate for splicing regulatory elements that are relatively short and can function at various positions within an exon. For the background distribution, COR values were calculated for random control sets of hexamers that had exactly the same total number of occurrences as the ESSs in the A5E and A3E extension region.

## Supporting Information

Figure S1 Two-Fold Cross-Validation of NI-Predicted Exonic Splicing Regulators (A–C) Cross-validation was performed, using only ESS (A), only ESE (B), or both ESS and ESE trusted hexamers (C) as training data.
(D) Comparison of NI performance in different cross-validation experiments.
(67 KB PDF)

Figure S2 Distribution of Predicted ESE and ESS Hexamers Relative to Splice Sites The mean relative frequency is plotted for different sets of predicted ESE or ESS sequences as a function of distance from the 5′ and 3′ splice sites of human exons. For each hexamer, the frequency was calculated at each position, and the mean frequency was calculated for each hexamer set, then divided by 4^−6^ to obtain mean relative frequency. The “New ESE” and “New ESS” sets are NI-predicted ESEs and ESSs at score cutoffs of 0.8 and −0.8, respectively.
(544 KB PDF)

Figure S3 Retrospective Tests of NI Predictions Pairs of functional and mutated exonic splicing regulators—RESCUE-ESE (squares) [22], PESE/PESS (circles) [8], and FAS-ESS (triangles) [7]—were scored by NI and NI scores were compared to their experimentally determined activity. The maximum change in NI score (*x*-axis) caused by a mutant was plotted against the change in exon inclusion rate (*y*-axis). For FAS-ESS sequences, the percentage of green fluorescent protein–expressing cells was interpreted as exon inclusion rate. The solid line represents a linear fit to the data.
(5 KB EPS)

Figure S4 Comparison of NI- and RESCUE-ESE-Predicted ESEs For each hexamer, the scatter plots show the enrichment in exons versus introns (ΔEI, *x*-axis) and the enrichment in exons with weak splice sites versus exons with strong splice sites (ΔWS, *y*-axis), as described by the original RESCUE-ESE method [22]. ΔWS values for 3′ splice sites are shown in (A), and for 5′ splice sites in (B). “RESCUE-ESE” were predicted to have ESE activity for at least one splice site [22], “trusted ESE” were used as NI training data, “new 3′/5′ RESCUE-ESE” fulfill the conditions ΔEI ≥ 2.5 and ΔWS ≥ 2.5, but were not in the original RESCUE-ESE set, “new NI predicted ESE” have NI scores ≥ 0.8, and “experimentally tested” were selected for testing in a splicing reporter assay (Figure 4). For better visibility, the symbols for “trusted ESE” have been plotted last (i.e., on top of earlier printed symbols), while in Figures 3A and 3B, symbols for NI-predicted hexamers were plotted last.
(838 KB PDF)

Table S1 NI Scores for All Hexanucleotides (3.3 MB DOC)

## Abbreviations

A3E: alternative 3′ splice site exon
A5E: alternative 5′ splice site exon
AUC: area under curve
COR: conserved occurrence rate
ESE: exonic splicing enhancer
ESS: exonic splicing silencer
NI: Neighborhood Inference
PSSM: position-specific scoring matrix
