# Supplementary figures and images for: Inference of Splicing Regulatory Activities by Sequence Neighborhood Analysis
Source: PLoS Genet. 2006 Nov 24;2(11):e191. doi: 10.1371/journal.pgen.0020191 (PMC1657047; doi:10.1371/journal.pgen.0020191)

Supporting Figure 1

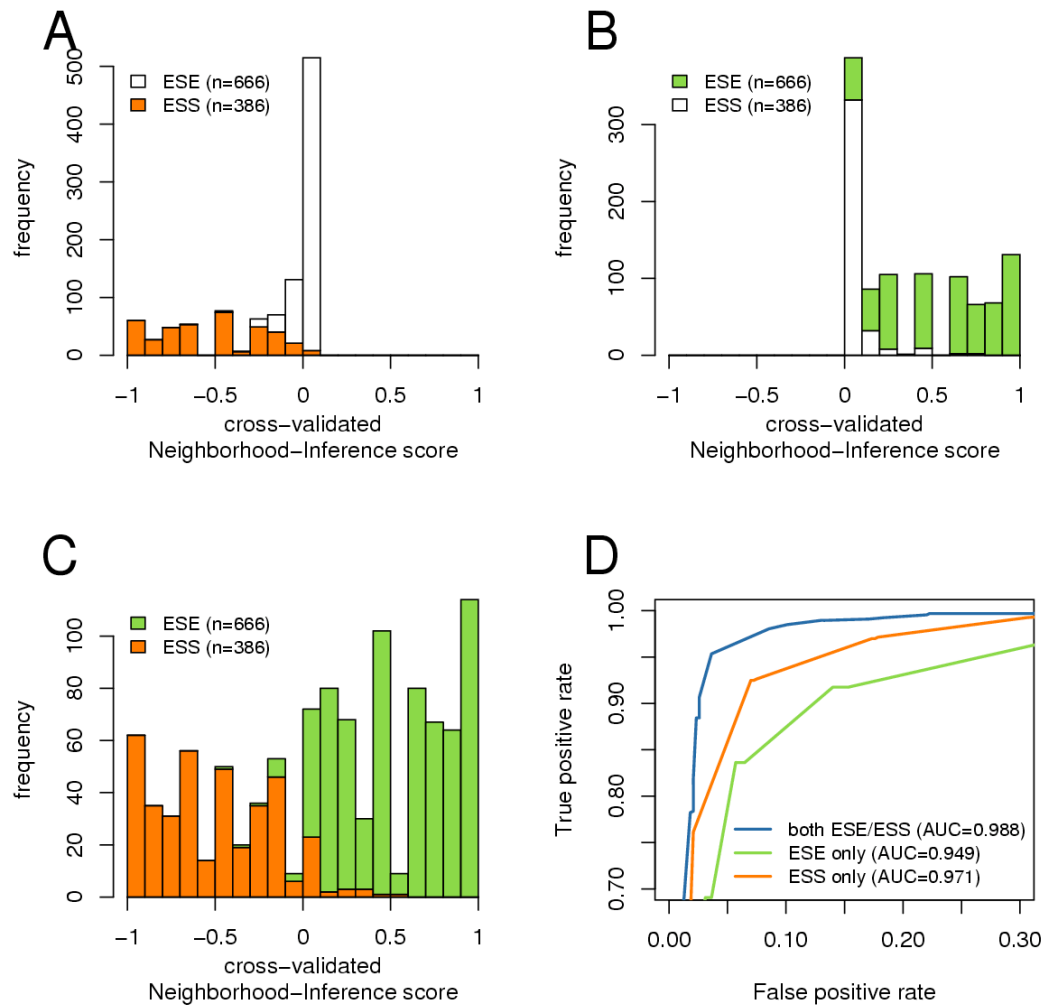

Supplement: Figure S1 — (A–C) Cross-validation was performed, using only ESS (A), only ESE (B), or both ESS and ESE trusted hexamers (C) as training data. (D) Comparison of NI performance in different cross-validation experiments. (67 KB PDF) [file pgen.0020191.sg001.pdf]

Supporting Figure 2

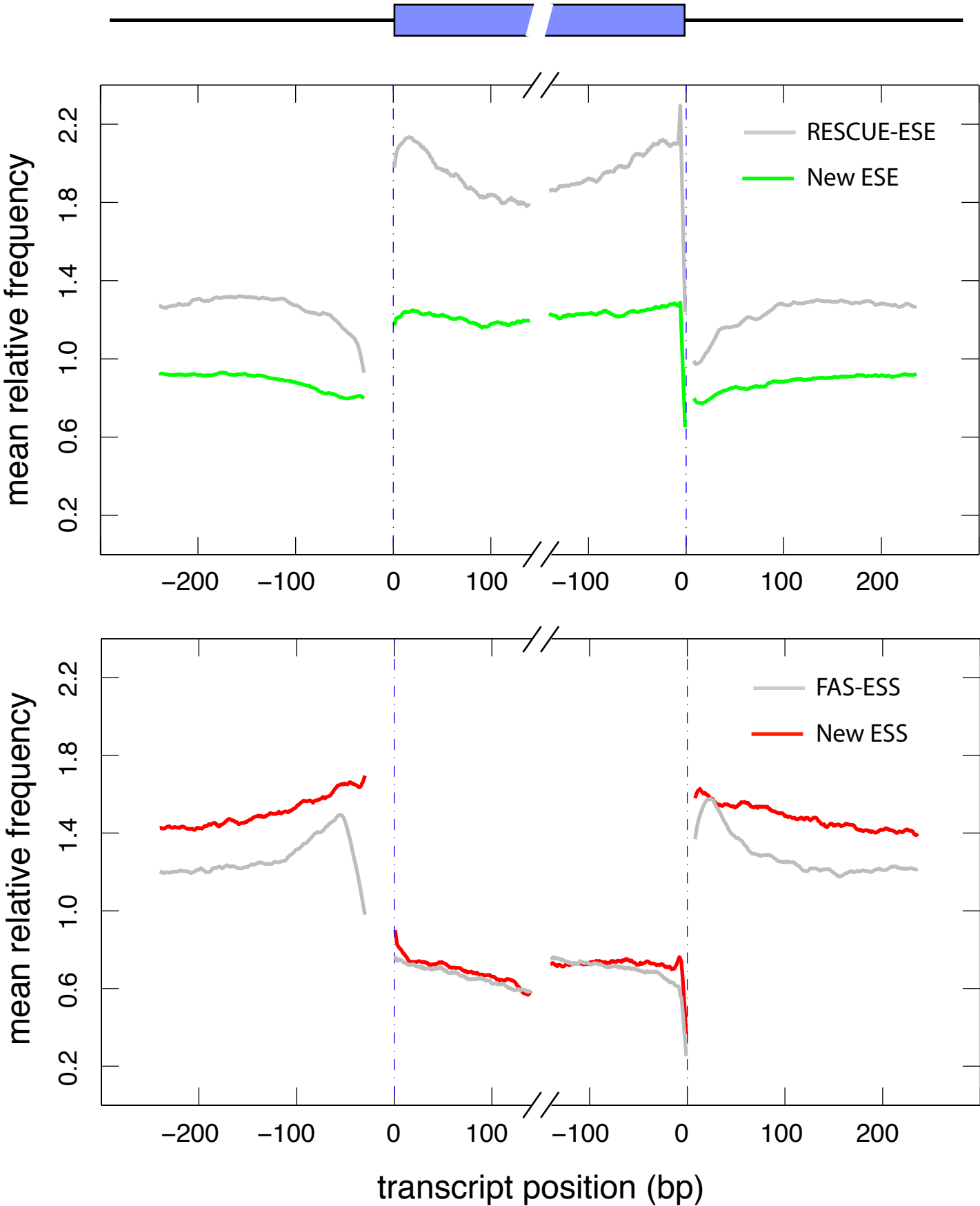

Supplement: Figure S2 — The mean relative frequency is plotted for different sets of predicted ESE or ESS sequences as a function of distance from the 5′ and 3′ splice sites of human exons. For each hexamer, the frequency was calculated at each position, and the mean frequency was calculated for each hexamer set, then divided by 4−6 to obtain mean relative frequency. The “New ESE” and “New ESS” sets are NI-predicted ESEs and ESSs at score cutoffs of 0.8 and −0.8, respectively. (544 KB PDF) [file pgen.0020191.sg002.pdf]

**A**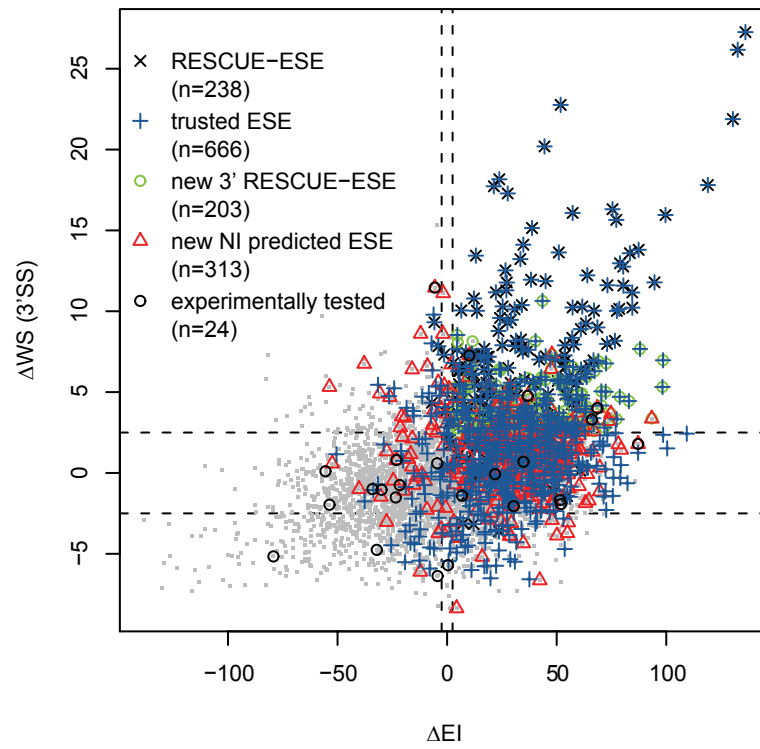**B**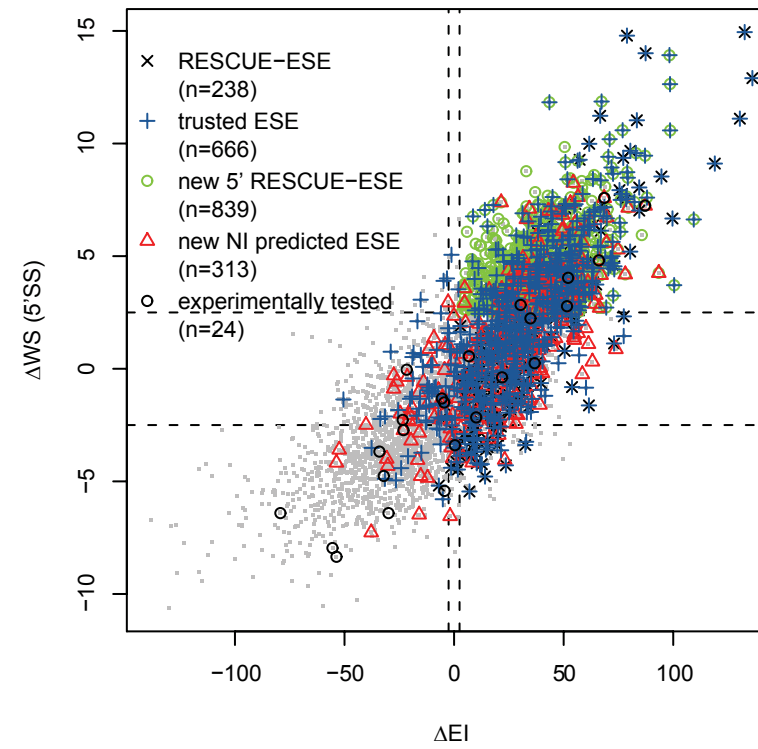

Supplement: Figure S4 — For each hexamer, the scatter plots show the enrichment in exons versus introns (ΔEI, x-axis) and the enrichment in exons with weak splice sites versus exons with strong splice sites (ΔWS, y-axis), as described by the original RESCUE-ESE method [22]. ΔWS values for 3′ splice sites are shown in (A), and for 5′ splice sites in (B). “RESCUE-ESE” were predicted to have ESE activity for at least one splice site [22], “trusted ESE” were used as NI training data, “new 3′/5′ RESCUE-ESE” fulfill the conditions ΔEI ≥ 2.5 and ΔWS ≥ 2.5, but were not in the original RESCUE-ESE set, “new NI predicted ESE” have NI scores ≥ 0.8, and “experimentally tested” were selected for testing in a splicing reporter assay (Figure 4). For better visibility, the symbols for “trusted ESE” have been plotted last (i.e., on top of earlier printed symbols), while in Figures 3A and 3B, symbols for NI-predicted hexamers were plotted last. (838 KB PDF) [file pgen.0020191.sg004.pdf]
