# Supplementary material for: Inference of Splicing Regulatory Activities by Sequence Neighborhood Analysis
Source: PLoS Genet. 2006 Nov 24;2(11):e191. doi: 10.1371/journal.pgen.0020191 (PMC1657047; doi:10.1371/journal.pgen.0020191)
Supplement: Table S1 — (3.3 MB DOC) [file pgen.0020191.st001.doc]

**Supporting Table 1: NI scores for all hexanucleotides**

| **6-mer** | **NI score** | **Trusted ESE/ESS** |
| --- | --- | --- |
| AAAAAA | -1 | 1 |
| AAAAAC | 0.7479 | 0 |
| AAAAAG | 1 | 1 |
| AAAAAT | -1 | 1 |
| AAAACA | 0.8617 | 0 |
| AAAACC | 1 | 1 |
| AAAACG | 0.9074 | 0 |
| AAAACT | 0.9712 | 0 |
| AAAAGA | 1 | 1 |
| AAAAGC | 1 | 1 |
| AAAAGG | 0.671 | 0 |
| AAAAGT | 0.6351 | 0 |
| AAAATA | -1 | 1 |
| AAAATC | 0.493 | 0 |
| AAAATG | -1 | 1 |
| AAAATT | -0.4522 | 0 |
| AAACAA | 0.9414 | 0 |
| AAACAC | 0.7668 | 0 |
| AAACAG | 1 | 1 |
| AAACAT | 0.5503 | 0 |
| AAACCA | 1 | 1 |
| AAACCC | 0.9948 | 0 |
| AAACCG | 0.9468 | 0 |
| AAACCT | 1 | 1 |
| AAACGA | 1 | 1 |
| AAACGC | 0.3027 | 0 |
| AAACGG | 0.5459 | 0 |
| AAACGT | 0.1974 | 0 |
| AAACTA | 0.5503 | 0 |
| AAACTC | 1 | 1 |
| AAACTG | 1 | 1 |
| AAACTT | 0.6351 | 0 |
| AAAGAA | 1 | 1 |
| AAAGAC | 1 | 1 |
| AAAGAG | 1 | 1 |
| AAAGAT | 1 | 1 |
| AAAGCA | 1 | 1 |
| AAAGCC | 0.9973 | 0 |
| AAAGCG | 0.978 | 0 |
| AAAGCT | 1 | 1 |
| AAAGGA | 1 | 1 |
| AAAGGC | 0.3027 | 0 |
| AAAGGG | -0.2326 | 0 |
| AAAGGT | -0.2153 | 0 |
| AAAGTA | 0.5717 | 0 |
| AAAGTC | 0.3027 | 0 |
| AAAGTG | 1 | 1 |
| AAAGTT | 0.2636 | 0 |
| AAATAA | -1 | 1 |
| AAATAC | 0 | 0 |
| AAATAG | -0.3364 | 0 |
| AAATAT | -1 | 1 |
| AAATCA | 0.6351 | 0 |
| AAATCC | 1 | 1 |
| AAATCG | 0.112 | 0 |
| AAATCT | 0.3746 | 0 |
| AAATGA | -1 | 1 |
| AAATGC | -1 | 1 |
| AAATGG | -1 | 1 |
| AAATGT | -1 | 1 |
| AAATTA | -0.8854 | 0 |
| AAATTC | 0.1974 | 0 |
| AAATTG | 0.1651 | 0 |
| AAATTT | -0.8136 | 0 |
| AACAAA | 0.7818 | 0 |
| AACAAC | 1 | 1 |
| AACAAG | 1 | 1 |
| AACAAT | 0.5459 | 0 |
| AACACA | 0.3364 | 0 |
| AACACC | 1 | 1 |
| AACACG | 0.8854 | 0 |
| AACACT | 0.7818 | 0 |
| AACAGA | 1 | 1 |
| AACAGC | 1 | 1 |
| AACAGG | 0.9973 | 0 |
| AACAGT | 0.7818 | 0 |
| AACATA | 0 | 0 |
| AACATC | 0.9973 | 0 |
| AACATG | 1 | 1 |
| AACATT | 0.4219 | 0 |
| AACCAA | 1 | 1 |
| AACCAC | 0.9468 | 0 |
| AACCAG | 1 | 1 |
| AACCAT | 0.7668 | 0 |
| AACCCA | 1 | 1 |
| AACCCC | 0.4942 | 0 |
| AACCCG | 0.8854 | 0 |
| AACCCT | 1 | 1 |
| AACCGA | 1 | 1 |
| AACCGC | 0.1489 | 0 |
| AACCGG | 0.8483 | 0 |
| AACCGT | 0.112 | 0 |
| AACCTA | 0.664 | 0 |
| AACCTC | 0.7668 | 0 |
| AACCTG | 1 | 1 |
| AACCTT | -1 | 1 |
| AACGAA | 1 | 1 |
| AACGAC | 1 | 0 |
| AACGAG | 1 | 1 |
| AACGAT | 0.9074 | 0 |
| AACGCA | 0.4219 | 0 |
| AACGCC | 0.04996 | 0 |
| AACGCG | 0.04996 | 0 |
| AACGCT | 0.1974 | 0 |
| AACGGA | 1 | 1 |
| AACGGC | 0.1489 | 0 |
| AACGGG | 0.1651 | 0 |
| AACGGT | 0.112 | 0 |
| AACGTA | 0 | 0 |
| AACGTC | 0 | 0 |
| AACGTG | 0.8854 | 0 |
| AACGTT | -0.1489 | 0 |
| AACTAA | 0.2449 | 0 |
| AACTAC | 1 | 1 |
| AACTAG | 0.3746 | 0 |
| AACTAT | -0.1651 | 0 |
| AACTCA | 0.4621 | 0 |
| AACTCC | 0.9468 | 0 |
| AACTCG | 0.2913 | 0 |
| AACTCT | 1 | 1 |
| AACTGA | 1 | 1 |
| AACTGC | 0.8483 | 0 |
| AACTGG | 1 | 1 |
| AACTGT | 0.664 | 0 |
| AACTTA | 0.3027 | 0 |
| AACTTC | 1 | 1 |
| AACTTG | 0.9468 | 0 |
| AACTTT | 0.2153 | 0 |
| AAGAAA | 1 | 1 |
| AAGAAC | 1 | 1 |
| AAGAAG | 1 | 1 |
| AAGAAT | 1 | 1 |
| AAGACA | 1 | 1 |
| AAGACC | 1 | 1 |
| AAGACG | 1 | 1 |
| AAGACT | 1 | 1 |
| AAGAGA | 1 | 1 |
| AAGAGC | 0.9919 | 0 |
| AAGAGG | 1 | 1 |
| AAGAGT | 0.9468 | 0 |
| AAGATA | 1 | 1 |
| AAGATC | 1 | 1 |
| AAGATG | 1 | 1 |
| AAGATT | 0.8005 | 0 |
| AAGCAA | 1 | 1 |
| AAGCAC | 0.9919 | 0 |
| AAGCAG | 1 | 1 |
| AAGCAT | 0.6351 | 0 |
| AAGCCA | 1 | 1 |
| AAGCCC | 0.9468 | 0 |
| AAGCCG | 1 | 1 |
| AAGCCT | 0.9973 | 0 |
| AAGCGA | 1 | 1 |
| AAGCGC | 0.4621 | 0 |
| AAGCGG | 1 | 1 |
| AAGCGT | 0.4621 | 0 |
| AAGCTA | 1 | 1 |
| AAGCTC | 1 | 1 |
| AAGCTG | 1 | 1 |
| AAGCTT | 0.3746 | 0 |
| AAGGAA | 1 | 1 |
| AAGGAC | 1 | 1 |
| AAGGAG | 1 | 1 |
| AAGGAT | 1 | 1 |
| AAGGCA | 0.2153 | 0 |
| AAGGCC | 0.2913 | 0 |
| AAGGCG | 0.3027 | 0 |
| AAGGCT | 0.3027 | 0 |
| AAGGGA | 0.537 | 0 |
| AAGGGC | 0 | 0 |
| AAGGGG | -1 | 1 |
| AAGGGT | -0.7668 | 0 |
| AAGGTA | -1 | 1 |
| AAGGTC | 0.2153 | 0 |
| AAGGTG | 0 | 0 |
| AAGGTT | -0.7243 | 0 |
| AAGTAA | 0.4136 | 0 |
| AAGTAC | 1 | 1 |
| AAGTAG | 1 | 1 |
| AAGTAT | -1 | 1 |
| AAGTCA | 0.1974 | 0 |
| AAGTCC | 0.1974 | 0 |
| AAGTCG | 0.3027 | 0 |
| AAGTCT | -0.1651 | 0 |
| AAGTGA | 0.9081 | 0 |
| AAGTGC | 0.2449 | 0 |
| AAGTGG | 1 | 1 |
| AAGTGT | -0.5503 | 0 |
| AAGTTA | -0.5503 | 0 |
| AAGTTC | 0.2636 | 0 |
| AAGTTG | 0.2913 | 0 |
| AAGTTT | -1 | 1 |
| AATAAA | -1 | 1 |
| AATAAC | 0.1651 | 0 |
| AATAAG | 0.3364 | 0 |
| AATAAT | -0.7668 | 0 |
| AATACA | 0 | 0 |
| AATACC | 0.4621 | 0 |
| AATACG | 1 | 1 |
| AATACT | -0.2449 | 0 |
| AATAGA | 0.3364 | 0 |
| AATAGC | 0.1489 | 0 |
| AATAGG | -0.537 | 0 |
| AATAGT | -0.2913 | 0 |
| AATATA | -0.9712 | 0 |
| AATATC | -0.3364 | 0 |
| AATATG | -0.3364 | 0 |
| AATATT | -0.8854 | 0 |
| AATCAA | 1 | 1 |
| AATCAC | 0.6351 | 0 |
| AATCAG | 1 | 1 |
| AATCAT | 0.4219 | 0 |
| AATCCA | 1 | 1 |
| AATCCC | 0.1489 | 0 |
| AATCCG | 0.5459 | 0 |
| AATCCT | 0.5459 | 0 |
| AATCGA | 0.7818 | 0 |
| AATCGC | 0 | 0 |
| AATCGG | 0.1974 | 0 |
| AATCGT | -0.1489 | 0 |
| AATCTA | 0.1651 | 0 |
| AATCTC | 0.1974 | 0 |
| AATCTG | 0.9919 | 0 |
| AATCTT | -0.1974 | 0 |
| AATGAA | 1 | 1 |
| AATGAC | 1 | 1 |
| AATGAG | 0.7163 | 0 |
| AATGAT | -1 | 1 |
| AATGCA | -1 | 1 |
| AATGCC | -0.4219 | 0 |
| AATGCG | -0.4219 | 0 |
| AATGCT | -1 | 1 |
| AATGGA | 1 | 1 |
| AATGGC | -0.3746 | 0 |
| AATGGG | -1 | 1 |
| AATGGT | -1 | 1 |
| AATGTA | -1 | 1 |
| AATGTC | -1 | 1 |
| AATGTG | -0.537 | 0 |
| AATGTT | -1 | 1 |
| AATTAA | -0.6351 | 0 |
| AATTAC | 0.1651 | 0 |
| AATTAG | -0.2449 | 0 |
| AATTAT | -0.8854 | 0 |
| AATTCA | 0.2449 | 0 |
| AATTCC | 0.04996 | 0 |
| AATTCG | 0.112 | 0 |
| AATTCT | -0.3027 | 0 |
| AATTGA | 0.537 | 0 |
| AATTGC | -0.04996 | 0 |
| AATTGG | 0.1651 | 0 |
| AATTGT | -0.7818 | 0 |
| AATTTA | -1 | 1 |
| AATTTC | -0.5459 | 0 |
| AATTTG | -0.6351 | 0 |
| AATTTT | -0.9848 | 0 |
| ACAAAA | -1 | 1 |
| ACAAAC | 0.6351 | 0 |
| ACAAAG | 1 | 1 |
| ACAAAT | -0.2913 | 0 |
| ACAACA | 0.8483 | 0 |
| ACAACC | 0.9973 | 0 |
| ACAACG | 1 | 1 |
| ACAACT | 1 | 1 |
| ACAAGA | 1 | 1 |
| ACAAGC | 1 | 1 |
| ACAAGG | 0.9468 | 0 |
| ACAAGT | 0.978 | 0 |
| ACAATA | 0 | 0 |
| ACAATC | 0.7818 | 0 |
| ACAATG | 0.3364 | 0 |
| ACAATT | 0.1974 | 0 |
| ACACAA | 0.2153 | 0 |
| ACACAC | -1 | 1 |
| ACACAG | 0.3364 | 0 |
| ACACAT | -0.4219 | 0 |
| ACACCA | 0.4621 | 0 |
| ACACCC | 0.1974 | 0 |
| ACACCG | 0.2913 | 0 |
| ACACCT | 0.8854 | 0 |
| ACACGA | 0.8854 | 0 |
| ACACGC | 0 | 0 |
| ACACGG | 0.1489 | 0 |
| ACACGT | 0 | 0 |
| ACACTA | -0.1489 | 0 |
| ACACTC | 0 | 0 |
| ACACTG | 0.7818 | 0 |
| ACACTT | 0 | 0 |
| ACAGAA | 1 | 1 |
| ACAGAC | 0.9712 | 0 |
| ACAGAG | 1 | 1 |
| ACAGAT | 0.6351 | 0 |
| ACAGCA | 0.9468 | 0 |
| ACAGCC | 0.6351 | 0 |
| ACAGCG | 0.4621 | 0 |
| ACAGCT | 0.978 | 0 |
| ACAGGA | 1 | 1 |
| ACAGGC | 0.4621 | 0 |
| ACAGGG | 0.1974 | 0 |
| ACAGGT | 0.1489 | 0 |
| ACAGTA | 0.1489 | 0 |
| ACAGTC | 0.04996 | 0 |
| ACAGTG | 0.6351 | 0 |
| ACAGTT | -0.04996 | 0 |
| ACATAA | -0.537 | 0 |
| ACATAC | -0.6351 | 0 |
| ACATAG | -0.2153 | 0 |
| ACATAT | -1 | 1 |
| ACATCA | 0.7818 | 0 |
| ACATCC | 0.4621 | 0 |
| ACATCG | 0.6351 | 0 |
| ACATCT | 1 | 1 |
| ACATGA | 1 | 1 |
| ACATGC | 0.2913 | 0 |
| ACATGG | 0.3364 | 0 |
| ACATGT | 0 | 0 |
| ACATTA | 0 | 0 |
| ACATTC | 0.1489 | 0 |
| ACATTG | 1 | 1 |
| ACATTT | -0.2153 | 0 |
| ACCAAA | 0.9074 | 0 |
| ACCAAC | 1 | 1 |
| ACCAAG | 1 | 1 |
| ACCAAT | 0.8854 | 0 |
| ACCACA | 0.2913 | 0 |
| ACCACC | 0.8854 | 0 |
| ACCACG | 1 | 1 |
| ACCACT | 0.5459 | 0 |
| ACCAGA | 1 | 1 |
| ACCAGC | 1 | 1 |
| ACCAGG | 0.9712 | 0 |
| ACCAGT | 1 | 1 |
| ACCATA | 0.2913 | 0 |
| ACCATC | 1 | 1 |
| ACCATG | 0.8854 | 0 |
| ACCATT | 0.8854 | 0 |
| ACCCAA | 0.8854 | 0 |
| ACCCAC | 0.4219 | 0 |
| ACCCAG | 1 | 1 |
| ACCCAT | 0.4621 | 0 |
| ACCCCA | 0.04996 | 0 |
| ACCCCC | 0 | 0 |
| ACCCCG | 0.4621 | 0 |
| ACCCCT | 0.4621 | 0 |
| ACCCGA | 0.6351 | 0 |
| ACCCGC | 0.2913 | 0 |
| ACCCGG | 1 | 1 |
| ACCCGT | 0.4621 | 0 |
| ACCCTA | 0.6351 | 0 |
| ACCCTC | 0.8854 | 0 |
| ACCCTG | 1 | 1 |
| ACCCTT | 1 | 1 |
| ACCGAA | 1 | 1 |
| ACCGAC | 1 | 1 |
| ACCGAG | 0.9919 | 0 |
| ACCGAT | 0.4621 | 0 |
| ACCGCA | 0.1489 | 0 |
| ACCGCC | 0.04996 | 0 |
| ACCGCG | 0.04996 | 0 |
| ACCGCT | 0.04996 | 0 |
| ACCGGA | 1 | 1 |
| ACCGGC | 0.8854 | 0 |
| ACCGGG | 0.7818 | 0 |
| ACCGGT | 0.6351 | 0 |
| ACCGTA | 0.1489 | 0 |
| ACCGTC | 0.1489 | 0 |
| ACCGTG | 0.2913 | 0 |
| ACCGTT | -0.112 | 0 |
| ACCTAA | 0.1489 | 0 |
| ACCTAC | 0.4621 | 0 |
| ACCTAG | 0.4621 | 0 |
| ACCTAT | 0.112 | 0 |
| ACCTCA | 0.4621 | 0 |
| ACCTCC | 0.4621 | 0 |
| ACCTCG | 0.8854 | 0 |
| ACCTCT | 1 | 1 |
| ACCTGA | 1 | 1 |
| ACCTGC | 1 | 1 |
| ACCTGG | 1 | 1 |
| ACCTGT | 1 | 1 |
| ACCTTA | 0.1974 | 0 |
| ACCTTC | 0.664 | 0 |
| ACCTTG | 1 | 1 |
| ACCTTT | 0.4219 | 0 |
| ACGAAA | 1 | 1 |
| ACGAAC | 1 | 1 |
| ACGAAG | 1 | 1 |
| ACGAAT | 0.9992 | 0 |
| ACGACA | 0.9973 | 0 |
| ACGACC | 1 | 1 |
| ACGACG | 0.978 | 0 |
| ACGACT | 1 | 1 |
| ACGAGA | 1 | 1 |
| ACGAGC | 0.9919 | 0 |
| ACGAGG | 0.7668 | 0 |
| ACGAGT | 0.7818 | 0 |
| ACGATA | 0.5459 | 0 |
| ACGATC | 1 | 1 |
| ACGATG | 0.7818 | 0 |
| ACGATT | 1 | 1 |
| ACGCAA | 1 | 1 |
| ACGCAC | 0.112 | 0 |
| ACGCAG | 0.4621 | 0 |
| ACGCAT | -0.112 | 0 |
| ACGCCA | 0.1489 | 0 |
| ACGCCC | 0.04996 | 0 |
| ACGCCG | 0.04996 | 0 |
| ACGCCT | 0.1489 | 0 |
| ACGCGA | 0.4621 | 0 |
| ACGCGC | 0 | 0 |
| ACGCGG | 0.2913 | 0 |
| ACGCGT | 0 | 0 |
| ACGCTA | 0.1489 | 0 |
| ACGCTC | 0.2913 | 0 |
| ACGCTG | 0.4621 | 0 |
| ACGCTT | 0.112 | 0 |
| ACGGAA | 0.9998 | 0 |
| ACGGAC | 0.8854 | 0 |
| ACGGAG | 0.9919 | 0 |
| ACGGAT | 0.6351 | 0 |
| ACGGCA | 0 | 0 |
| ACGGCC | 0.04996 | 0 |
| ACGGCG | 0.04996 | 0 |
| ACGGCT | 0.4621 | 0 |
| ACGGGA | 0.7818 | 0 |
| ACGGGC | 0 | 0 |
| ACGGGG | -0.3027 | 0 |
| ACGGGT | -0.1489 | 0 |
| ACGGTA | -0.2913 | 0 |
| ACGGTC | 0.1489 | 0 |
| ACGGTG | 0 | 0 |
| ACGGTT | -0.112 | 0 |
| ACGTAA | 0.1651 | 0 |
| ACGTAC | 0.1489 | 0 |
| ACGTAG | 0.1651 | 0 |
| ACGTAT | -0.3027 | 0 |
| ACGTCA | 0.1974 | 0 |
| ACGTCC | 0.04996 | 0 |
| ACGTCG | 0.4621 | 0 |
| ACGTCT | 0.1974 | 0 |
| ACGTGA | 1 | 1 |
| ACGTGC | 0.4621 | 0 |
| ACGTGG | 1 | 1 |
| ACGTGT | 0.3027 | 0 |
| ACGTTA | -0.112 | 0 |
| ACGTTC | -0.5459 | 0 |
| ACGTTG | 0.2913 | 0 |
| ACGTTT | -0.1974 | 0 |
| ACTAAA | 0 | 0 |
| ACTAAC | 0.2913 | 0 |
| ACTAAG | 0.3027 | 0 |
| ACTAAT | -0.04996 | 0 |
| ACTACA | 1 | 1 |
| ACTACC | 0.2913 | 0 |
| ACTACG | 0.5459 | 0 |
| ACTACT | 0.4621 | 0 |
| ACTAGA | 0.537 | 0 |
| ACTAGC | 0 | 0 |
| ACTAGG | -1 | 1 |
| ACTAGT | -0.112 | 0 |
| ACTATA | 0 | 0 |
| ACTATC | 0.1489 | 0 |
| ACTATG | 0 | 0 |
| ACTATT | -0.1974 | 0 |
| ACTCAA | 0.6351 | 0 |
| ACTCAC | 0 | 0 |
| ACTCAG | 0.6351 | 0 |
| ACTCAT | 0 | 0 |
| ACTCCA | 0.7818 | 0 |
| ACTCCC | 0.04996 | 0 |
| ACTCCG | 0.1974 | 0 |
| ACTCCT | 0.2913 | 0 |
| ACTCGA | 0.6351 | 0 |
| ACTCGC | 0 | 0 |
| ACTCGG | 0.1974 | 0 |
| ACTCGT | -0.04996 | 0 |
| ACTCTA | 0.112 | 0 |
| ACTCTC | 0.1489 | 0 |
| ACTCTG | 1 | 1 |
| ACTCTT | 0.1974 | 0 |
| ACTGAA | 1 | 1 |
| ACTGAC | 1 | 1 |
| ACTGAG | 1 | 1 |
| ACTGAT | 0.664 | 0 |
| ACTGCA | 0.664 | 0 |
| ACTGCC | 0.4621 | 0 |
| ACTGCG | 0.1489 | 0 |
| ACTGCT | 0.3027 | 0 |
| ACTGGA | 1 | 1 |
| ACTGGC | 0.7818 | 0 |
| ACTGGG | 0.2153 | 0 |
| ACTGGT | 0.3027 | 0 |
| ACTGTA | 0.1651 | 0 |
| ACTGTC | 0.112 | 0 |
| ACTGTG | 0.7818 | 0 |
| ACTGTT | -0.3364 | 0 |
| ACTTAA | 0.1974 | 0 |
| ACTTAC | 0 | 0 |
| ACTTAG | -0.2449 | 0 |
| ACTTAT | -0.4621 | 0 |
| ACTTCA | 1 | 1 |
| ACTTCC | 0.4621 | 0 |
| ACTTCG | 1 | 1 |
| ACTTCT | 0.4355 | 0 |
| ACTTGA | 1 | 1 |
| ACTTGC | 0.1489 | 0 |
| ACTTGG | 0.4355 | 0 |
| ACTTGT | -0.1651 | 0 |
| ACTTTA | -0.4355 | 0 |
| ACTTTC | -0.2913 | 0 |
| ACTTTG | 0.2449 | 0 |
| ACTTTT | -0.6351 | 0 |
| AGAAAA | 1 | 1 |
| AGAAAC | 1 | 1 |
| AGAAAG | 1 | 1 |
| AGAAAT | 0.8393 | 0 |
| AGAACA | 1 | 1 |
| AGAACC | 1 | 1 |
| AGAACG | 1 | 1 |
| AGAACT | 1 | 1 |
| AGAAGA | 1 | 1 |
| AGAAGC | 1 | 1 |
| AGAAGG | 1 | 1 |
| AGAAGT | 1 | 1 |
| AGAATA | 0.4621 | 0 |
| AGAATC | 1 | 1 |
| AGAATG | 1 | 1 |
| AGAATT | 0.7668 | 0 |
| AGACAA | 1 | 1 |
| AGACAC | 1 | 1 |
| AGACAG | 0.9919 | 0 |
| AGACAT | 1 | 1 |
| AGACCA | 1 | 1 |
| AGACCC | 1 | 1 |
| AGACCG | 0.8483 | 0 |
| AGACCT | 1 | 1 |
| AGACGA | 1 | 1 |
| AGACGC | 0.8854 | 0 |
| AGACGG | 0.1651 | 0 |
| AGACGT | 0.8483 | 0 |
| AGACTA | 0.3364 | 0 |
| AGACTC | 0.9919 | 0 |
| AGACTG | 0.978 | 0 |
| AGACTT | 0.5459 | 0 |
| AGAGAA | 1 | 1 |
| AGAGAC | 1 | 1 |
| AGAGAG | 1 | 1 |
| AGAGAT | 1 | 1 |
| AGAGCA | 0.9973 | 0 |
| AGAGCC | 0.7818 | 0 |
| AGAGCG | 0.1489 | 0 |
| AGAGCT | 0.9468 | 0 |
| AGAGGA | 1 | 1 |
| AGAGGC | 1 | 1 |
| AGAGGG | 0.4136 | 0 |
| AGAGGT | 0.9074 | 0 |
| AGAGTA | 0.2153 | 0 |
| AGAGTC | 0.6351 | 0 |
| AGAGTG | 0.4219 | 0 |
| AGAGTT | -0.1651 | 0 |
| AGATAA | 0.3364 | 0 |
| AGATAC | 0.8854 | 0 |
| AGATAG | -0.2636 | 0 |
| AGATAT | 0.4136 | 0 |
| AGATCA | 0.9712 | 0 |
| AGATCC | 1 | 1 |
| AGATCG | 0.3364 | 0 |
| AGATCT | 0.8617 | 0 |
| AGATGA | 1 | 1 |
| AGATGC | 1 | 1 |
| AGATGG | -1 | 1 |
| AGATGT | 1 | 1 |
| AGATTA | -1 | 1 |
| AGATTC | 0.4355 | 0 |
| AGATTG | -0.1651 | 0 |
| AGATTT | -0.3027 | 0 |
| AGCAAA | 1 | 1 |
| AGCAAC | 0.9973 | 0 |
| AGCAAG | 1 | 1 |
| AGCAAT | 0.978 | 0 |
| AGCACA | 0.7818 | 0 |
| AGCACC | 0.8854 | 0 |
| AGCACG | 0.7818 | 0 |
| AGCACT | 1 | 1 |
| AGCAGA | 1 | 1 |
| AGCAGC | 1 | 1 |
| AGCAGG | 1 | 1 |
| AGCAGT | 1 | 1 |
| AGCATA | 0.3364 | 0 |
| AGCATC | 1 | 1 |
| AGCATG | 0.664 | 0 |
| AGCATT | 1 | 1 |
| AGCCAA | 0.7818 | 0 |
| AGCCAC | 0.2913 | 0 |
| AGCCAG | 0.3027 | 0 |
| AGCCAT | 0.3027 | 0 |
| AGCCCA | 0.4621 | 0 |
| AGCCCC | 0.4621 | 0 |
| AGCCCG | 0 | 0 |
| AGCCCT | 1 | 1 |
| AGCCGA | 0.5459 | 0 |
| AGCCGC | 0.1974 | 0 |
| AGCCGG | -1 | 1 |
| AGCCGT | 0.4355 | 0 |
| AGCCTA | 0.4621 | 0 |
| AGCCTC | 1 | 1 |
| AGCCTG | 0.9074 | 0 |
| AGCCTT | 1 | 1 |
| AGCGAA | 0.8854 | 0 |
| AGCGAC | 0.6351 | 0 |
| AGCGAG | 0.7818 | 0 |
| AGCGAT | 0.1489 | 0 |
| AGCGCA | 0 | 0 |
| AGCGCC | 0 | 0 |
| AGCGCG | 0 | 0 |
| AGCGCT | 0.2913 | 0 |
| AGCGGA | 0.978 | 0 |
| AGCGGC | 0.2913 | 0 |
| AGCGGG | 0.1651 | 0 |
| AGCGGT | 0.3027 | 0 |
| AGCGTA | -0.112 | 0 |
| AGCGTC | 0.2913 | 0 |
| AGCGTG | 0 | 0 |
| AGCGTT | 0.1651 | 0 |
| AGCTAA | 0.3027 | 0 |
| AGCTAC | 0.2913 | 0 |
| AGCTAG | 0.2449 | 0 |
| AGCTAT | 0.3027 | 0 |
| AGCTCA | 0.4621 | 0 |
| AGCTCC | 0.6351 | 0 |
| AGCTCG | 0.3027 | 0 |
| AGCTCT | 1 | 1 |
| AGCTGA | 1 | 1 |
| AGCTGC | 0.9973 | 0 |
| AGCTGG | 1 | 1 |
| AGCTGT | 1 | 1 |
| AGCTTA | -0.3364 | 0 |
| AGCTTC | 0.4219 | 0 |
| AGCTTG | 0.1974 | 0 |
| AGCTTT | 0.2913 | 0 |
| AGGAAA | 1 | 1 |
| AGGAAC | 1 | 1 |
| AGGAAG | 1 | 1 |
| AGGAAT | 1 | 1 |
| AGGACA | 1 | 1 |
| AGGACC | 1 | 1 |
| AGGACG | 0.9998 | 0 |
| AGGACT | 1 | 1 |
| AGGAGA | 1 | 1 |
| AGGAGC | 1 | 1 |
| AGGAGG | 1 | 1 |
| AGGAGT | 1 | 1 |
| AGGATA | 0.493 | 0 |
| AGGATC | 1 | 1 |
| AGGATG | 0.7132 | 0 |
| AGGATT | 1 | 1 |
| AGGCAA | 0.3364 | 0 |
| AGGCAC | 0.1974 | 0 |
| AGGCAG | 0.2449 | 0 |
| AGGCAT | -1 | 1 |
| AGGCCA | 0.4621 | 0 |
| AGGCCC | 0.4621 | 0 |
| AGGCCG | 0.1974 | 0 |
| AGGCCT | 0.3027 | 0 |
| AGGCGA | 0.4621 | 0 |
| AGGCGC | 0.1489 | 0 |
| AGGCGG | 0 | 0 |
| AGGCGT | -0.1974 | 0 |
| AGGCTA | 0 | 0 |
| AGGCTC | 1 | 1 |
| AGGCTG | 0.5459 | 0 |
| AGGCTT | -0.3746 | 0 |
| AGGGAA | 0.4355 | 0 |
| AGGGAC | 0.6351 | 0 |
| AGGGAG | 1 | 1 |
| AGGGAT | -0.3103 | 0 |
| AGGGCA | 0 | 0 |
| AGGGCC | 0.04996 | 0 |
| AGGGCG | 0 | 0 |
| AGGGCT | -0.112 | 0 |
| AGGGGA | -0.5717 | 0 |
| AGGGGC | -0.2449 | 0 |
| AGGGGG | -1 | 1 |
| AGGGGT | -1 | 1 |
| AGGGTA | -1 | 1 |
| AGGGTC | -0.2153 | 0 |
| AGGGTG | -1 | 1 |
| AGGGTT | -1 | 1 |
| AGGTAA | -1 | 1 |
| AGGTAC | -0.3364 | 0 |
| AGGTAG | -1 | 1 |
| AGGTAT | -1 | 1 |
| AGGTCA | -0.1651 | 0 |
| AGGTCC | 0.2913 | 0 |
| AGGTCG | -0.2913 | 0 |
| AGGTCT | -0.2449 | 0 |
| AGGTGA | 1 | 1 |
| AGGTGC | 0.1974 | 0 |
| AGGTGG | -1 | 1 |
| AGGTGT | -0.493 | 0 |
| AGGTTA | -1 | 1 |
| AGGTTC | -0.2153 | 0 |
| AGGTTG | -0.8483 | 0 |
| AGGTTT | -1 | 1 |
| AGTAAA | 0.3103 | 0 |
| AGTAAC | 0.2913 | 0 |
| AGTAAG | -0.2636 | 0 |
| AGTAAT | -0.1974 | 0 |
| AGTACA | 0.4219 | 0 |
| AGTACC | 0.2913 | 0 |
| AGTACG | 0 | 0 |
| AGTACT | 0.3027 | 0 |
| AGTAGA | 1 | 1 |
| AGTAGC | 0.4219 | 0 |
| AGTAGG | -1 | 1 |
| AGTAGT | -0.3103 | 0 |
| AGTATA | -1 | 1 |
| AGTATC | -0.2153 | 0 |
| AGTATG | -0.6351 | 0 |
| AGTATT | -0.6351 | 0 |
| AGTCAA | 1 | 1 |
| AGTCAC | 0.2913 | 0 |
| AGTCAG | 0.2449 | 0 |
| AGTCAT | -0.2449 | 0 |
| AGTCCA | 0.3027 | 0 |
| AGTCCC | 0 | 0 |
| AGTCCG | -1 | 1 |
| AGTCCT | 0.2153 | 0 |
| AGTCGA | 0.5459 | 0 |
| AGTCGC | 0 | 0 |
| AGTCGG | -0.4621 | 0 |
| AGTCGT | -1 | 1 |
| AGTCTA | -0.1974 | 0 |
| AGTCTC | 0.112 | 0 |
| AGTCTG | -0.112 | 0 |
| AGTCTT | -0.3027 | 0 |
| AGTGAA | 1 | 1 |
| AGTGAC | 1 | 1 |
| AGTGAG | 1 | 1 |
| AGTGAT | 0.3364 | 0 |
| AGTGCA | 0.2449 | 0 |
| AGTGCC | 0.04996 | 0 |
| AGTGCG | 0.1651 | 0 |
| AGTGCT | -0.1651 | 0 |
| AGTGGA | 1 | 1 |
| AGTGGC | 0.537 | 0 |
| AGTGGG | -0.4136 | 0 |
| AGTGGT | -0.4621 | 0 |
| AGTGTA | -1 | 1 |
| AGTGTC | -0.3364 | 0 |
| AGTGTG | -0.5459 | 0 |
| AGTGTT | -1 | 1 |
| AGTTAA | -0.4355 | 0 |
| AGTTAC | -0.3027 | 0 |
| AGTTAG | -1 | 1 |
| AGTTAT | -0.9973 | 0 |
| AGTTCA | -0.5459 | 0 |
| AGTTCC | -0.664 | 0 |
| AGTTCG | -1 | 1 |
| AGTTCT | -1 | 1 |
| AGTTGA | 0.4355 | 0 |
| AGTTGC | -0.112 | 0 |
| AGTTGG | -0.7243 | 0 |
| AGTTGT | -1 | 1 |
| AGTTTA | -1 | 1 |
| AGTTTC | -1 | 1 |
| AGTTTG | -1 | 1 |
| AGTTTT | -1 | 1 |
| ATAAAA | -1 | 1 |
| ATAAAC | -0.4219 | 0 |
| ATAAAG | -0.3552 | 0 |
| ATAAAT | -1 | 1 |
| ATAACA | -0.112 | 0 |
| ATAACC | 0.1489 | 0 |
| ATAACG | 0.112 | 0 |
| ATAACT | -0.3364 | 0 |
| ATAAGA | 0.4219 | 0 |
| ATAAGC | 0.3027 | 0 |
| ATAAGG | -0.4219 | 0 |
| ATAAGT | -0.5459 | 0 |
| ATAATA | -0.9919 | 0 |
| ATAATC | -0.1974 | 0 |
| ATAATG | -1 | 1 |
| ATAATT | -1 | 1 |
| ATACAA | -0.2636 | 0 |
| ATACAC | -0.1974 | 0 |
| ATACAG | 0 | 0 |
| ATACAT | -1 | 1 |
| ATACCA | 0.112 | 0 |
| ATACCC | 0.04996 | 0 |
| ATACCG | 0 | 0 |
| ATACCT | 0 | 0 |
| ATACGA | 0.3027 | 0 |
| ATACGC | 0 | 0 |
| ATACGG | 0.112 | 0 |
| ATACGT | -0.1974 | 0 |
| ATACTA | -1 | 1 |
| ATACTC | -0.1974 | 0 |
| ATACTG | -0.2449 | 0 |
| ATACTT | -1 | 1 |
| ATAGAA | 0.4621 | 0 |
| ATAGAC | 0.3027 | 0 |
| ATAGAG | 0.2153 | 0 |
| ATAGAT | -1 | 1 |
| ATAGCA | 0.04996 | 0 |
| ATAGCC | 0 | 0 |
| ATAGCG | 0 | 0 |
| ATAGCT | -0.1974 | 0 |
| ATAGGA | 0.2913 | 0 |
| ATAGGC | -0.4219 | 0 |
| ATAGGG | -1 | 1 |
| ATAGGT | -0.9712 | 0 |
| ATAGTA | -0.8854 | 0 |
| ATAGTC | -0.112 | 0 |
| ATAGTG | -0.664 | 0 |
| ATAGTT | -1 | 1 |
| ATATAA | -1 | 1 |
| ATATAC | -0.9468 | 0 |
| ATATAG | -1 | 1 |
| ATATAT | -1 | 1 |
| ATATCA | -0.4219 | 0 |
| ATATCC | 0.2153 | 0 |
| ATATCG | -0.1974 | 0 |
| ATATCT | -1 | 1 |
| ATATGA | -0.3103 | 0 |
| ATATGC | -1 | 1 |
| ATATGG | -0.6469 | 0 |
| ATATGT | -1 | 1 |
| ATATTA | -1 | 1 |
| ATATTC | -0.4219 | 0 |
| ATATTG | -0.664 | 0 |
| ATATTT | -1 | 1 |
| ATCAAA | 1 | 1 |
| ATCAAC | 0.9973 | 0 |
| ATCAAG | 1 | 1 |
| ATCAAT | 1 | 1 |
| ATCACA | 0.1489 | 0 |
| ATCACC | 0.2913 | 0 |
| ATCACG | 0.1489 | 0 |
| ATCACT | 0.1489 | 0 |
| ATCAGA | 1 | 1 |
| ATCAGC | 1 | 1 |
| ATCAGG | 0.9074 | 0 |
| ATCAGT | 0.978 | 0 |
| ATCATA | 0 | 0 |
| ATCATC | 0.7818 | 0 |
| ATCATG | 0.2636 | 0 |
| ATCATT | -0.2913 | 0 |
| ATCCAA | 1 | 1 |
| ATCCAC | 0.4621 | 0 |
| ATCCAG | 0.9919 | 0 |
| ATCCAT | 0.2449 | 0 |
| ATCCCA | 0.1489 | 0 |
| ATCCCC | 0.04996 | 0 |
| ATCCCG | 0.2913 | 0 |
| ATCCCT | 0.1489 | 0 |
| ATCCGA | 0.6351 | 0 |
| ATCCGC | 0.2913 | 0 |
| ATCCGG | 1 | 1 |
| ATCCGT | -0.1651 | 0 |
| ATCCTA | 0.1974 | 0 |
| ATCCTC | 0.8854 | 0 |
| ATCCTG | 1 | 1 |
| ATCCTT | 0 | 0 |
| ATCGAA | 0.9973 | 0 |
| ATCGAC | 0.4621 | 0 |
| ATCGAG | 0.6351 | 0 |
| ATCGAT | 0.1974 | 0 |
| ATCGCA | 0 | 0 |
| ATCGCC | 0.04996 | 0 |
| ATCGCG | 0.04996 | 0 |
| ATCGCT | 0.04996 | 0 |
| ATCGGA | 0.7818 | 0 |
| ATCGGC | 0.6351 | 0 |
| ATCGGG | 0.1651 | 0 |
| ATCGGT | 1 | 1 |
| ATCGTA | -0.1489 | 0 |
| ATCGTC | 0.4621 | 0 |
| ATCGTG | 0.04996 | 0 |
| ATCGTT | -0.5459 | 0 |
| ATCTAA | 0 | 0 |
| ATCTAC | 0.3027 | 0 |
| ATCTAG | -0.1651 | 0 |
| ATCTAT | -0.4219 | 0 |
| ATCTCA | 0.112 | 0 |
| ATCTCC | 1 | 1 |
| ATCTCG | 0.1489 | 0 |
| ATCTCT | -0.3364 | 0 |
| ATCTGA | 1 | 1 |
| ATCTGC | 1 | 1 |
| ATCTGG | 1 | 1 |
| ATCTGT | 0.4522 | 0 |
| ATCTTA | -0.4355 | 0 |
| ATCTTC | 1 | 1 |
| ATCTTG | 0.3027 | 0 |
| ATCTTT | -0.664 | 0 |
| ATGAAA | 1 | 1 |
| ATGAAC | 0.9998 | 0 |
| ATGAAG | 1 | 1 |
| ATGAAT | 0.5717 | 0 |
| ATGACA | 0.3364 | 0 |
| ATGACC | 0.978 | 0 |
| ATGACG | 0.9468 | 0 |
| ATGACT | 0.4219 | 0 |
| ATGAGA | 1 | 1 |
| ATGAGC | 0.7818 | 0 |
| ATGAGG | 0.9081 | 0 |
| ATGAGT | 0.1651 | 0 |
| ATGATA | -1 | 1 |
| ATGATC | 0.537 | 0 |
| ATGATG | 1 | 1 |
| ATGATT | -0.4136 | 0 |
| ATGCAA | 0.3799 | 0 |
| ATGCAC | -0.3027 | 0 |
| ATGCAG | 0.493 | 0 |
| ATGCAT | -1 | 1 |
| ATGCCA | 0.112 | 0 |
| ATGCCC | 0.1489 | 0 |
| ATGCCG | 0.1489 | 0 |
| ATGCCT | -0.2913 | 0 |
| ATGCGA | 0.4621 | 0 |
| ATGCGC | 0 | 0 |
| ATGCGG | 0.4219 | 0 |
| ATGCGT | -0.4621 | 0 |
| ATGCTA | -0.664 | 0 |
| ATGCTC | 0.2913 | 0 |
| ATGCTG | 0.5503 | 0 |
| ATGCTT | -1 | 1 |
| ATGGAA | 1 | 1 |
| ATGGAC | 1 | 0 |
| ATGGAG | 0.9985 | 0 |
| ATGGAT | 1 | 1 |
| ATGGCA | 0 | 0 |
| ATGGCC | 0.4621 | 0 |
| ATGGCG | 1 | 1 |
| ATGGCT | 0.2153 | 0 |
| ATGGGA | -0.4621 | 0 |
| ATGGGC | -0.4219 | 0 |
| ATGGGG | -1 | 1 |
| ATGGGT | -1 | 1 |
| ATGGTA | -1 | 1 |
| ATGGTC | 1 | 1 |
| ATGGTG | -0.3799 | 0 |
| ATGGTT | -1 | 1 |
| ATGTAA | -1 | 1 |
| ATGTAC | -0.5459 | 0 |
| ATGTAG | -0.5964 | 0 |
| ATGTAT | -1 | 1 |
| ATGTCA | -1 | 1 |
| ATGTCC | -0.2449 | 0 |
| ATGTCG | 0 | 0 |
| ATGTCT | -1 | 1 |
| ATGTGA | 0.2636 | 0 |
| ATGTGC | 0.4355 | 0 |
| ATGTGG | 1 | 1 |
| ATGTGT | -1 | 1 |
| ATGTTA | -1 | 1 |
| ATGTTC | -0.537 | 0 |
| ATGTTG | -0.3364 | 0 |
| ATGTTT | -1 | 1 |
| ATTAAA | -1 | 1 |
| ATTAAC | -0.664 | 0 |
| ATTAAG | 0 | 0 |
| ATTAAT | -1 | 1 |
| ATTACA | -0.112 | 0 |
| ATTACC | -0.04996 | 0 |
| ATTACG | 0 | 0 |
| ATTACT | -0.6351 | 0 |
| ATTAGA | 0 | 0 |
| ATTAGC | -0.112 | 0 |
| ATTAGG | -1 | 1 |
| ATTAGT | -0.9973 | 0 |
| ATTATA | -0.978 | 0 |
| ATTATC | -0.1489 | 0 |
| ATTATG | -0.9074 | 0 |
| ATTATT | -1 | 1 |
| ATTCAA | 0.537 | 0 |
| ATTCAC | 0.3027 | 0 |
| ATTCAG | 1 | 1 |
| ATTCAT | -0.7243 | 0 |
| ATTCCA | 0.04996 | 0 |
| ATTCCC | 0 | 0 |
| ATTCCG | -0.1974 | 0 |
| ATTCCT | -0.4219 | 0 |
| ATTCGA | 0.2913 | 0 |
| ATTCGC | 0 | 0 |
| ATTCGG | 0.112 | 0 |
| ATTCGT | -0.7818 | 0 |
| ATTCTA | -0.8854 | 0 |
| ATTCTC | -0.2913 | 0 |
| ATTCTG | 0 | 0 |
| ATTCTT | -0.978 | 0 |
| ATTGAA | 1 | 1 |
| ATTGAC | 1 | 1 |
| ATTGAG | 1 | 1 |
| ATTGAT | 0 | 0 |
| ATTGCA | 0.112 | 0 |
| ATTGCC | 0.04996 | 0 |
| ATTGCG | 0.2913 | 0 |
| ATTGCT | -0.4621 | 0 |
| ATTGGA | 1 | 1 |
| ATTGGC | 0.1489 | 0 |
| ATTGGG | -0.6351 | 0 |
| ATTGGT | -0.2449 | 0 |
| ATTGTA | -0.7243 | 0 |
| ATTGTC | -0.1651 | 0 |
| ATTGTG | -0.1974 | 0 |
| ATTGTT | -1 | 1 |
| ATTTAA | -1 | 1 |
| ATTTAC | -1 | 1 |
| ATTTAG | -0.8617 | 0 |
| ATTTAT | -1 | 1 |
| ATTTCA | -0.664 | 0 |
| ATTTCC | -0.4219 | 0 |
| ATTTCG | -0.3027 | 0 |
| ATTTCT | -1 | 1 |
| ATTTGA | -0.2153 | 0 |
| ATTTGC | -0.4219 | 0 |
| ATTTGG | -0.3746 | 0 |
| ATTTGT | -1 | 1 |
| ATTTTA | -1 | 1 |
| ATTTTC | -0.8483 | 0 |
| ATTTTG | -0.978 | 0 |
| ATTTTT | -1 | 1 |
| CAAAAA | 0.632 | 0 |
| CAAAAC | 1 | 1 |
| CAAAAG | 1 | 1 |
| CAAAAT | 0.3799 | 0 |
| CAAACA | 1 | 1 |
| CAAACC | 0.9468 | 0 |
| CAAACG | 0.4219 | 0 |
| CAAACT | 1 | 1 |
| CAAAGA | 1 | 1 |
| CAAAGC | 0.8854 | 0 |
| CAAAGG | 0.5459 | 0 |
| CAAAGT | 0.6351 | 0 |
| CAAATA | -0.4621 | 0 |
| CAAATC | 0.3027 | 0 |
| CAAATG | -1 | 1 |
| CAAATT | 0.112 | 0 |
| CAACAA | 0.978 | 0 |
| CAACAC | 0.7818 | 0 |
| CAACAG | 0.9468 | 0 |
| CAACAT | 0.7818 | 0 |
| CAACCA | 1 | 1 |
| CAACCC | 0.8854 | 0 |
| CAACCG | 0.2913 | 0 |
| CAACCT | 0.9074 | 0 |
| CAACGA | 1 | 1 |
| CAACGC | 0.2913 | 0 |
| CAACGG | 0.4621 | 0 |
| CAACGT | 0.6351 | 0 |
| CAACTA | 0.4355 | 0 |
| CAACTC | 0.6351 | 0 |
| CAACTG | 0.9074 | 0 |
| CAACTT | 1 | 1 |
| CAAGAA | 1 | 1 |
| CAAGAC | 1 | 1 |
| CAAGAG | 0.9998 | 0 |
| CAAGAT | 1 | 1 |
| CAAGCA | 0.9928 | 0 |
| CAAGCC | 1 | 1 |
| CAAGCG | 0.6351 | 0 |
| CAAGCT | 0.9919 | 0 |
| CAAGGA | 1 | 1 |
| CAAGGC | 0.6351 | 0 |
| CAAGGG | 0.1651 | 0 |
| CAAGGT | 0.2153 | 0 |
| CAAGTA | 1 | 1 |
| CAAGTC | 0.4621 | 0 |
| CAAGTG | 0.3364 | 0 |
| CAAGTT | 0.2449 | 0 |
| CAATAA | 0 | 0 |
| CAATAC | 0.2913 | 0 |
| CAATAG | 0.04996 | 0 |
| CAATAT | -0.112 | 0 |
| CAATCA | 1 | 1 |
| CAATCC | 0.4621 | 0 |
| CAATCG | 0.04996 | 0 |
| CAATCT | 0.1489 | 0 |
| CAATGA | 0.6351 | 0 |
| CAATGC | -0.4621 | 0 |
| CAATGG | -0.4219 | 0 |
| CAATGT | -0.6351 | 0 |
| CAATTA | 0.112 | 0 |
| CAATTC | 0.04996 | 0 |
| CAATTG | 0 | 0 |
| CAATTT | -0.3027 | 0 |
| CACAAA | 0.4136 | 0 |
| CACAAC | 1 | 1 |
| CACAAG | 0.978 | 0 |
| CACAAT | 0.2913 | 0 |
| CACACA | -1 | 1 |
| CACACC | 0 | 0 |
| CACACG | -0.1489 | 0 |
| CACACT | 0.1651 | 0 |
| CACAGA | 1 | 1 |
| CACAGC | 0.2913 | 0 |
| CACAGG | 0.6351 | 0 |
| CACAGT | 0.04996 | 0 |
| CACATA | -1 | 1 |
| CACATC | 0.3027 | 0 |
| CACATG | 0.1651 | 0 |
| CACATT | 0.112 | 0 |
| CACCAA | 1 | 1 |
| CACCAC | 0.4621 | 0 |
| CACCAG | 0.978 | 0 |
| CACCAT | 0.6351 | 0 |
| CACCCA | 0.664 | 0 |
| CACCCC | 0.1489 | 0 |
| CACCCG | 0.2913 | 0 |
| CACCCT | 1 | 1 |
| CACCGA | 1 | 1 |
| CACCGC | 0.04996 | 0 |
| CACCGG | 0.4621 | 0 |
| CACCGT | 0.2913 | 0 |
| CACCTA | 0.3027 | 0 |
| CACCTC | 0.4621 | 0 |
| CACCTG | 1 | 1 |
| CACCTT | 0.5459 | 0 |
| CACGAA | 1 | 1 |
| CACGAC | 0.978 | 0 |
| CACGAG | 0.7818 | 0 |
| CACGAT | 0.7818 | 0 |
| CACGCA | 0.1651 | 0 |
| CACGCC | 0.04996 | 0 |
| CACGCG | 0 | 0 |
| CACGCT | 0.04996 | 0 |
| CACGGA | 1 | 1 |
| CACGGC | 0.1489 | 0 |
| CACGGG | 0.04996 | 0 |
| CACGGT | 0.04996 | 0 |
| CACGTA | 0.1651 | 0 |
| CACGTC | 0 | 0 |
| CACGTG | 0.4621 | 0 |
| CACGTT | 0 | 0 |
| CACTAA | 0.1974 | 0 |
| CACTAC | 0.1974 | 0 |
| CACTAG | -0.2913 | 0 |
| CACTAT | -0.04996 | 0 |
| CACTCA | 0.112 | 0 |
| CACTCC | 0.04996 | 0 |
| CACTCG | 0 | 0 |
| CACTCT | 0.2913 | 0 |
| CACTGA | 1 | 1 |
| CACTGC | 0.4621 | 0 |
| CACTGG | 0.8854 | 0 |
| CACTGT | 0.1489 | 0 |
| CACTTA | 0 | 0 |
| CACTTC | 0.4621 | 0 |
| CACTTG | 0.2913 | 0 |
| CACTTT | -0.1489 | 0 |
| CAGAAA | 1 | 1 |
| CAGAAC | 1 | 1 |
| CAGAAG | 1 | 1 |
| CAGAAT | 1 | 1 |
| CAGACA | 0.8617 | 0 |
| CAGACC | 0.9999 | 0 |
| CAGACG | 0.9468 | 0 |
| CAGACT | 1 | 1 |
| CAGAGA | 1 | 1 |
| CAGAGC | 0.7818 | 0 |
| CAGAGG | 1 | 1 |
| CAGAGT | 0.8854 | 0 |
| CAGATA | 0.3364 | 0 |
| CAGATC | 1 | 1 |
| CAGATG | 0.7243 | 0 |
| CAGATT | 0.1974 | 0 |
| CAGCAA | 1 | 1 |
| CAGCAC | 0.9919 | 0 |
| CAGCAG | 1 | 1 |
| CAGCAT | 0.9919 | 0 |
| CAGCCA | 0.9468 | 0 |
| CAGCCC | 1 | 1 |
| CAGCCG | 0.664 | 0 |
| CAGCCT | 1 | 1 |
| CAGCGA | 0.8854 | 0 |
| CAGCGC | 0.2913 | 0 |
| CAGCGG | 0.6351 | 0 |
| CAGCGT | 1 | 1 |
| CAGCTA | 0.9468 | 0 |
| CAGCTC | 1 | 1 |
| CAGCTG | 1 | 1 |
| CAGCTT | 0.978 | 0 |
| CAGGAA | 1 | 1 |
| CAGGAC | 1 | 1 |
| CAGGAG | 1 | 1 |
| CAGGAT | 1 | 0 |
| CAGGCA | -0.112 | 0 |
| CAGGCC | 0.4621 | 0 |
| CAGGCG | 0.1489 | 0 |
| CAGGCT | 0.6351 | 0 |
| CAGGGA | 0.8854 | 0 |
| CAGGGC | 0.04996 | 0 |
| CAGGGG | -0.3364 | 0 |
| CAGGGT | -0.3027 | 0 |
| CAGGTA | -0.4355 | 0 |
| CAGGTC | 0.2913 | 0 |
| CAGGTG | 0.3027 | 0 |
| CAGGTT | -0.1974 | 0 |
| CAGTAA | 0.4621 | 0 |
| CAGTAC | 0.2913 | 0 |
| CAGTAG | 0.5459 | 0 |
| CAGTAT | -0.1974 | 0 |
| CAGTCA | 0.4621 | 0 |
| CAGTCC | 0.112 | 0 |
| CAGTCG | 0.112 | 0 |
| CAGTCT | 0.2913 | 0 |
| CAGTGA | 1 | 1 |
| CAGTGC | 0.2913 | 0 |
| CAGTGG | 0.7668 | 0 |
| CAGTGT | 0 | 0 |
| CAGTTA | -0.112 | 0 |
| CAGTTC | -0.1651 | 0 |
| CAGTTG | 0 | 0 |
| CAGTTT | -0.9468 | 0 |
| CATAAA | -1 | 1 |
| CATAAC | 0.1974 | 0 |
| CATAAG | 0.1974 | 0 |
| CATAAT | -0.3027 | 0 |
| CATACA | -1 | 1 |
| CATACC | -0.04996 | 0 |
| CATACG | 0 | 0 |
| CATACT | -0.2449 | 0 |
| CATAGA | 0 | 0 |
| CATAGC | 0 | 0 |
| CATAGG | -0.4219 | 0 |
| CATAGT | -0.2913 | 0 |
| CATATA | -1 | 1 |
| CATATC | -0.3364 | 0 |
| CATATG | -0.5503 | 0 |
| CATATT | -0.8854 | 0 |
| CATCAA | 0.9074 | 0 |
| CATCAC | 0.2913 | 0 |
| CATCAG | 1 | 1 |
| CATCAT | 0.2913 | 0 |
| CATCCA | 0.1651 | 0 |
| CATCCC | 0.04996 | 0 |
| CATCCG | 0.2913 | 0 |
| CATCCT | 0.6351 | 0 |
| CATCGA | 0.6351 | 0 |
| CATCGC | 0.04996 | 0 |
| CATCGG | 0.2913 | 0 |
| CATCGT | 0.04996 | 0 |
| CATCTA | 0.1651 | 0 |
| CATCTC | 0.6351 | 0 |
| CATCTG | 1 | 1 |
| CATCTT | 0.3364 | 0 |
| CATGAA | 0.8393 | 0 |
| CATGAC | 0.6351 | 0 |
| CATGAG | 0.9468 | 0 |
| CATGAT | 0.2449 | 0 |
| CATGCA | -1 | 1 |
| CATGCC | 0.112 | 0 |
| CATGCG | -0.04996 | 0 |
| CATGCT | -0.3027 | 0 |
| CATGGA | 0.8617 | 0 |
| CATGGC | 0.04996 | 0 |
| CATGGG | -0.8854 | 0 |
| CATGGT | -0.3027 | 0 |
| CATGTA | -1 | 1 |
| CATGTC | -0.7818 | 0 |
| CATGTG | 0 | 0 |
| CATGTT | -0.664 | 0 |
| CATTAA | -0.4219 | 0 |
| CATTAC | -0.04996 | 0 |
| CATTAG | -0.1974 | 0 |
| CATTAT | -0.6351 | 0 |
| CATTCA | 0.2913 | 0 |
| CATTCC | -0.04996 | 0 |
| CATTCG | -0.04996 | 0 |
| CATTCT | -0.4621 | 0 |
| CATTGA | 1 | 1 |
| CATTGC | 0.4621 | 0 |
| CATTGG | 0.7818 | 0 |
| CATTGT | 0.2153 | 0 |
| CATTTA | -0.8483 | 0 |
| CATTTC | -0.4621 | 0 |
| CATTTG | -0.3027 | 0 |
| CATTTT | -1 | 1 |
| CCAAAA | 0.6997 | 0 |
| CCAAAC | 0.4621 | 0 |
| CCAAAG | 0.4621 | 0 |
| CCAAAT | -0.04996 | 0 |
| CCAACA | 1 | 1 |
| CCAACC | 0.7818 | 0 |
| CCAACG | 0.7818 | 0 |
| CCAACT | 0.978 | 0 |
| CCAAGA | 1 | 1 |
| CCAAGC | 0.4621 | 0 |
| CCAAGG | 0.9468 | 0 |
| CCAAGT | 0.2913 | 0 |
| CCAATA | 0.112 | 0 |
| CCAATC | 0.1489 | 0 |
| CCAATG | 0.1974 | 0 |
| CCAATT | 0 | 0 |
| CCACAA | 0.1974 | 0 |
| CCACAC | 0 | 0 |
| CCACAG | 0.04996 | 0 |
| CCACAT | 0.112 | 0 |
| CCACCA | 0.5459 | 0 |
| CCACCC | 0.2913 | 0 |
| CCACCG | 0.1489 | 0 |
| CCACCT | 1 | 1 |
| CCACGA | 1 | 1 |
| CCACGC | 0.2913 | 0 |
| CCACGG | 0.4621 | 0 |
| CCACGT | 0.4621 | 0 |
| CCACTA | -1 | 1 |
| CCACTC | 0 | 0 |
| CCACTG | 0.5459 | 0 |
| CCACTT | 0.112 | 0 |
| CCAGAA | 1 | 1 |
| CCAGAC | 1 | 1 |
| CCAGAG | 0.9919 | 0 |
| CCAGAT | 0.9468 | 0 |
| CCAGCA | 1 | 1 |
| CCAGCC | 1 | 1 |
| CCAGCG | 0.7818 | 0 |
| CCAGCT | 1 | 1 |
| CCAGGA | 1 | 1 |
| CCAGGC | 0.6351 | 0 |
| CCAGGG | 0.1651 | 0 |
| CCAGGT | 0.1974 | 0 |
| CCAGTA | 0.664 | 0 |
| CCAGTC | 1 | 1 |
| CCAGTG | 1 | 1 |
| CCAGTT | 0.6351 | 0 |
| CCATAA | 0 | 0 |
| CCATAC | 0 | 0 |
| CCATAG | 0.04996 | 0 |
| CCATAT | -0.2913 | 0 |
| CCATCA | 0.9468 | 0 |
| CCATCC | 0.1489 | 0 |
| CCATCG | 0.1489 | 0 |
| CCATCT | 0.7818 | 0 |
| CCATGA | 0.7818 | 0 |
| CCATGC | 0 | 0 |
| CCATGG | 0.2913 | 0 |
| CCATGT | -0.1489 | 0 |
| CCATTA | -0.112 | 0 |
| CCATTC | 0.1974 | 0 |
| CCATTG | 1 | 1 |
| CCATTT | -0.1974 | 0 |
| CCCAAA | 0 | 0 |
| CCCAAC | 0.4621 | 0 |
| CCCAAG | 0.6351 | 0 |
| CCCAAT | 0 | 0 |
| CCCACA | 0 | 0 |
| CCCACC | 0.04996 | 0 |
| CCCACG | 0.4621 | 0 |
| CCCACT | 0 | 0 |
| CCCAGA | 0.9919 | 0 |
| CCCAGC | 0.8854 | 0 |
| CCCAGG | 1 | 1 |
| CCCAGT | 0.6351 | 0 |
| CCCATA | -0.04996 | 0 |
| CCCATC | 0.04996 | 0 |
| CCCATG | 0.1489 | 0 |
| CCCATT | 0.04996 | 0 |
| CCCCAA | 0.05411 | 0 |
| CCCCAC | 0.04996 | 0 |
| CCCCAG | 0.1489 | 0 |
| CCCCAT | 0.04996 | 0 |
| CCCCCA | 0 | 0 |
| CCCCCC | 0 | 0 |
| CCCCCG | 0 | 0 |
| CCCCCT | 0.314 | 0 |
| CCCCGA | 0.6351 | 0 |
| CCCCGC | 0.04996 | 0 |
| CCCCGG | 0.671 | 0 |
| CCCCGT | 0 | 0 |
| CCCCTA | -0.04996 | 0 |
| CCCCTC | 0 | 0 |
| CCCCTG | 0.9919 | 0 |
| CCCCTT | 0.1611 | 0 |
| CCCGAA | 0.9468 | 0 |
| CCCGAC | 0.6351 | 0 |
| CCCGAG | 0.4621 | 0 |
| CCCGAT | 0.04996 | 0 |
| CCCGCA | 0.2913 | 0 |
| CCCGCC | 0.1489 | 0 |
| CCCGCG | 0 | 0 |
| CCCGCT | 0.2913 | 0 |
| CCCGGA | 1 | 1 |
| CCCGGC | 0.8854 | 0 |
| CCCGGG | 0.6997 | 0 |
| CCCGGT | 0.2913 | 0 |
| CCCGTA | 0.04996 | 0 |
| CCCGTC | 0.04996 | 0 |
| CCCGTG | 0.4621 | 0 |
| CCCGTT | -0.112 | 0 |
| CCCTAA | 0.04996 | 0 |
| CCCTAC | 0.04996 | 0 |
| CCCTAG | 0.1489 | 0 |
| CCCTAT | 0 | 0 |
| CCCTCA | 0.2913 | 0 |
| CCCTCC | 0.1489 | 0 |
| CCCTCG | 0.4621 | 0 |
| CCCTCT | 0.1489 | 0 |
| CCCTGA | 1 | 1 |
| CCCTGC | 1 | 1 |
| CCCTGG | 1 | 1 |
| CCCTGT | 0.9919 | 0 |
| CCCTTA | 0.2913 | 0 |
| CCCTTC | 0.3027 | 0 |
| CCCTTG | 1 | 1 |
| CCCTTT | 0.1611 | 0 |
| CCGAAA | 0.9468 | 0 |
| CCGAAC | 0.8854 | 0 |
| CCGAAG | 1 | 1 |
| CCGAAT | 0.8854 | 0 |
| CCGACA | 0.7818 | 0 |
| CCGACC | 0.7818 | 0 |
| CCGACG | 0.8854 | 0 |
| CCGACT | 1 | 1 |
| CCGAGA | 0.7818 | 0 |
| CCGAGC | 0.4621 | 0 |
| CCGAGG | 1 | 1 |
| CCGAGT | 0.1489 | 0 |
| CCGATA | 0 | 0 |
| CCGATC | 0.3027 | 0 |
| CCGATG | 0.1489 | 0 |
| CCGATT | 0.2913 | 0 |
| CCGCAA | 0.2913 | 0 |
| CCGCAC | 0 | 0 |
| CCGCAG | 0.4621 | 0 |
| CCGCAT | 0.04996 | 0 |
| CCGCCA | 0.04996 | 0 |
| CCGCCC | 0.1489 | 0 |
| CCGCCG | 0 | 0 |
| CCGCCT | 0.7818 | 0 |
| CCGCGA | 0.2913 | 0 |
| CCGCGC | 0 | 0 |
| CCGCGG | 0.04996 | 0 |
| CCGCGT | 0.04996 | 0 |
| CCGCTA | -0.04996 | 0 |
| CCGCTC | 0.112 | 0 |
| CCGCTG | 0.6351 | 0 |
| CCGCTT | 0 | 0 |
| CCGGAA | 1 | 1 |
| CCGGAC | 0.9919 | 0 |
| CCGGAG | 1 | 1 |
| CCGGAT | 1 | 1 |
| CCGGCA | 0.7818 | 0 |
| CCGGCC | 0.4621 | 0 |
| CCGGCG | 0.4621 | 0 |
| CCGGCT | 1 | 1 |
| CCGGGA | 1 | 1 |
| CCGGGC | 0.1489 | 0 |
| CCGGGG | 0.2644 | 0 |
| CCGGGT | 0.4621 | 0 |
| CCGGTA | 0.1489 | 0 |
| CCGGTC | 0 | 0 |
| CCGGTG | 0.6351 | 0 |
| CCGGTT | 0.2913 | 0 |
| CCGTAA | 0.04996 | 0 |
| CCGTAC | -0.04996 | 0 |
| CCGTAG | 0.1974 | 0 |
| CCGTAT | 0.1489 | 0 |
| CCGTCA | 0.1489 | 0 |
| CCGTCC | -0.04996 | 0 |
| CCGTCG | 0.2913 | 0 |
| CCGTCT | 0.1489 | 0 |
| CCGTGA | 0.9468 | 0 |
| CCGTGC | 0.1974 | 0 |
| CCGTGG | 0.9468 | 0 |
| CCGTGT | 0.04996 | 0 |
| CCGTTA | -0.1489 | 0 |
| CCGTTC | -1 | 1 |
| CCGTTG | 0 | 0 |
| CCGTTT | -0.2913 | 0 |
| CCTAAA | 0 | 0 |
| CCTAAC | 0.04996 | 0 |
| CCTAAG | 0.1489 | 0 |
| CCTAAT | 0 | 0 |
| CCTACA | 0.4219 | 0 |
| CCTACC | 0.04996 | 0 |
| CCTACG | 0 | 0 |
| CCTACT | 0.2913 | 0 |
| CCTAGA | 0.4621 | 0 |
| CCTAGC | 0 | 0 |
| CCTAGG | -0.4621 | 0 |
| CCTAGT | -0.04996 | 0 |
| CCTATA | -0.04996 | 0 |
| CCTATC | 0 | 0 |
| CCTATG | 0.1974 | 0 |
| CCTATT | -0.112 | 0 |
| CCTCAA | 0.6351 | 0 |
| CCTCAC | 0.2913 | 0 |
| CCTCAG | 0.6351 | 0 |
| CCTCAT | 0.1489 | 0 |
| CCTCCA | 0.7818 | 0 |
| CCTCCC | 0.1489 | 0 |
| CCTCCG | 0.1489 | 0 |
| CCTCCT | 1 | 1 |
| CCTCGA | 1 | 1 |
| CCTCGC | 0.2913 | 0 |
| CCTCGG | 0.4219 | 0 |
| CCTCGT | 0.2913 | 0 |
| CCTCTA | 0.1651 | 0 |
| CCTCTC | 0.1489 | 0 |
| CCTCTG | 1 | 1 |
| CCTCTT | 0.3027 | 0 |
| CCTGAA | 1 | 1 |
| CCTGAC | 1 | 1 |
| CCTGAG | 1 | 1 |
| CCTGAT | 0.9973 | 0 |
| CCTGCA | 1 | 1 |
| CCTGCC | 1 | 1 |
| CCTGCG | 0.7668 | 0 |
| CCTGCT | 1 | 1 |
| CCTGGA | 1 | 1 |
| CCTGGC | 0.664 | 0 |
| CCTGGG | -1 | 1 |
| CCTGGT | 0.5459 | 0 |
| CCTGTA | 0.7243 | 0 |
| CCTGTC | 0.978 | 0 |
| CCTGTG | 1 | 1 |
| CCTGTT | 1 | 1 |
| CCTTAA | 0.2913 | 0 |
| CCTTAC | 0.04996 | 0 |
| CCTTAG | -0.1651 | 0 |
| CCTTAT | -0.2913 | 0 |
| CCTTCA | 1 | 1 |
| CCTTCC | 0.112 | 0 |
| CCTTCG | 0.3027 | 0 |
| CCTTCT | 0.1651 | 0 |
| CCTTGA | 1 | 1 |
| CCTTGC | 0.7818 | 0 |
| CCTTGG | 1 | 1 |
| CCTTGT | 0.4219 | 0 |
| CCTTTA | 0 | 0 |
| CCTTTC | -0.1489 | 0 |
| CCTTTG | 0.6351 | 0 |
| CCTTTT | -0.2133 | 0 |
| CGAAAA | 1 | 1 |
| CGAAAC | 0.9998 | 0 |
| CGAAAG | 0.9998 | 0 |
| CGAAAT | 0.3027 | 0 |
| CGAACA | 1 | 1 |
| CGAACC | 0.978 | 0 |
| CGAACG | 0.7818 | 0 |
| CGAACT | 0.8854 | 0 |
| CGAAGA | 1 | 1 |
| CGAAGC | 0.9973 | 0 |
| CGAAGG | 1 | 1 |
| CGAAGT | 0.9973 | 0 |
| CGAATA | 0.6351 | 0 |
| CGAATC | 0.7818 | 0 |
| CGAATG | 1 | 1 |
| CGAATT | 0.1489 | 0 |
| CGACAA | 0.9468 | 0 |
| CGACAC | 1 | 1 |
| CGACAG | 0.6351 | 0 |
| CGACAT | 0.7818 | 0 |
| CGACCA | 0.9992 | 0 |
| CGACCC | 0.7818 | 0 |
| CGACCG | 0.4621 | 0 |
| CGACCT | 0.8854 | 0 |
| CGACGA | 1 | 1 |
| CGACGC | 0.2913 | 0 |
| CGACGG | 0.2913 | 0 |
| CGACGT | 0.4621 | 0 |
| CGACTA | 0.3027 | 0 |
| CGACTC | 0.8854 | 0 |
| CGACTG | 1 | 1 |
| CGACTT | 0.6351 | 0 |
| CGAGAA | 0.9998 | 0 |
| CGAGAC | 0.9468 | 0 |
| CGAGAG | 0.8854 | 0 |
| CGAGAT | 0.8854 | 0 |
| CGAGCA | 1 | 1 |
| CGAGCC | 0.6351 | 0 |
| CGAGCG | 0.1489 | 0 |
| CGAGCT | 0.8854 | 0 |
| CGAGGA | 1 | 1 |
| CGAGGC | 0.6351 | 0 |
| CGAGGG | 0 | 0 |
| CGAGGT | 0.3027 | 0 |
| CGAGTA | 0.4621 | 0 |
| CGAGTC | 0.2913 | 0 |
| CGAGTG | 0.4621 | 0 |
| CGAGTT | 0 | 0 |
| CGATAA | 0.1489 | 0 |
| CGATAC | 0.1489 | 0 |
| CGATAG | -0.04996 | 0 |
| CGATAT | 0.1489 | 0 |
| CGATCA | 1 | 1 |
| CGATCC | 0.5459 | 0 |
| CGATCG | 0.112 | 0 |
| CGATCT | 0.3027 | 0 |
| CGATGA | 0.978 | 0 |
| CGATGC | 0.2913 | 0 |
| CGATGG | 0.2153 | 0 |
| CGATGT | 0.04996 | 0 |
| CGATTA | 0.1651 | 0 |
| CGATTC | 1 | 1 |
| CGATTG | 0.6351 | 0 |
| CGATTT | 0.112 | 0 |
| CGCAAA | 0.4621 | 0 |
| CGCAAC | 0.4621 | 0 |
| CGCAAG | 0.7818 | 0 |
| CGCAAT | 0.04996 | 0 |
| CGCACA | 0 | 0 |
| CGCACC | 0.04996 | 0 |
| CGCACG | 0 | 0 |
| CGCACT | 0.1489 | 0 |
| CGCAGA | 0.8854 | 0 |
| CGCAGC | 0.2913 | 0 |
| CGCAGG | 0.5459 | 0 |
| CGCAGT | 0.04996 | 0 |
| CGCATA | -0.1489 | 0 |
| CGCATC | 0.1489 | 0 |
| CGCATG | 0.1974 | 0 |
| CGCATT | 0.1489 | 0 |
| CGCCAA | 0.04996 | 0 |
| CGCCAC | 0.1489 | 0 |
| CGCCAG | 0.04996 | 0 |
| CGCCAT | 0.04996 | 0 |
| CGCCCA | 0 | 0 |
| CGCCCC | 0 | 0 |
| CGCCCG | 0.04996 | 0 |
| CGCCCT | 0.4621 | 0 |
| CGCCGA | 0.2913 | 0 |
| CGCCGC | 0.04996 | 0 |
| CGCCGG | 0.112 | 0 |
| CGCCGT | 0.04996 | 0 |
| CGCCTA | 0.04996 | 0 |
| CGCCTC | 0.1489 | 0 |
| CGCCTG | 1 | 1 |
| CGCCTT | 0.2913 | 0 |
| CGCGAA | 0.4621 | 0 |
| CGCGAC | 0.04996 | 0 |
| CGCGAG | 0.1489 | 0 |
| CGCGAT | 0 | 0 |
| CGCGCA | 0.04996 | 0 |
| CGCGCC | 0 | 0 |
| CGCGCG | 0 | 0 |
| CGCGCT | 0 | 0 |
| CGCGGA | 0.7818 | 0 |
| CGCGGC | 0.04996 | 0 |
| CGCGGG | 0 | 0 |
| CGCGGT | 0 | 0 |
| CGCGTA | 0 | 0 |
| CGCGTC | 0 | 0 |
| CGCGTG | 0.04996 | 0 |
| CGCGTT | 0 | 0 |
| CGCTAA | 0 | 0 |
| CGCTAC | 0 | 0 |
| CGCTAG | 0.04996 | 0 |
| CGCTAT | 0 | 0 |
| CGCTCA | 0 | 0 |
| CGCTCC | -0.04996 | 0 |
| CGCTCG | 0 | 0 |
| CGCTCT | 0.1974 | 0 |
| CGCTGA | 0.978 | 0 |
| CGCTGC | 0.4621 | 0 |
| CGCTGG | 1 | 1 |
| CGCTGT | 0.7818 | 0 |
| CGCTTA | -0.1489 | 0 |
| CGCTTC | 0.04996 | 0 |
| CGCTTG | 0.2913 | 0 |
| CGCTTT | -0.04996 | 0 |
| CGGAAA | 0.9998 | 0 |
| CGGAAC | 1 | 1 |
| CGGAAG | 1 | 1 |
| CGGAAT | 0.9468 | 0 |
| CGGACA | 0.8854 | 0 |
| CGGACC | 1 | 1 |
| CGGACG | 0.8854 | 0 |
| CGGACT | 0.8854 | 0 |
| CGGAGA | 1 | 1 |
| CGGAGC | 0.9919 | 0 |
| CGGAGG | 1 | 1 |
| CGGAGT | 0.7818 | 0 |
| CGGATA | 0.1489 | 0 |
| CGGATC | 0.9919 | 0 |
| CGGATG | 0.537 | 0 |
| CGGATT | 0.2913 | 0 |
| CGGCAA | 0.04996 | 0 |
| CGGCAC | 0.1489 | 0 |
| CGGCAG | 0.6351 | 0 |
| CGGCAT | -0.1489 | 0 |
| CGGCCA | 0.04996 | 0 |
| CGGCCC | 0.2913 | 0 |
| CGGCCG | 0.1489 | 0 |
| CGGCCT | 0.04996 | 0 |
| CGGCGA | 0.1489 | 0 |
| CGGCGC | 0.04996 | 0 |
| CGGCGG | 0.3027 | 0 |
| CGGCGT | 0.04996 | 0 |
| CGGCTA | 0.2913 | 0 |
| CGGCTC | 0.7818 | 0 |
| CGGCTG | 1 | 1 |
| CGGCTT | 0.1651 | 0 |
| CGGGAA | 0.6351 | 0 |
| CGGGAC | 0.4621 | 0 |
| CGGGAG | 0.4136 | 0 |
| CGGGAT | 0.1651 | 0 |
| CGGGCA | 0.04996 | 0 |
| CGGGCC | 0.04996 | 0 |
| CGGGCG | 0 | 0 |
| CGGGCT | 0.1489 | 0 |
| CGGGGA | -0.2636 | 0 |
| CGGGGC | -0.04996 | 0 |
| CGGGGG | -1 | 1 |
| CGGGGT | -0.8854 | 0 |
| CGGGTA | -0.4621 | 0 |
| CGGGTC | 0 | 0 |
| CGGGTG | -0.4355 | 0 |
| CGGGTT | -0.6351 | 0 |
| CGGTAA | -0.2913 | 0 |
| CGGTAC | 0.04996 | 0 |
| CGGTAG | -0.2913 | 0 |
| CGGTAT | -0.2913 | 0 |
| CGGTCA | 0 | 0 |
| CGGTCC | 0.112 | 0 |
| CGGTCG | 0.112 | 0 |
| CGGTCT | -0.04996 | 0 |
| CGGTGA | 0.7818 | 0 |
| CGGTGC | 0.1489 | 0 |
| CGGTGG | 1 | 1 |
| CGGTGT | -0.112 | 0 |
| CGGTTA | -0.6351 | 0 |
| CGGTTC | 0 | 0 |
| CGGTTG | 0 | 0 |
| CGGTTT | -0.8854 | 0 |
| CGTAAA | -0.1651 | 0 |
| CGTAAC | 0.04996 | 0 |
| CGTAAG | -0.2153 | 0 |
| CGTAAT | -0.1489 | 0 |
| CGTACA | -0.112 | 0 |
| CGTACC | 0 | 0 |
| CGTACG | -0.112 | 0 |
| CGTACT | -0.04996 | 0 |
| CGTAGA | 0.5459 | 0 |
| CGTAGC | 0 | 0 |
| CGTAGG | -1 | 1 |
| CGTAGT | -0.6351 | 0 |
| CGTATA | -0.5459 | 0 |
| CGTATC | 0.04996 | 0 |
| CGTATG | 1 | 1 |
| CGTATT | -0.112 | 0 |
| CGTCAA | 0.7818 | 0 |
| CGTCAC | 0.4621 | 0 |
| CGTCAG | 0.4621 | 0 |
| CGTCAT | -0.112 | 0 |
| CGTCCA | 0 | 0 |
| CGTCCC | 0 | 0 |
| CGTCCG | -0.2913 | 0 |
| CGTCCT | 0.1974 | 0 |
| CGTCGA | 0.9468 | 0 |
| CGTCGC | 1 | 1 |
| CGTCGG | 0.112 | 0 |
| CGTCGT | 0 | 0 |
| CGTCTA | 0 | 0 |
| CGTCTC | 0.04996 | 0 |
| CGTCTG | 0.7818 | 0 |
| CGTCTT | -0.04996 | 0 |
| CGTGAA | 1 | 1 |
| CGTGAC | 0.7818 | 0 |
| CGTGAG | 1 | 1 |
| CGTGAT | 0.2913 | 0 |
| CGTGCA | 0.4355 | 0 |
| CGTGCC | 0 | 0 |
| CGTGCG | 0.1974 | 0 |
| CGTGCT | 0.1974 | 0 |
| CGTGGA | 1 | 1 |
| CGTGGC | 0.4219 | 0 |
| CGTGGG | -0.4621 | 0 |
| CGTGGT | 0.112 | 0 |
| CGTGTA | -0.2449 | 0 |
| CGTGTC | 0.04996 | 0 |
| CGTGTG | 0.1974 | 0 |
| CGTGTT | -0.3027 | 0 |
| CGTTAA | -0.112 | 0 |
| CGTTAC | -0.04996 | 0 |
| CGTTAG | -0.5459 | 0 |
| CGTTAT | -0.7818 | 0 |
| CGTTCA | -1 | 1 |
| CGTTCC | -1 | 1 |
| CGTTCG | -1 | 1 |
| CGTTCT | -1 | 1 |
| CGTTGA | 0.1974 | 0 |
| CGTTGC | 0 | 0 |
| CGTTGG | 0 | 0 |
| CGTTGT | -0.2913 | 0 |
| CGTTTA | -0.8854 | 0 |
| CGTTTC | -0.4219 | 0 |
| CGTTTG | -0.5459 | 0 |
| CGTTTT | -0.9468 | 0 |
| CTAAAA | -0.3364 | 0 |
| CTAAAC | 0.1489 | 0 |
| CTAAAG | 0.4621 | 0 |
| CTAAAT | -0.3027 | 0 |
| CTAACA | 0.1974 | 0 |
| CTAACC | 0.04996 | 0 |
| CTAACG | 0.04996 | 0 |
| CTAACT | -0.112 | 0 |
| CTAAGA | 0.2913 | 0 |
| CTAAGC | 0.04996 | 0 |
| CTAAGG | -0.2449 | 0 |
| CTAAGT | -0.6351 | 0 |
| CTAATA | -0.1489 | 0 |
| CTAATC | 0.04996 | 0 |
| CTAATG | -0.8483 | 0 |
| CTAATT | -0.6351 | 0 |
| CTACAA | 0.6351 | 0 |
| CTACAC | 0.6351 | 0 |
| CTACAG | 0.6351 | 0 |
| CTACAT | 1 | 1 |
| CTACCA | 0.1489 | 0 |
| CTACCC | 0.04996 | 0 |
| CTACCG | 0 | 0 |
| CTACCT | 0.2913 | 0 |
| CTACGA | 0.6351 | 0 |
| CTACGC | 0 | 0 |
| CTACGG | -0.04996 | 0 |
| CTACGT | 0 | 0 |
| CTACTA | -0.4621 | 0 |
| CTACTC | 0.04996 | 0 |
| CTACTG | 0.2913 | 0 |
| CTACTT | -0.1651 | 0 |
| CTAGAA | 0.8854 | 0 |
| CTAGAC | 0.4621 | 0 |
| CTAGAG | 0.1974 | 0 |
| CTAGAT | 0 | 0 |
| CTAGCA | 0.1489 | 0 |
| CTAGCC | 0.1489 | 0 |
| CTAGCG | 0 | 0 |
| CTAGCT | 0.112 | 0 |
| CTAGGA | 0.2636 | 0 |
| CTAGGC | -0.4219 | 0 |
| CTAGGG | -1 | 1 |
| CTAGGT | -1 | 1 |
| CTAGTA | -0.1974 | 0 |
| CTAGTC | 0.04996 | 0 |
| CTAGTG | -0.2449 | 0 |
| CTAGTT | -0.8854 | 0 |
| CTATAA | -0.4621 | 0 |
| CTATAC | -0.04996 | 0 |
| CTATAG | -0.3027 | 0 |
| CTATAT | -0.8483 | 0 |
| CTATCA | 0.1489 | 0 |
| CTATCC | 0 | 0 |
| CTATCG | 0.04996 | 0 |
| CTATCT | -0.4621 | 0 |
| CTATGA | 0.04996 | 0 |
| CTATGC | -0.2449 | 0 |
| CTATGG | -0.2449 | 0 |
| CTATGT | -0.9468 | 0 |
| CTATTA | -0.7818 | 0 |
| CTATTC | -0.1974 | 0 |
| CTATTG | -0.3027 | 0 |
| CTATTT | -1 | 1 |
| CTCAAA | 0.4621 | 0 |
| CTCAAC | 0.8854 | 0 |
| CTCAAG | 1 | 1 |
| CTCAAT | 0.2913 | 0 |
| CTCACA | -0.04996 | 0 |
| CTCACC | 0.04996 | 0 |
| CTCACG | 0.1489 | 0 |
| CTCACT | 0 | 0 |
| CTCAGA | 0.4621 | 0 |
| CTCAGC | 0.9468 | 0 |
| CTCAGG | 0.3027 | 0 |
| CTCAGT | -0.04996 | 0 |
| CTCATA | -0.2913 | 0 |
| CTCATC | 0.1489 | 0 |
| CTCATG | -0.2449 | 0 |
| CTCATT | -0.2913 | 0 |
| CTCCAA | 0.8854 | 0 |
| CTCCAC | 1 | 1 |
| CTCCAG | 0.978 | 0 |
| CTCCAT | 1 | 1 |
| CTCCCA | 0.04996 | 0 |
| CTCCCC | 0.1489 | 0 |
| CTCCCG | 0.1489 | 0 |
| CTCCCT | 0.1489 | 0 |
| CTCCGA | 0.1489 | 0 |
| CTCCGC | 0.2913 | 0 |
| CTCCGG | 0.4621 | 0 |
| CTCCGT | -0.1974 | 0 |
| CTCCTA | 0.1489 | 0 |
| CTCCTC | 0.8854 | 0 |
| CTCCTG | 1 | 1 |
| CTCCTT | 0.1974 | 0 |
| CTCGAA | 1 | 1 |
| CTCGAC | 0.9468 | 0 |
| CTCGAG | 1 | 1 |
| CTCGAT | 0.6351 | 0 |
| CTCGCA | 0.04996 | 0 |
| CTCGCC | 0.04996 | 0 |
| CTCGCG | 0.1489 | 0 |
| CTCGCT | 0.04996 | 0 |
| CTCGGA | 0.6351 | 0 |
| CTCGGC | 1 | 1 |
| CTCGGG | 0 | 0 |
| CTCGGT | 0.112 | 0 |
| CTCGTA | -0.112 | 0 |
| CTCGTC | 0.2913 | 0 |
| CTCGTG | 0.2913 | 0 |
| CTCGTT | -0.2913 | 0 |
| CTCTAA | 0 | 0 |
| CTCTAC | 0.112 | 0 |
| CTCTAG | 0 | 0 |
| CTCTAT | -0.3027 | 0 |
| CTCTCA | 0 | 0 |
| CTCTCC | 0.1489 | 0 |
| CTCTCG | 0.04996 | 0 |
| CTCTCT | -0.4777 | 0 |
| CTCTGA | 0.978 | 0 |
| CTCTGC | 1 | 1 |
| CTCTGG | 0.978 | 0 |
| CTCTGT | 0.2449 | 0 |
| CTCTTA | -0.2913 | 0 |
| CTCTTC | 0.1974 | 0 |
| CTCTTG | 0.112 | 0 |
| CTCTTT | -0.8854 | 0 |
| CTGAAA | 1 | 1 |
| CTGAAC | 1 | 1 |
| CTGAAG | 1 | 1 |
| CTGAAT | 0.978 | 0 |
| CTGACA | 0.9468 | 0 |
| CTGACC | 1 | 1 |
| CTGACG | 1 | 1 |
| CTGACT | 0.9919 | 0 |
| CTGAGA | 0.9919 | 0 |
| CTGAGC | 1 | 1 |
| CTGAGG | 1 | 1 |
| CTGAGT | 0.4219 | 0 |
| CTGATA | 0.112 | 0 |
| CTGATC | 1 | 1 |
| CTGATG | 0.7668 | 0 |
| CTGATT | 0 | 0 |
| CTGCAA | 0.978 | 0 |
| CTGCAC | 0.978 | 0 |
| CTGCAG | 1 | 1 |
| CTGCAT | 0.4355 | 0 |
| CTGCCA | 1 | 1 |
| CTGCCC | 1 | 1 |
| CTGCCG | 0.9468 | 0 |
| CTGCCT | 0.6351 | 0 |
| CTGCGA | 0.4621 | 0 |
| CTGCGC | 0.4621 | 0 |
| CTGCGG | 0.7818 | 0 |
| CTGCGT | 0.04996 | 0 |
| CTGCTA | 0.6351 | 0 |
| CTGCTC | 1 | 1 |
| CTGCTG | 1 | 1 |
| CTGCTT | 0.2449 | 0 |
| CTGGAA | 1 | 1 |
| CTGGAC | 1 | 1 |
| CTGGAG | 1 | 1 |
| CTGGAT | 1 | 1 |
| CTGGCA | 0.4219 | 0 |
| CTGGCC | 0.8854 | 0 |
| CTGGCG | 1 | 1 |
| CTGGCT | 1 | 1 |
| CTGGGA | 0.1651 | 0 |
| CTGGGC | 0.3027 | 0 |
| CTGGGG | -0.5964 | 0 |
| CTGGGT | -0.3364 | 0 |
| CTGGTA | -0.1651 | 0 |
| CTGGTC | 0.7818 | 0 |
| CTGGTG | 1 | 1 |
| CTGGTT | -0.2913 | 0 |
| CTGTAA | 0 | 0 |
| CTGTAC | 0.4621 | 0 |
| CTGTAG | 1 | 1 |
| CTGTAT | -0.537 | 0 |
| CTGTCA | 0 | 0 |
| CTGTCC | 0.6351 | 0 |
| CTGTCG | 1 | 1 |
| CTGTCT | -0.2449 | 0 |
| CTGTGA | 0.6351 | 0 |
| CTGTGC | 1 | 1 |
| CTGTGG | 1 | 1 |
| CTGTGT | 0 | 0 |
| CTGTTA | -0.6351 | 0 |
| CTGTTC | -0.3103 | 0 |
| CTGTTG | 0.4621 | 0 |
| CTGTTT | -1 | 1 |
| CTTAAA | -0.2913 | 0 |
| CTTAAC | -0.1651 | 0 |
| CTTAAG | 0.2449 | 0 |
| CTTAAT | -0.8854 | 0 |
| CTTACA | -0.1489 | 0 |
| CTTACC | 0 | 0 |
| CTTACG | -0.112 | 0 |
| CTTACT | -0.4621 | 0 |
| CTTAGA | -0.2449 | 0 |
| CTTAGC | -0.1974 | 0 |
| CTTAGG | -1 | 1 |
| CTTAGT | -1 | 1 |
| CTTATA | -0.8854 | 0 |
| CTTATC | -0.112 | 0 |
| CTTATG | -1 | 1 |
| CTTATT | -0.9919 | 0 |
| CTTCAA | 1 | 1 |
| CTTCAC | 1 | 1 |
| CTTCAG | 1 | 1 |
| CTTCAT | 0.4219 | 0 |
| CTTCCA | 0.2913 | 0 |
| CTTCCC | 0.1489 | 0 |
| CTTCCG | -0.112 | 0 |
| CTTCCT | -0.3364 | 0 |
| CTTCGA | 1 | 1 |
| CTTCGC | 0.4621 | 0 |
| CTTCGG | 0.1651 | 0 |
| CTTCGT | -0.3364 | 0 |
| CTTCTA | -0.3364 | 0 |
| CTTCTC | 0 | 0 |
| CTTCTG | 0.2913 | 0 |
| CTTCTT | -0.4621 | 0 |
| CTTGAA | 1 | 1 |
| CTTGAC | 0.978 | 0 |
| CTTGAG | 1 | 1 |
| CTTGAT | 0.4219 | 0 |
| CTTGCA | 0.1651 | 0 |
| CTTGCC | 0.04996 | 0 |
| CTTGCG | 0.1974 | 0 |
| CTTGCT | 0 | 0 |
| CTTGGA | 1 | 1 |
| CTTGGC | 0.1974 | 0 |
| CTTGGG | -1 | 1 |
| CTTGGT | -0.1651 | 0 |
| CTTGTA | -1 | 1 |
| CTTGTC | -0.04996 | 0 |
| CTTGTG | -0.2153 | 0 |
| CTTGTT | -0.8483 | 0 |
| CTTTAA | -0.6351 | 0 |
| CTTTAC | -0.5459 | 0 |
| CTTTAG | -0.3746 | 0 |
| CTTTAT | -1 | 1 |
| CTTTCA | -0.3027 | 0 |
| CTTTCC | -0.4621 | 0 |
| CTTTCG | -0.1974 | 0 |
| CTTTCT | -1 | 1 |
| CTTTGA | 0.4621 | 0 |
| CTTTGC | 0.112 | 0 |
| CTTTGG | -0.2913 | 0 |
| CTTTGT | -0.978 | 0 |
| CTTTTA | -0.9468 | 0 |
| CTTTTC | -0.6351 | 0 |
| CTTTTG | -0.8854 | 0 |
| CTTTTT | -1 | 1 |
| | GAAAAA | 1 | 1 | | --- | --- | --- | |  |  |
| GAAAAC | 1 | 1 |
| GAAAAG | 1 | 1 |
| GAAAAT | 1 | 1 |
| GAAACA | 1 | 1 |
| GAAACC | 1 | 1 |
| GAAACG | 1 | 1 |
| GAAACT | 1 | 1 |
| GAAAGA | 1 | 1 |
| GAAAGC | 1 | 1 |
| GAAAGG | 0.7779 | 0 |
| GAAAGT | 1 | 1 |
| GAAATA | 0.3103 | 0 |
| GAAATC | 1 | 1 |
| GAAATG | -1 | 1 |
| GAAATT | 0.664 | 0 |
| GAACAA | 1 | 1 |
| GAACAC | 1 | 1 |
| GAACAG | 1 | 1 |
| GAACAT | 1 | 1 |
| GAACCA | 1 | 1 |
| GAACCC | 1 | 1 |
| GAACCG | 0.9992 | 0 |
| GAACCT | 1 | 1 |
| GAACGA | 1 | 1 |
| GAACGC | 0.6351 | 0 |
| GAACGG | 0.5459 | 0 |
| GAACGT | 0.8854 | 0 |
| GAACTA | 0.9074 | 0 |
| GAACTC | 0.9919 | 0 |
| GAACTG | 1 | 1 |
| GAACTT | 1 | 1 |
| GAAGAA | 1 | 1 |
| GAAGAC | 1 | 1 |
| GAAGAG | 1 | 1 |
| GAAGAT | 1 | 1 |
| GAAGCA | 1 | 1 |
| GAAGCC | 1 | 1 |
| GAAGCG | 1 | 1 |
| GAAGCT | 1 | 1 |
| GAAGGA | 1 | 1 |
| GAAGGC | 0.8854 | 0 |
| GAAGGG | 0.3799 | 0 |
| GAAGGT | 0.6351 | 0 |
| GAAGTA | 1 | 1 |
| GAAGTC | 0.9919 | 0 |
| GAAGTG | 1 | 1 |
| GAAGTT | 1 | 1 |
| GAATAA | 0.2449 | 0 |
| GAATAC | 0.4621 | 0 |
| GAATAG | 0 | 0 |
| GAATAT | 0 | 0 |
| GAATCA | 1 | 1 |
| GAATCC | 0.9919 | 0 |
| GAATCG | 0.4219 | 0 |
| GAATCT | 0.9468 | 0 |
| GAATGA | 0.5717 | 0 |
| GAATGC | -0.2913 | 0 |
| GAATGG | -1 | 1 |
| GAATGT | -0.4621 | 0 |
| GAATTA | 0.112 | 0 |
| GAATTC | 0.2913 | 0 |
| GAATTG | 0.1651 | 0 |
| GAATTT | -0.1651 | 0 |
| GACAAA | 1 | 1 |
| GACAAC | 0.9999 | 0 |
| GACAAG | 1 | 1 |
| GACAAT | 1 | 1 |
| GACACA | 0.537 | 0 |
| GACACC | 0.9468 | 0 |
| GACACG | 0.7818 | 0 |
| GACACT | 0.8854 | 0 |
| GACAGA | 0.9973 | 0 |
| GACAGC | 0.6351 | 0 |
| GACAGG | 1 | 1 |
| GACAGT | 0.7818 | 0 |
| GACATA | 0.2913 | 0 |
| GACATC | 1 | 1 |
| GACATG | 0.7818 | 0 |
| GACATT | 1 | 1 |
| GACCAA | 1 | 1 |
| GACCAC | 0.9919 | 0 |
| GACCAG | 1 | 1 |
| GACCAT | 1 | 1 |
| GACCCA | 1 | 1 |
| GACCCC | 0.7818 | 0 |
| GACCCG | 0.9468 | 0 |
| GACCCT | 1 | 1 |
| GACCGA | 0.9919 | 0 |
| GACCGC | 0.1489 | 0 |
| GACCGG | 1 | 1 |
| GACCGT | 0.4219 | 0 |
| GACCTA | 0.9468 | 0 |
| GACCTC | 0.9468 | 0 |
| GACCTG | 1 | 1 |
| GACCTT | 1 | 1 |
| GACGAA | 1 | 1 |
| GACGAC | 1 | 1 |
| GACGAG | 1 | 0 |
| GACGAT | 1 | 1 |
| GACGCA | 0.6351 | 0 |
| GACGCC | 0.4621 | 0 |
| GACGCG | 0.1489 | 0 |
| GACGCT | 0.2913 | 0 |
| GACGGA | 0.7818 | 0 |
| GACGGC | 0.04996 | 0 |
| GACGGG | 0.3027 | 0 |
| GACGGT | 0.04996 | 0 |
| GACGTA | 0.6351 | 0 |
| GACGTC | 0.7818 | 0 |
| GACGTG | 1 | 1 |
| GACGTT | 0.7668 | 0 |
| GACTAA | 0.4621 | 0 |
| GACTAC | 0.6351 | 0 |
| GACTAG | 0 | 0 |
| GACTAT | 0.2913 | 0 |
| GACTCA | 0.7818 | 0 |
| GACTCC | 1 | 1 |
| GACTCG | 0.2913 | 0 |
| GACTCT | 0.6351 | 0 |
| GACTGA | 1 | 1 |
| GACTGC | 0.8854 | 0 |
| GACTGG | 1 | 1 |
| GACTGT | 0.8854 | 0 |
| GACTTA | 0.112 | 0 |
| GACTTC | 1 | 1 |
| GACTTG | 0.7818 | 0 |
| GACTTT | 0.1651 | 0 |
| GAGAAA | 1 | 1 |
| GAGAAC | 1 | 1 |
| GAGAAG | 1 | 1 |
| GAGAAT | 1 | 0 |
| GAGACA | 1 | 1 |
| GAGACC | 1 | 1 |
| GAGACG | 0.9919 | 0 |
| GAGACT | 0.978 | 0 |
| GAGAGA | 1 | 1 |
| GAGAGC | 0.978 | 0 |
| GAGAGG | 1 | 1 |
| GAGAGT | 1 | 1 |
| GAGATA | 1 | 1 |
| GAGATC | 1 | 1 |
| GAGATG | 1 | 1 |
| GAGATT | 0.8483 | 0 |
| GAGCAA | 1 | 0 |
| GAGCAC | 1 | 1 |
| GAGCAG | 1 | 1 |
| GAGCAT | 1 | 1 |
| GAGCCA | 0.7818 | 0 |
| GAGCCC | 0.8854 | 0 |
| GAGCCG | 0.3027 | 0 |
| GAGCCT | 1 | 1 |
| GAGCGA | 0.4621 | 0 |
| GAGCGC | 0.04996 | 0 |
| GAGCGG | 0.8854 | 0 |
| GAGCGT | 0.6351 | 0 |
| GAGCTA | 1 | 1 |
| GAGCTC | 0.978 | 0 |
| GAGCTG | 1 | 1 |
| GAGCTT | 1 | 1 |
| GAGGAA | 1 | 1 |
| GAGGAC | 1 | 1 |
| GAGGAG | 1 | 1 |
| GAGGAT | 1 | 1 |
| GAGGCA | 0.537 | 0 |
| GAGGCC | 1 | 1 |
| GAGGCG | 0.6351 | 0 |
| GAGGCT | 0.9468 | 0 |
| GAGGGA | 0.3364 | 0 |
| GAGGGC | 0.112 | 0 |
| GAGGGG | -0.5503 | 0 |
| GAGGGT | -0.4621 | 0 |
| GAGGTA | 0 | 0 |
| GAGGTC | 0.6351 | 0 |
| GAGGTG | 0.6351 | 0 |
| GAGGTT | 1 | 1 |
| GAGTAA | 0.1974 | 0 |
| GAGTAC | 0.6351 | 0 |
| GAGTAG | 0.4136 | 0 |
| GAGTAT | 0 | 0 |
| GAGTCA | 0.6351 | 0 |
| GAGTCC | 0.3027 | 0 |
| GAGTCG | 0.1974 | 0 |
| GAGTCT | 0.1489 | 0 |
| GAGTGA | 0.8854 | 0 |
| GAGTGC | 0.04996 | 0 |
| GAGTGG | 1 | 1 |
| GAGTGT | -0.2449 | 0 |
| GAGTTA | -0.2449 | 0 |
| GAGTTC | 0 | 0 |
| GAGTTG | 0.1651 | 0 |
| GAGTTT | -0.6351 | 0 |
| GATAAA | 0 | 0 |
| GATAAC | 0.2913 | 0 |
| GATAAG | 0.3746 | 0 |
| GATAAT | 0.2153 | 0 |
| GATACA | 0.2449 | 0 |
| GATACC | 0.2913 | 0 |
| GATACG | 0.1974 | 0 |
| GATACT | -0.1651 | 0 |
| GATAGA | 0.1651 | 0 |
| GATAGC | 0.112 | 0 |
| GATAGG | -1 | 1 |
| GATAGT | -0.3364 | 0 |
| GATATA | -0.3746 | 0 |
| GATATC | 1 | 1 |
| GATATG | 1 | 1 |
| GATATT | -0.2153 | 0 |
| GATCAA | 1 | 1 |
| GATCAC | 0.8854 | 0 |
| GATCAG | 1 | 1 |
| GATCAT | 1 | 1 |
| GATCCA | 0.9919 | 0 |
| GATCCC | 0.4621 | 0 |
| GATCCG | 0.5459 | 0 |
| GATCCT | 1 | 1 |
| GATCGA | 0.2913 | 0 |
| GATCGC | 0 | 0 |
| GATCGG | 0.2449 | 0 |
| GATCGT | 0.112 | 0 |
| GATCTA | 0.3027 | 0 |
| GATCTC | 0.4621 | 0 |
| GATCTG | 1 | 1 |
| GATCTT | 0.7668 | 0 |
| GATGAA | 1 | 1 |
| GATGAC | 0.9919 | 0 |
| GATGAG | 1 | 1 |
| GATGAT | 1 | 1 |
| GATGCA | 1 | 1 |
| GATGCC | 0.4621 | 0 |
| GATGCG | 0.3027 | 0 |
| GATGCT | 0.2913 | 0 |
| GATGGA | 1 | 1 |
| GATGGC | -0.1651 | 0 |
| GATGGG | -1 | 1 |
| GATGGT | -0.4621 | 0 |
| GATGTA | 0 | 0 |
| GATGTC | -0.2449 | 0 |
| GATGTG | 0.5503 | 0 |
| GATGTT | 0 | 0 |
| GATTAA | -0.3746 | 0 |
| GATTAC | -0.1489 | 0 |
| GATTAG | -1 | 1 |
| GATTAT | -0.4355 | 0 |
| GATTCA | 1 | 1 |
| GATTCC | 0.3027 | 0 |
| GATTCG | 0.1974 | 0 |
| GATTCT | 0.2449 | 0 |
| GATTGA | 0.8854 | 0 |
| GATTGC | 0 | 0 |
| GATTGG | -0.3746 | 0 |
| GATTGT | -0.2913 | 0 |
| GATTTA | -0.5459 | 0 |
| GATTTC | 0 | 0 |
| GATTTG | -0.3364 | 0 |
| GATTTT | -0.7818 | 0 |
| GCAAAA | 1 | 1 |
| GCAAAC | 0.8854 | 0 |
| GCAAAG | 0.8854 | 0 |
| GCAAAT | 0.3027 | 0 |
| GCAACA | 0.7818 | 0 |
| GCAACC | 0.6351 | 0 |
| GCAACG | 0.7818 | 0 |
| GCAACT | 0.8854 | 0 |
| GCAAGA | 1 | 1 |
| GCAAGC | 0.7818 | 0 |
| GCAAGG | 1 | 1 |
| GCAAGT | 0.5459 | 0 |
| GCAATA | 0.1489 | 0 |
| GCAATC | 0.2913 | 0 |
| GCAATG | 0.1651 | 0 |
| GCAATT | 0 | 0 |
| GCACAA | 0.4621 | 0 |
| GCACAC | 0.1651 | 0 |
| GCACAG | 0.4621 | 0 |
| GCACAT | 0 | 0 |
| GCACCA | 0.2913 | 0 |
| GCACCC | 0.2913 | 0 |
| GCACCG | 0.2913 | 0 |
| GCACCT | 0.7818 | 0 |
| GCACGA | 0.7818 | 0 |
| GCACGC | 0 | 0 |
| GCACGG | 0.2913 | 0 |
| GCACGT | 0.04996 | 0 |
| GCACTA | 0.112 | 0 |
| GCACTC | 0.2913 | 0 |
| GCACTG | 1 | 1 |
| GCACTT | 0.2913 | 0 |
| GCAGAA | 1 | 1 |
| GCAGAC | 1 | 1 |
| GCAGAG | 0.9919 | 0 |
| GCAGAT | 0.9468 | 0 |
| GCAGCA | 0.9973 | 0 |
| GCAGCC | 0.978 | 0 |
| GCAGCG | 0.6351 | 0 |
| GCAGCT | 1 | 1 |
| GCAGGA | 1 | 1 |
| GCAGGC | 0.2913 | 0 |
| GCAGGG | 0.1651 | 0 |
| GCAGGT | 0.3027 | 0 |
| GCAGTA | 0.6351 | 0 |
| GCAGTC | 0.4621 | 0 |
| GCAGTG | 0.9468 | 0 |
| GCAGTT | 0.3027 | 0 |
| GCATAA | 0 | 0 |
| GCATAC | -0.112 | 0 |
| GCATAG | -0.1974 | 0 |
| GCATAT | -0.4219 | 0 |
| GCATCA | 0.7818 | 0 |
| GCATCC | 0.2913 | 0 |
| GCATCG | 0.2913 | 0 |
| GCATCT | 1 | 1 |
| GCATGA | 0.3027 | 0 |
| GCATGC | -0.1489 | 0 |
| GCATGG | -0.1651 | 0 |
| GCATGT | -0.2449 | 0 |
| GCATTA | 0.112 | 0 |
| GCATTC | 0.2913 | 0 |
| GCATTG | 1 | 1 |
| GCATTT | 0 | 0 |
| GCCAAA | 0.1489 | 0 |
| GCCAAC | 0.6351 | 0 |
| GCCAAG | 0.4621 | 0 |
| GCCAAT | 0.1489 | 0 |
| GCCACA | 0 | 0 |
| GCCACC | 0.04996 | 0 |
| GCCACG | 0.2913 | 0 |
| GCCACT | -0.04996 | 0 |
| GCCAGA | 0.7818 | 0 |
| GCCAGC | 0.4621 | 0 |
| GCCAGG | 0.4621 | 0 |
| GCCAGT | 0.4621 | 0 |
| GCCATA | 0 | 0 |
| GCCATC | 0.1489 | 0 |
| GCCATG | 0.1651 | 0 |
| GCCATT | 0.1489 | 0 |
| GCCCAA | 0.04996 | 0 |
| GCCCAC | 0.04996 | 0 |
| GCCCAG | 0.6351 | 0 |
| GCCCAT | 0.04996 | 0 |
| GCCCCA | 0.04996 | 0 |
| GCCCCC | 0 | 0 |
| GCCCCG | 0.04996 | 0 |
| GCCCCT | 0.04996 | 0 |
| GCCCGA | 0.04996 | 0 |
| GCCCGC | 0 | 0 |
| GCCCGG | 0.6351 | 0 |
| GCCCGT | 0 | 0 |
| GCCCTA | 0.2913 | 0 |
| GCCCTC | 0.2913 | 0 |
| GCCCTG | 1 | 1 |
| GCCCTT | 0.7818 | 0 |
| GCCGAA | 0.9468 | 0 |
| GCCGAC | 1 | 1 |
| GCCGAG | 0.4621 | 0 |
| GCCGAT | 0.1489 | 0 |
| GCCGCA | 0 | 0 |
| GCCGCC | 0.04996 | 0 |
| GCCGCG | 0.04996 | 0 |
| GCCGCT | 0.04996 | 0 |
| GCCGGA | 0.7668 | 0 |
| GCCGGC | 0.1974 | 0 |
| GCCGGG | 0.1974 | 0 |
| GCCGGT | 0 | 0 |
| GCCGTA | 0.04996 | 0 |
| GCCGTC | 0.1489 | 0 |
| GCCGTG | 1 | 1 |
| GCCGTT | -0.1651 | 0 |
| GCCTAA | 0.04996 | 0 |
| GCCTAC | 0.04996 | 0 |
| GCCTAG | 0 | 0 |
| GCCTAT | 0.04996 | 0 |
| GCCTCA | 0.2913 | 0 |
| GCCTCC | 0.2913 | 0 |
| GCCTCG | 0.4621 | 0 |
| GCCTCT | 0.6351 | 0 |
| GCCTGA | 1 | 1 |
| GCCTGC | 0.978 | 0 |
| GCCTGG | 0.7668 | 0 |
| GCCTGT | 1 | 1 |
| GCCTTA | 0.1974 | 0 |
| GCCTTC | 0.4621 | 0 |
| GCCTTG | 1 | 1 |
| GCCTTT | 0.1974 | 0 |
| GCGAAA | 0.8854 | 0 |
| GCGAAC | 0.6351 | 0 |
| GCGAAG | 0.9468 | 0 |
| GCGAAT | 1 | 1 |
| GCGACA | 0.4621 | 0 |
| GCGACC | 0.4621 | 0 |
| GCGACG | 0.1489 | 0 |
| GCGACT | 0.6351 | 0 |
| GCGAGA | 0.2913 | 0 |
| GCGAGC | 0.1489 | 0 |
| GCGAGG | 0.4219 | 0 |
| GCGAGT | 0.1489 | 0 |
| GCGATA | 0.04996 | 0 |
| GCGATC | 0.2913 | 0 |
| GCGATG | 0 | 0 |
| GCGATT | 0.2913 | 0 |
| GCGCAA | 0.1489 | 0 |
| GCGCAC | 0.04996 | 0 |
| GCGCAG | 0.1489 | 0 |
| GCGCAT | 0.1489 | 0 |
| GCGCCA | 0 | 0 |
| GCGCCC | 0 | 0 |
| GCGCCG | 0 | 0 |
| GCGCCT | 0.1489 | 0 |
| GCGCGA | 0 | 0 |
| GCGCGC | 0 | 0 |
| GCGCGG | 0.04996 | 0 |
| GCGCGT | 0 | 0 |
| GCGCTA | 0.04996 | 0 |
| GCGCTC | 0 | 0 |
| GCGCTG | 0.7818 | 0 |
| GCGCTT | 0.04996 | 0 |
| GCGGAA | 1 | 1 |
| GCGGAC | 0.9468 | 0 |
| GCGGAG | 0.7668 | 0 |
| GCGGAT | 0.4219 | 0 |
| GCGGCA | 0 | 0 |
| GCGGCC | 0.1489 | 0 |
| GCGGCG | 0 | 0 |
| GCGGCT | 0.4621 | 0 |
| GCGGGA | 0.3027 | 0 |
| GCGGGC | 0 | 0 |
| GCGGGG | -0.2913 | 0 |
| GCGGGT | -0.1489 | 0 |
| GCGGTA | 0 | 0 |
| GCGGTC | 0 | 0 |
| GCGGTG | 0.1974 | 0 |
| GCGGTT | 0 | 0 |
| GCGTAA | 0 | 0 |
| GCGTAC | 0 | 0 |
| GCGTAG | -0.4621 | 0 |
| GCGTAT | 0.1489 | 0 |
| GCGTCA | 0.04996 | 0 |
| GCGTCC | 0 | 0 |
| GCGTCG | 0.2913 | 0 |
| GCGTCT | 0.04996 | 0 |
| GCGTGA | 0.4621 | 0 |
| GCGTGC | 0 | 0 |
| GCGTGG | 0.1974 | 0 |
| GCGTGT | 0 | 0 |
| GCGTTA | -0.1489 | 0 |
| GCGTTC | -0.7818 | 0 |
| GCGTTG | 0.112 | 0 |
| GCGTTT | -0.1489 | 0 |
| GCTAAA | 0.1489 | 0 |
| GCTAAC | 0.04996 | 0 |
| GCTAAG | -0.112 | 0 |
| GCTAAT | 0.04996 | 0 |
| GCTACA | 0.1489 | 0 |
| GCTACC | 0 | 0 |
| GCTACG | 0 | 0 |
| GCTACT | 0 | 0 |
| GCTAGA | 0.1489 | 0 |
| GCTAGC | 0 | 0 |
| GCTAGG | -0.664 | 0 |
| GCTAGT | -0.1489 | 0 |
| GCTATA | -0.04996 | 0 |
| GCTATC | 0.04996 | 0 |
| GCTATG | 0.1651 | 0 |
| GCTATT | -0.1974 | 0 |
| GCTCAA | 0.2913 | 0 |
| GCTCAC | 0.04996 | 0 |
| GCTCAG | 0.1974 | 0 |
| GCTCAT | 0 | 0 |
| GCTCCA | 0.1489 | 0 |
| GCTCCC | 0 | 0 |
| GCTCCG | 0 | 0 |
| GCTCCT | 0.1974 | 0 |
| GCTCGA | 0.4621 | 0 |
| GCTCGC | 0 | 0 |
| GCTCGG | 0.1489 | 0 |
| GCTCGT | -0.04996 | 0 |
| GCTCTA | 0.1651 | 0 |
| GCTCTC | 0.2913 | 0 |
| GCTCTG | 1 | 1 |
| GCTCTT | 0.3027 | 0 |
| GCTGAA | 1 | 1 |
| GCTGAC | 1 | 1 |
| GCTGAG | 1 | 1 |
| GCTGAT | 0.978 | 0 |
| GCTGCA | 0.8854 | 0 |
| GCTGCC | 0.6351 | 0 |
| GCTGCG | 0.2913 | 0 |
| GCTGCT | 0.8854 | 0 |
| GCTGGA | 1 | 1 |
| GCTGGC | 0.7818 | 0 |
| GCTGGG | 0.2636 | 0 |
| GCTGGT | 0.6351 | 0 |
| GCTGTA | 0.9468 | 0 |
| GCTGTC | 0.8854 | 0 |
| GCTGTG | 1 | 1 |
| GCTGTT | 1 | 1 |
| GCTTAA | -0.1974 | 0 |
| GCTTAC | -0.112 | 0 |
| GCTTAG | -1 | 1 |
| GCTTAT | -0.7818 | 0 |
| GCTTCA | 0.8854 | 0 |
| GCTTCC | -0.04996 | 0 |
| GCTTCG | 0.112 | 0 |
| GCTTCT | 0 | 0 |
| GCTTGA | 0.7818 | 0 |
| GCTTGC | 0 | 0 |
| GCTTGG | -0.2449 | 0 |
| GCTTGT | -0.112 | 0 |
| GCTTTA | -0.4621 | 0 |
| GCTTTC | -0.1489 | 0 |
| GCTTTG | 0 | 0 |
| GCTTTT | -0.4219 | 0 |
| GGAAAA | 1 | 1 |
| GGAAAC | 1 | 1 |
| GGAAAG | 1 | 1 |
| GGAAAT | 0.9074 | 0 |
| GGAACA | 1 | 1 |
| GGAACC | 1 | 1 |
| GGAACG | 0.9973 | 0 |
| GGAACT | 1 | 1 |
| GGAAGA | 1 | 1 |
| GGAAGC | 1 | 0 |
| GGAAGG | 0.664 | 0 |
| GGAAGT | 1 | 1 |
| GGAATA | 0.664 | 0 |
| GGAATC | 1 | 1 |
| GGAATG | 0.3552 | 0 |
| GGAATT | 0.6351 | 0 |
| GGACAA | 0.9998 | 0 |
| GGACAC | 1 | 1 |
| GGACAG | 1 | 1 |
| GGACAT | 0.9973 | 0 |
| GGACCA | 1 | 1 |
| GGACCC | 1 | 1 |
| GGACCG | 1 | 1 |
| GGACCT | 1 | 1 |
| GGACGA | 1 | 1 |
| GGACGC | 0.7818 | 0 |
| GGACGG | 0.4355 | 0 |
| GGACGT | 1 | 1 |
| GGACTA | 0.8854 | 0 |
| GGACTC | 1 | 1 |
| GGACTG | 1 | 1 |
| GGACTT | 0.9468 | 0 |
| GGAGAA | 1 | 1 |
| GGAGAC | 1 | 1 |
| GGAGAG | 1 | 1 |
| GGAGAT | 1 | 1 |
| GGAGCA | 1 | 1 |
| GGAGCC | 0.9973 | 0 |
| GGAGCG | 0.664 | 0 |
| GGAGCT | 1 | 1 |
| GGAGGA | 1 | 1 |
| GGAGGC | 0.8005 | 0 |
| GGAGGG | -1 | 1 |
| GGAGGT | 1 | 1 |
| GGAGTA | 1 | 1 |
| GGAGTC | 1 | 1 |
| GGAGTG | 0.4621 | 0 |
| GGAGTT | 0.537 | 0 |
| GGATAA | 0.1974 | 0 |
| GGATAC | 0.6351 | 0 |
| GGATAG | -0.2913 | 0 |
| GGATAT | 0.2449 | 0 |
| GGATCA | 1 | 1 |
| GGATCC | 1 | 1 |
| GGATCG | 0.5459 | 0 |
| GGATCT | 1 | 1 |
| GGATGA | 0.8617 | 0 |
| GGATGC | 0.3364 | 0 |
| GGATGG | -1 | 1 |
| GGATGT | 0.4136 | 0 |
| GGATTA | -0.2636 | 0 |
| GGATTC | 1 | 1 |
| GGATTG | 0.2153 | 0 |
| GGATTT | -0.2153 | 0 |
| GGCAAA | 0.4621 | 0 |
| GGCAAC | 0.2913 | 0 |
| GGCAAG | 0.4355 | 0 |
| GGCAAT | 0.04996 | 0 |
| GGCACA | 0.04996 | 0 |
| GGCACC | 0.04996 | 0 |
| GGCACG | -0.04996 | 0 |
| GGCACT | 0.2913 | 0 |
| GGCAGA | 0.8854 | 0 |
| GGCAGC | 0.4621 | 0 |
| GGCAGG | 0.2153 | 0 |
| GGCAGT | 0.3027 | 0 |
| GGCATA | -0.6351 | 0 |
| GGCATC | 0.2449 | 0 |
| GGCATG | -1 | 1 |
| GGCATT | 0 | 0 |
| GGCCAA | 0.04996 | 0 |
| GGCCAC | 0.04996 | 0 |
| GGCCAG | 0.2913 | 0 |
| GGCCAT | 0.04996 | 0 |
| GGCCCA | 0.1489 | 0 |
| GGCCCC | 0.05411 | 0 |
| GGCCCG | 0.04996 | 0 |
| GGCCCT | 0.6351 | 0 |
| GGCCGA | 0.4621 | 0 |
| GGCCGC | 0.04996 | 0 |
| GGCCGG | 0.112 | 0 |
| GGCCGT | 0.3027 | 0 |
| GGCCTA | -0.04996 | 0 |
| GGCCTC | 0.1489 | 0 |
| GGCCTG | 0.664 | 0 |
| GGCCTT | 0.1974 | 0 |
| GGCGAA | 0.6351 | 0 |
| GGCGAC | 0.6351 | 0 |
| GGCGAG | 0 | 0 |
| GGCGAT | 0.112 | 0 |
| GGCGCA | 0.04996 | 0 |
| GGCGCC | 0 | 0 |
| GGCGCG | 0 | 0 |
| GGCGCT | 0.04996 | 0 |
| GGCGGA | 0.5459 | 0 |
| GGCGGC | 0.04996 | 0 |
| GGCGGG | -0.1974 | 0 |
| GGCGGT | 0.1974 | 0 |
| GGCGTA | -0.112 | 0 |
| GGCGTC | 0.04996 | 0 |
| GGCGTG | -0.1651 | 0 |
| GGCGTT | -0.2913 | 0 |
| GGCTAA | -0.112 | 0 |
| GGCTAC | 0 | 0 |
| GGCTAG | -0.1489 | 0 |
| GGCTAT | -0.112 | 0 |
| GGCTCA | 0.1974 | 0 |
| GGCTCC | 0.2913 | 0 |
| GGCTCG | 0.04996 | 0 |
| GGCTCT | 0.5459 | 0 |
| GGCTGA | 1 | 1 |
| GGCTGC | 0.6351 | 0 |
| GGCTGG | 0.5503 | 0 |
| GGCTGT | 1 | 1 |
| GGCTTA | -1 | 1 |
| GGCTTC | 0 | 0 |
| GGCTTG | -0.4219 | 0 |
| GGCTTT | -1 | 1 |
| GGGAAA | 0.5807 | 0 |
| GGGAAC | 0.978 | 0 |
| GGGAAG | 0.4522 | 0 |
| GGGAAT | 0.3027 | 0 |
| GGGACA | 0.7818 | 0 |
| GGGACC | 0.9919 | 0 |
| GGGACG | 0.1651 | 0 |
| GGGACT | 0.6351 | 0 |
| GGGAGA | 0.6469 | 0 |
| GGGAGC | 0.2913 | 0 |
| GGGAGG | -1 | 1 |
| GGGAGT | 0 | 0 |
| GGGATA | -0.4219 | 0 |
| GGGATC | 0.537 | 0 |
| GGGATG | -1 | 1 |
| GGGATT | -0.2449 | 0 |
| GGGCAA | -0.04996 | 0 |
| GGGCAC | 0.1489 | 0 |
| GGGCAG | 0.2449 | 0 |
| GGGCAT | -0.1974 | 0 |
| GGGCCA | 0.04996 | 0 |
| GGGCCC | 0.05411 | 0 |
| GGGCCG | 0.1489 | 0 |
| GGGCCT | 0.1489 | 0 |
| GGGCGA | 0 | 0 |
| GGGCGC | 0 | 0 |
| GGGCGG | -0.1651 | 0 |
| GGGCGT | -0.112 | 0 |
| GGGCTA | -0.112 | 0 |
| GGGCTC | 0.1489 | 0 |
| GGGCTG | 0.4621 | 0 |
| GGGCTT | -0.3027 | 0 |
| GGGGAA | -0.3342 | 0 |
| GGGGAC | -0.2153 | 0 |
| GGGGAG | -1 | 1 |
| GGGGAT | -1 | 1 |
| GGGGCA | -0.1974 | 0 |
| GGGGCC | 0.1611 | 0 |
| GGGGCG | -0.2913 | 0 |
| GGGGCT | -0.1974 | 0 |
| GGGGGA | -1 | 1 |
| GGGGGC | -0.8136 | 0 |
| GGGGGG | -1 | 1 |
| GGGGGT | -1 | 1 |
| GGGGTA | -1 | 1 |
| GGGGTC | -0.5459 | 0 |
| GGGGTG | -1 | 1 |
| GGGGTT | -1 | 1 |
| GGGTAA | -1 | 1 |
| GGGTAC | -0.4621 | 0 |
| GGGTAG | -1 | 1 |
| GGGTAT | -0.9973 | 0 |
| GGGTCA | -0.112 | 0 |
| GGGTCC | 0.1489 | 0 |
| GGGTCG | -0.1974 | 0 |
| GGGTCT | -0.112 | 0 |
| GGGTGA | -0.493 | 0 |
| GGGTGC | -0.6351 | 0 |
| GGGTGG | -1 | 1 |
| GGGTGT | -1 | 1 |
| GGGTTA | -1 | 1 |
| GGGTTC | -0.4219 | 0 |
| GGGTTG | -1 | 1 |
| GGGTTT | -1 | 1 |
| GGTAAA | -0.4355 | 0 |
| GGTAAC | -0.1974 | 0 |
| GGTAAG | -1 | 1 |
| GGTAAT | -0.8854 | 0 |
| GGTACA | 0 | 0 |
| GGTACC | 0.04996 | 0 |
| GGTACG | -0.2913 | 0 |
| GGTACT | 0 | 0 |
| GGTAGA | -0.3103 | 0 |
| GGTAGC | -0.6351 | 0 |
| GGTAGG | -1 | 1 |
| GGTAGT | -1 | 1 |
| GGTATA | -1 | 1 |
| GGTATC | -0.1651 | 0 |
| GGTATG | -1 | 1 |
| GGTATT | -0.9468 | 0 |
| GGTCAA | 0.6351 | 0 |
| GGTCAC | 0.04996 | 0 |
| GGTCAG | 0 | 0 |
| GGTCAT | -0.3027 | 0 |
| GGTCCA | 0.2913 | 0 |
| GGTCCC | 0.1489 | 0 |
| GGTCCG | -0.1651 | 0 |
| GGTCCT | 0.4219 | 0 |
| GGTCGA | 0.4621 | 0 |
| GGTCGC | 0.04996 | 0 |
| GGTCGG | -0.2913 | 0 |
| GGTCGT | -0.3027 | 0 |
| GGTCTA | -0.2913 | 0 |
| GGTCTC | 0.04996 | 0 |
| GGTCTG | -0.2153 | 0 |
| GGTCTT | -0.1489 | 0 |
| GGTGAA | 1 | 1 |
| GGTGAC | 0.8854 | 0 |
| GGTGAG | 0.5251 | 0 |
| GGTGAT | 0.3364 | 0 |
| GGTGCA | 0.7818 | 0 |
| GGTGCC | 0 | 0 |
| GGTGCG | 0 | 0 |
| GGTGCT | 0.2913 | 0 |
| GGTGGA | 1 | 1 |
| GGTGGC | -0.2636 | 0 |
| GGTGGG | -1 | 1 |
| GGTGGT | -0.4621 | 0 |
| GGTGTA | -0.4621 | 0 |
| GGTGTC | 0 | 0 |
| GGTGTG | -1 | 1 |
| GGTGTT | -0.8483 | 0 |
| GGTTAA | -0.8483 | 0 |
| GGTTAC | -0.6351 | 0 |
| GGTTAG | -1 | 1 |
| GGTTAT | -1 | 1 |
| GGTTCA | -0.2449 | 0 |
| GGTTCC | -0.4219 | 0 |
| GGTTCG | -0.7818 | 0 |
| GGTTCT | -0.664 | 0 |
| GGTTGA | -0.1651 | 0 |
| GGTTGC | -0.1489 | 0 |
| GGTTGG | -1 | 1 |
| GGTTGT | -0.8483 | 0 |
| GGTTTA | -1 | 1 |
| GGTTTC | -0.9074 | 0 |
| GGTTTG | -1 | 1 |
| GGTTTT | -1 | 1 |
| GTAAAA | -1 | 1 |
| GTAAAC | 0.2449 | 0 |
| GTAAAG | 0 | 0 |
| GTAAAT | -0.7668 | 0 |
| GTAACA | 0 | 0 |
| GTAACC | 0.2913 | 0 |
| GTAACG | -0.112 | 0 |
| GTAACT | -0.1651 | 0 |
| GTAAGA | 0 | 0 |
| GTAAGC | -0.1974 | 0 |
| GTAAGG | -1 | 1 |
| GTAAGT | -1 | 1 |
| GTAATA | -0.6351 | 0 |
| GTAATC | 0 | 0 |
| GTAATG | -1 | 1 |
| GTAATT | -0.9919 | 0 |
| GTACAA | 0.3027 | 0 |
| GTACAC | 0.1489 | 0 |
| GTACAG | 0.4219 | 0 |
| GTACAT | -0.2449 | 0 |
| GTACCA | 0.1489 | 0 |
| GTACCC | 0.1489 | 0 |
| GTACCG | 0 | 0 |
| GTACCT | 0.1974 | 0 |
| GTACGA | 0.4621 | 0 |
| GTACGC | 0 | 0 |
| GTACGG | -0.112 | 0 |
| GTACGT | -0.4219 | 0 |
| GTACTA | -0.4621 | 0 |
| GTACTC | 0.04996 | 0 |
| GTACTG | 0.3364 | 0 |
| GTACTT | -0.3027 | 0 |
| GTAGAA | 1 | 1 |
| GTAGAC | 0.978 | 0 |
| GTAGAG | 0.4355 | 0 |
| GTAGAT | 0 | 0 |
| GTAGCA | 0.3027 | 0 |
| GTAGCC | 0.1489 | 0 |
| GTAGCG | 0 | 0 |
| GTAGCT | 0.1651 | 0 |
| GTAGGA | 1 | 1 |
| GTAGGC | -0.664 | 0 |
| GTAGGG | -1 | 1 |
| GTAGGT | -1 | 1 |
| GTAGTA | -0.2636 | 0 |
| GTAGTC | -0.1974 | 0 |
| GTAGTG | -0.7668 | 0 |
| GTAGTT | -1 | 1 |
| GTATAA | -0.9468 | 0 |
| GTATAC | -0.7818 | 0 |
| GTATAG | -1 | 1 |
| GTATAT | -1 | 1 |
| GTATCA | 0.1489 | 0 |
| GTATCC | 0 | 0 |
| GTATCG | 0 | 0 |
| GTATCT | -0.4355 | 0 |
| GTATGA | 0 | 0 |
| GTATGC | -0.664 | 0 |
| GTATGG | -0.9081 | 0 |
| GTATGT | -1 | 1 |
| GTATTA | -0.8854 | 0 |
| GTATTC | -0.1974 | 0 |
| GTATTG | -0.664 | 0 |
| GTATTT | -1 | 1 |
| GTCAAA | 0.5459 | 0 |
| GTCAAC | 1 | 1 |
| GTCAAG | 1 | 1 |
| GTCAAT | 0.6351 | 0 |
| GTCACA | 0 | 0 |
| GTCACC | 0.1489 | 0 |
| GTCACG | 0 | 0 |
| GTCACT | 0 | 0 |
| GTCAGA | 0.2913 | 0 |
| GTCAGC | 0.7818 | 0 |
| GTCAGG | 0.2153 | 0 |
| GTCAGT | -0.2913 | 0 |
| GTCATA | -0.4621 | 0 |
| GTCATC | 0.1651 | 0 |
| GTCATG | -1 | 1 |
| GTCATT | -0.664 | 0 |
| GTCCAA | 0.6351 | 0 |
| GTCCAC | 0.6351 | 0 |
| GTCCAG | 1 | 1 |
| GTCCAT | 0.1651 | 0 |
| GTCCCA | 0.04996 | 0 |
| GTCCCC | 0 | 0 |
| GTCCCG | 0.1974 | 0 |
| GTCCCT | -0.112 | 0 |
| GTCCGA | -0.1489 | 0 |
| GTCCGC | -0.1489 | 0 |
| GTCCGG | 0.537 | 0 |
| GTCCGT | -1 | 1 |
| GTCCTA | 0.112 | 0 |
| GTCCTC | 0.6351 | 0 |
| GTCCTG | 1 | 1 |
| GTCCTT | 0 | 0 |
| GTCGAA | 1 | 1 |
| GTCGAC | 1 | 1 |
| GTCGAG | 0.8854 | 0 |
| GTCGAT | 0.1974 | 0 |
| GTCGCA | 0.112 | 0 |
| GTCGCC | 0.2913 | 0 |
| GTCGCG | 0.04996 | 0 |
| GTCGCT | 0 | 0 |
| GTCGGA | 0.2913 | 0 |
| GTCGGC | 0.2913 | 0 |
| GTCGGG | -0.2913 | 0 |
| GTCGGT | -0.3027 | 0 |
| GTCGTA | -0.1974 | 0 |
| GTCGTC | 0.1651 | 0 |
| GTCGTG | 0 | 0 |
| GTCGTT | -1 | 1 |
| GTCTAA | 0 | 0 |
| GTCTAC | 0.1489 | 0 |
| GTCTAG | -0.3364 | 0 |
| GTCTAT | -0.4621 | 0 |
| GTCTCA | 0 | 0 |
| GTCTCC | 0.112 | 0 |
| GTCTCG | 0.04996 | 0 |
| GTCTCT | -0.4621 | 0 |
| GTCTGA | 0.7818 | 0 |
| GTCTGC | 0.4621 | 0 |
| GTCTGG | 0.1974 | 0 |
| GTCTGT | -0.3364 | 0 |
| GTCTTA | -0.6351 | 0 |
| GTCTTC | 0.1974 | 0 |
| GTCTTG | -0.1651 | 0 |
| GTCTTT | -0.978 | 0 |
| GTGAAA | 0.9468 | 0 |
| GTGAAC | 1 | 1 |
| GTGAAG | 1 | 1 |
| GTGAAT | 0.7818 | 0 |
| GTGACA | 0.1974 | 0 |
| GTGACC | 1 | 1 |
| GTGACG | 0.9468 | 0 |
| GTGACT | 0.2913 | 0 |
| GTGAGA | 0.7818 | 0 |
| GTGAGC | 0.8854 | 0 |
| GTGAGG | 1 | 1 |
| GTGAGT | 0.2153 | 0 |
| GTGATA | -0.112 | 0 |
| GTGATC | 0.4621 | 0 |
| GTGATG | 0.3364 | 0 |
| GTGATT | -0.2913 | 0 |
| GTGCAA | 0.4621 | 0 |
| GTGCAC | 0.6351 | 0 |
| GTGCAG | 1 | 1 |
| GTGCAT | 0 | 0 |
| GTGCCA | 0 | 0 |
| GTGCCC | 0.4621 | 0 |
| GTGCCG | 0.3027 | 0 |
| GTGCCT | 0 | 0 |
| GTGCGA | 0.4621 | 0 |
| GTGCGC | 0.1489 | 0 |
| GTGCGG | 1 | 1 |
| GTGCGT | -0.1651 | 0 |
| GTGCTA | 0.1651 | 0 |
| GTGCTC | 0.4621 | 0 |
| GTGCTG | 1 | 1 |
| GTGCTT | 0 | 0 |
| GTGGAA | 1 | 1 |
| GTGGAC | 1 | 1 |
| GTGGAG | 1 | 1 |
| GTGGAT | 0.8854 | 0 |
| GTGGCA | -1 | 1 |
| GTGGCC | 1 | 1 |
| GTGGCG | 0.537 | 0 |
| GTGGCT | 0.2153 | 0 |
| GTGGGA | -0.3746 | 0 |
| GTGGGC | -0.1651 | 0 |
| GTGGGG | -1 | 1 |
| GTGGGT | -1 | 1 |
| GTGGTA | -0.5459 | 0 |
| GTGGTC | 0.3027 | 0 |
| GTGGTG | 0 | 0 |
| GTGGTT | -1 | 1 |
| GTGTAA | -0.7668 | 0 |
| GTGTAC | 0 | 0 |
| GTGTAG | -1 | 1 |
| GTGTAT | -0.9919 | 0 |
| GTGTCA | -0.3027 | 0 |
| GTGTCC | 0.3027 | 0 |
| GTGTCG | 1 | 1 |
| GTGTCT | -0.3027 | 0 |
| GTGTGA | -0.1489 | 0 |
| GTGTGC | 0.3027 | 0 |
| GTGTGG | 0.4522 | 0 |
| GTGTGT | -0.9816 | 0 |
| GTGTTA | -1 | 1 |
| GTGTTC | -0.8854 | 0 |
| GTGTTG | -0.6351 | 0 |
| GTGTTT | -1 | 1 |
| GTTAAA | -0.8854 | 0 |
| GTTAAC | -0.2449 | 0 |
| GTTAAG | -0.3364 | 0 |
| GTTAAT | -0.9919 | 0 |
| GTTACA | -0.04996 | 0 |
| GTTACC | -0.112 | 0 |
| GTTACG | -0.2913 | 0 |
| GTTACT | -0.7818 | 0 |
| GTTAGA | -0.5459 | 0 |
| GTTAGC | -0.6351 | 0 |
| GTTAGG | -1 | 1 |
| GTTAGT | -1 | 1 |
| GTTATA | -0.978 | 0 |
| GTTATC | -0.3027 | 0 |
| GTTATG | -1 | 1 |
| GTTATT | -1 | 1 |
| GTTCAA | -0.3364 | 0 |
| GTTCAC | -0.1974 | 0 |
| GTTCAG | 0.3552 | 0 |
| GTTCAT | -1 | 1 |
| GTTCCA | -0.6351 | 0 |
| GTTCCC | -0.6351 | 0 |
| GTTCCG | -1 | 1 |
| GTTCCT | -1 | 1 |
| GTTCGA | -0.2153 | 0 |
| GTTCGC | -0.2913 | 0 |
| GTTCGG | -0.7668 | 0 |
| GTTCGT | -1 | 1 |
| GTTCTA | -1 | 1 |
| GTTCTC | -0.7818 | 0 |
| GTTCTG | -1 | 1 |
| GTTCTT | -0.9998 | 0 |
| GTTGAA | 0.9468 | 0 |
| GTTGAC | 0.6351 | 0 |
| GTTGAG | 0.4355 | 0 |
| GTTGAT | -0.1974 | 0 |
| GTTGCA | 0.112 | 0 |
| GTTGCC | 0 | 0 |
| GTTGCG | -0.112 | 0 |
| GTTGCT | -0.4621 | 0 |
| GTTGGA | 1 | 1 |
| GTTGGC | -0.112 | 0 |
| GTTGGG | -1 | 1 |
| GTTGGT | -0.7668 | 0 |
| GTTGTA | -0.5459 | 0 |
| GTTGTC | -0.1489 | 0 |
| GTTGTG | -0.7668 | 0 |
| GTTGTT | -1 | 1 |
| GTTTAA | -1 | 1 |
| GTTTAC | -0.9973 | 0 |
| GTTTAG | -1 | 1 |
| GTTTAT | -1 | 1 |
| GTTTCA | -0.664 | 0 |
| GTTTCC | -1 | 1 |
| GTTTCG | -0.8483 | 0 |
| GTTTCT | -1 | 1 |
| GTTTGA | -0.5459 | 0 |
| GTTTGC | -0.8854 | 0 |
| GTTTGG | -1 | 1 |
| GTTTGT | -1 | 1 |
| GTTTTA | -0.9999 | 0 |
| GTTTTC | -0.978 | 0 |
| GTTTTG | -1 | 1 |
| GTTTTT | -1 | 1 |
| TAAAAA | -1 | 1 |
| TAAAAC | 0 | 0 |
| TAAAAG | 0.4219 | 0 |
| TAAAAT | -1 | 1 |
| TAAACA | 0.2913 | 0 |
| TAAACC | 0.8854 | 0 |
| TAAACG | 0.1974 | 0 |
| TAAACT | 0.2153 | 0 |
| TAAAGA | 1 | 1 |
| TAAAGC | 0.5459 | 0 |
| TAAAGG | 0.2153 | 0 |
| TAAAGT | 0 | 0 |
| TAAATA | -1 | 1 |
| TAAATC | -0.3364 | 0 |
| TAAATG | -1 | 1 |
| TAAATT | -0.978 | 0 |
| TAACAA | 0 | 0 |
| TAACAC | 0.112 | 0 |
| TAACAG | 0.3027 | 0 |
| TAACAT | -0.1651 | 0 |
| TAACCA | 0.5459 | 0 |
| TAACCC | 0.2913 | 0 |
| TAACCG | 0.04996 | 0 |
| TAACCT | 0.3027 | 0 |
| TAACGA | 0.537 | 0 |
| TAACGC | -0.04996 | 0 |
| TAACGG | 0 | 0 |
| TAACGT | -0.112 | 0 |
| TAACTA | -1 | 1 |
| TAACTC | 0 | 0 |
| TAACTG | 0.3364 | 0 |
| TAACTT | -0.2913 | 0 |
| TAAGAA | 0.9074 | 0 |
| TAAGAC | 0.9468 | 0 |
| TAAGAG | 0.3027 | 0 |
| TAAGAT | 0.3103 | 0 |
| TAAGCA | 0.5459 | 0 |
| TAAGCC | 0.6351 | 0 |
| TAAGCG | 0.1974 | 0 |
| TAAGCT | 0.3746 | 0 |
| TAAGGA | 0.7818 | 0 |
| TAAGGC | -0.3027 | 0 |
| TAAGGG | -0.9468 | 0 |
| TAAGGT | -1 | 1 |
| TAAGTA | -0.6469 | 0 |
| TAAGTC | -0.4621 | 0 |
| TAAGTG | -1 | 1 |
| TAAGTT | -1 | 1 |
| TAATAA | -0.978 | 0 |
| TAATAC | -0.1974 | 0 |
| TAATAG | -0.4621 | 0 |
| TAATAT | -1 | 1 |
| TAATCA | 0.3364 | 0 |
| TAATCC | 0.112 | 0 |
| TAATCG | -0.04996 | 0 |
| TAATCT | -0.4621 | 0 |
| TAATGA | -1 | 1 |
| TAATGC | -1 | 1 |
| TAATGG | -1 | 1 |
| TAATGT | -1 | 1 |
| TAATTA | -1 | 1 |
| TAATTC | -0.7818 | 0 |
| TAATTG | -0.978 | 0 |
| TAATTT | -1 | 1 |
| TACAAA | 0.2636 | 0 |
| TACAAC | 1 | 1 |
| TACAAG | 1 | 1 |
| TACAAT | 0.1651 | 0 |
| TACACA | -0.1974 | 0 |
| TACACC | 0.2913 | 0 |
| TACACG | 0.1489 | 0 |
| TACACT | -0.04996 | 0 |
| TACAGA | 1 | 1 |
| TACAGC | 0.6351 | 0 |
| TACAGG | 0.3027 | 0 |
| TACAGT | 0 | 0 |
| TACATA | -1 | 1 |
| TACATC | 0.2153 | 0 |
| TACATG | 0 | 0 |
| TACATT | -0.2913 | 0 |
| TACCAA | 0.6351 | 0 |
| TACCAC | 0.2913 | 0 |
| TACCAG | 0.664 | 0 |
| TACCAT | 0.1489 | 0 |
| TACCCA | 0.2913 | 0 |
| TACCCC | 0 | 0 |
| TACCCG | 0.1489 | 0 |
| TACCCT | 0.7818 | 0 |
| TACCGA | 0.6351 | 0 |
| TACCGC | 0 | 0 |
| TACCGG | 0.4621 | 0 |
| TACCGT | -0.04996 | 0 |
| TACCTA | -0.112 | 0 |
| TACCTC | 0.2913 | 0 |
| TACCTG | 1 | 1 |
| TACCTT | -0.2153 | 0 |
| TACGAA | 0.978 | 0 |
| TACGAC | 1 | 1 |
| TACGAG | 0.3027 | 0 |
| TACGAT | 0.4621 | 0 |
| TACGCA | 0 | 0 |
| TACGCC | 0.04996 | 0 |
| TACGCG | 0 | 0 |
| TACGCT | -0.04996 | 0 |
| TACGGA | 0.6351 | 0 |
| TACGGC | 0.1489 | 0 |
| TACGGG | -0.112 | 0 |
| TACGGT | -0.2913 | 0 |
| TACGTA | -0.4621 | 0 |
| TACGTC | 0 | 0 |
| TACGTG | 0.3027 | 0 |
| TACGTT | -0.7818 | 0 |
| TACTAA | -0.4621 | 0 |
| TACTAC | 0.2153 | 0 |
| TACTAG | -1 | 1 |
| TACTAT | -0.9468 | 0 |
| TACTCA | 0.04996 | 0 |
| TACTCC | 0.04996 | 0 |
| TACTCG | 0 | 0 |
| TACTCT | 0 | 0 |
| TACTGA | 0.8483 | 0 |
| TACTGC | 0.112 | 0 |
| TACTGG | 0 | 0 |
| TACTGT | -0.3364 | 0 |
| TACTTA | -0.8854 | 0 |
| TACTTC | 0.2153 | 0 |
| TACTTG | -0.2449 | 0 |
| TACTTT | -1 | 1 |
| TAGAAA | 0.8005 | 0 |
| TAGAAC | 0.9998 | 0 |
| TAGAAG | 1 | 1 |
| TAGAAT | 0.4621 | 0 |
| TAGACA | 0.9468 | 0 |
| TAGACC | 1 | 1 |
| TAGACG | 0.6351 | 0 |
| TAGACT | 0.3027 | 0 |
| TAGAGA | 0.9919 | 0 |
| TAGAGC | 0.2913 | 0 |
| TAGAGG | 0.5503 | 0 |
| TAGAGT | -0.1974 | 0 |
| TAGATA | -0.537 | 0 |
| TAGATC | 0.3746 | 0 |
| TAGATG | 0.3799 | 0 |
| TAGATT | -0.7818 | 0 |
| TAGCAA | 0.4621 | 0 |
| TAGCAC | 0.1489 | 0 |
| TAGCAG | 0.978 | 0 |
| TAGCAT | 0.1974 | 0 |
| TAGCCA | 0.04996 | 0 |
| TAGCCC | 0.2913 | 0 |
| TAGCCG | 0.112 | 0 |
| TAGCCT | 0.4621 | 0 |
| TAGCGA | 0.1489 | 0 |
| TAGCGC | 0 | 0 |
| TAGCGG | 0 | 0 |
| TAGCGT | -0.112 | 0 |
| TAGCTA | -0.1651 | 0 |
| TAGCTC | 0.2913 | 0 |
| TAGCTG | 0.9468 | 0 |
| TAGCTT | -0.1974 | 0 |
| TAGGAA | 0.9074 | 0 |
| TAGGAC | 0.978 | 0 |
| TAGGAG | 0.8005 | 0 |
| TAGGAT | 0.3746 | 0 |
| TAGGCA | -0.4621 | 0 |
| TAGGCC | 0.112 | 0 |
| TAGGCG | -0.1489 | 0 |
| TAGGCT | -0.2449 | 0 |
| TAGGGA | -0.6351 | 0 |
| TAGGGC | -0.7818 | 0 |
| TAGGGG | -1 | 1 |
| TAGGGT | -1 | 1 |
| TAGGTA | -1 | 1 |
| TAGGTC | -0.7818 | 0 |
| TAGGTG | -0.8483 | 0 |
| TAGGTT | -1 | 1 |
| TAGTAA | -0.4621 | 0 |
| TAGTAC | -0.112 | 0 |
| TAGTAG | -0.3746 | 0 |
| TAGTAT | -1 | 1 |
| TAGTCA | 0.112 | 0 |
| TAGTCC | 0.112 | 0 |
| TAGTCG | -0.112 | 0 |
| TAGTCT | -0.4621 | 0 |
| TAGTGA | 0 | 0 |
| TAGTGC | -0.4621 | 0 |
| TAGTGG | -1 | 1 |
| TAGTGT | -1 | 1 |
| TAGTTA | -1 | 1 |
| TAGTTC | -0.9919 | 0 |
| TAGTTG | -0.978 | 0 |
| TAGTTT | -1 | 1 |
| TATAAA | -1 | 1 |
| TATAAC | -0.5459 | 0 |
| TATAAG | -0.4355 | 0 |
| TATAAT | -1 | 1 |
| TATACA | -0.8854 | 0 |
| TATACC | -0.1974 | 0 |
| TATACG | -0.1974 | 0 |
| TATACT | -1 | 1 |
| TATAGA | -0.4136 | 0 |
| TATAGC | -0.6351 | 0 |
| TATAGG | -1 | 1 |
| TATAGT | -1 | 1 |
| TATATA | -1 | 1 |
| TATATC | -1 | 1 |
| TATATG | -1 | 1 |
| TATATT | -1 | 1 |
| TATCAA | 0.4355 | 0 |
| TATCAC | 0 | 0 |
| TATCAG | 0.6351 | 0 |
| TATCAT | -0.4219 | 0 |
| TATCCA | 0.112 | 0 |
| TATCCC | 0 | 0 |
| TATCCG | 0.04996 | 0 |
| TATCCT | -0.2913 | 0 |
| TATCGA | 0.04996 | 0 |
| TATCGC | 0 | 0 |
| TATCGG | -0.112 | 0 |
| TATCGT | -0.6351 | 0 |
| TATCTA | -0.9468 | 0 |
| TATCTC | -0.5459 | 0 |
| TATCTG | 0.3364 | 0 |
| TATCTT | -1 | 1 |
| TATGAA | 1 | 1 |
| TATGAC | 0.3027 | 0 |
| TATGAG | 0.1974 | 0 |
| TATGAT | -0.4621 | 0 |
| TATGCA | -1 | 1 |
| TATGCC | -0.6351 | 0 |
| TATGCG | -0.4219 | 0 |
| TATGCT | -1 | 1 |
| TATGGA | 1 | 1 |
| TATGGC | -0.2153 | 0 |
| TATGGG | -1 | 1 |
| TATGGT | -1 | 1 |
| TATGTA | -1 | 1 |
| TATGTC | -1 | 1 |
| TATGTG | -0.9468 | 0 |
| TATGTT | -1 | 1 |
| TATTAA | -1 | 1 |
| TATTAC | -0.6351 | 0 |
| TATTAG | -0.9919 | 0 |
| TATTAT | -1 | 1 |
| TATTCA | -0.3746 | 0 |
| TATTCC | -0.2913 | 0 |
| TATTCG | -0.1489 | 0 |
| TATTCT | -1 | 1 |
| TATTGA | 0 | 0 |
| TATTGC | -0.4621 | 0 |
| TATTGG | -0.7668 | 0 |
| TATTGT | -1 | 1 |
| TATTTA | -1 | 1 |
| TATTTC | -1 | 1 |
| TATTTG | -1 | 1 |
| TATTTT | -1 | 1 |
| TCAAAA | 0.4621 | 0 |
| TCAAAC | 0.7818 | 0 |
| TCAAAG | 0.8854 | 0 |
| TCAAAT | -0.3027 | 0 |
| TCAACA | 0.8483 | 0 |
| TCAACC | 1 | 1 |
| TCAACG | 1 | 1 |
| TCAACT | 0.5459 | 0 |
| TCAAGA | 1 | 1 |
| TCAAGC | 0.9919 | 0 |
| TCAAGG | 1 | 1 |
| TCAAGT | 0.664 | 0 |
| TCAATA | -0.1651 | 0 |
| TCAATC | 0.2913 | 0 |
| TCAATG | 0.1651 | 0 |
| TCAATT | -0.1974 | 0 |
| TCACAA | 0.1489 | 0 |
| TCACAC | -0.1489 | 0 |
| TCACAG | 0.04996 | 0 |
| TCACAT | -0.1489 | 0 |
| TCACCA | 0.4621 | 0 |
| TCACCC | 0.2913 | 0 |
| TCACCG | 0.1489 | 0 |
| TCACCT | 0.4621 | 0 |
| TCACGA | 0.6351 | 0 |
| TCACGC | 0 | 0 |
| TCACGG | 0.2913 | 0 |
| TCACGT | 0 | 0 |
| TCACTA | -0.6351 | 0 |
| TCACTC | 0 | 0 |
| TCACTG | 0.2913 | 0 |
| TCACTT | -0.1489 | 0 |
| TCAGAA | 1 | 1 |
| TCAGAC | 0.9468 | 0 |
| TCAGAG | 0.7818 | 0 |
| TCAGAT | 0.3364 | 0 |
| TCAGCA | 1 | 1 |
| TCAGCC | 1 | 1 |
| TCAGCG | 0.8854 | 0 |
| TCAGCT | 1 | 1 |
| TCAGGA | 1 | 1 |
| TCAGGC | 0.3027 | 0 |
| TCAGGG | 0.1974 | 0 |
| TCAGGT | -0.1651 | 0 |
| TCAGTA | 0.1651 | 0 |
| TCAGTC | 0.1489 | 0 |
| TCAGTG | 0 | 0 |
| TCAGTT | -0.4219 | 0 |
| TCATAA | -0.3364 | 0 |
| TCATAC | -0.2913 | 0 |
| TCATAG | -0.1489 | 0 |
| TCATAT | -1 | 1 |
| TCATCA | 1 | 1 |
| TCATCC | 0.2913 | 0 |
| TCATCG | 0.4621 | 0 |
| TCATCT | 0.2913 | 0 |
| TCATGA | 0.3103 | 0 |
| TCATGC | -0.5459 | 0 |
| TCATGG | -0.4219 | 0 |
| TCATGT | -1 | 1 |
| TCATTA | -1 | 1 |
| TCATTC | -0.4621 | 0 |
| TCATTG | 0 | 0 |
| TCATTT | -1 | 1 |
| TCCAAA | 0.4621 | 0 |
| TCCAAC | 1 | 1 |
| TCCAAG | 0.8854 | 0 |
| TCCAAT | 0.1489 | 0 |
| TCCACA | 0.6351 | 0 |
| TCCACC | 0.6351 | 0 |
| TCCACG | 1 | 1 |
| TCCACT | 0.112 | 0 |
| TCCAGA | 1 | 1 |
| TCCAGC | 0.9992 | 0 |
| TCCAGG | 0.7668 | 0 |
| TCCAGT | 0.7818 | 0 |
| TCCATA | -0.1651 | 0 |
| TCCATC | 0.2913 | 0 |
| TCCATG | 0.112 | 0 |
| TCCATT | -0.1651 | 0 |
| TCCCAA | 0 | 0 |
| TCCCAC | 0.04996 | 0 |
| TCCCAG | 0.2913 | 0 |
| TCCCAT | 0 | 0 |
| TCCCCA | 0.04996 | 0 |
| TCCCCC | 0 | 0 |
| TCCCCG | 0.2913 | 0 |
| TCCCCT | 0.04996 | 0 |
| TCCCGA | 0.2913 | 0 |
| TCCCGC | 0.2913 | 0 |
| TCCCGG | 1 | 1 |
| TCCCGT | 0.112 | 0 |
| TCCCTA | -0.04996 | 0 |
| TCCCTC | 0.04996 | 0 |
| TCCCTG | 0.664 | 0 |
| TCCCTT | -0.1651 | 0 |
| TCCGAA | 0.6351 | 0 |
| TCCGAC | 0.9468 | 0 |
| TCCGAG | 0.1489 | 0 |
| TCCGAT | -0.04996 | 0 |
| TCCGCA | 0.2913 | 0 |
| TCCGCC | 0.1489 | 0 |
| TCCGCG | 0.2913 | 0 |
| TCCGCT | 0.1974 | 0 |
| TCCGGA | 0.9998 | 0 |
| TCCGGC | 1 | 1 |
| TCCGGG | 1 | 1 |
| TCCGGT | 0.3027 | 0 |
| TCCGTA | -0.4621 | 0 |
| TCCGTC | -0.1974 | 0 |
| TCCGTG | -0.1651 | 0 |
| TCCGTT | -1 | 1 |
| TCCTAA | 0.1489 | 0 |
| TCCTAC | 0.1489 | 0 |
| TCCTAG | -0.112 | 0 |
| TCCTAT | -0.1974 | 0 |
| TCCTCA | 1 | 1 |
| TCCTCC | 0.6351 | 0 |
| TCCTCG | 1 | 1 |
| TCCTCT | 0.664 | 0 |
| TCCTGA | 1 | 1 |
| TCCTGC | 1 | 1 |
| TCCTGG | 0.9081 | 0 |
| TCCTGT | 1 | 1 |
| TCCTTA | -0.2449 | 0 |
| TCCTTC | 0 | 0 |
| TCCTTG | 0.5459 | 0 |
| TCCTTT | -0.5459 | 0 |
| TCGAAA | 1 | 1 |
| TCGAAC | 0.9973 | 0 |
| TCGAAG | 1 | 1 |
| TCGAAT | 0.9468 | 0 |
| TCGACA | 1 | 1 |
| TCGACC | 0.9468 | 0 |
| TCGACG | 0.9468 | 0 |
| TCGACT | 0.8854 | 0 |
| TCGAGA | 0.9468 | 0 |
| TCGAGC | 1 | 1 |
| TCGAGG | 0.7668 | 0 |
| TCGAGT | 0.1489 | 0 |
| TCGATA | 0.1489 | 0 |
| TCGATC | 0.3027 | 0 |
| TCGATG | 0.1489 | 0 |
| TCGATT | 0.112 | 0 |
| TCGCAA | 0.1489 | 0 |
| TCGCAC | 0 | 0 |
| TCGCAG | 0.1489 | 0 |
| TCGCAT | 0 | 0 |
| TCGCCA | 0.1489 | 0 |
| TCGCCC | 0 | 0 |
| TCGCCG | 0.1489 | 0 |
| TCGCCT | 0.1489 | 0 |
| TCGCGA | 0.04996 | 0 |
| TCGCGC | 0.04996 | 0 |
| TCGCGG | 0.1489 | 0 |
| TCGCGT | 0 | 0 |
| TCGCTA | -0.04996 | 0 |
| TCGCTC | -0.04996 | 0 |
| TCGCTG | 0.1489 | 0 |
| TCGCTT | -0.04996 | 0 |
| TCGGAA | 0.9468 | 0 |
| TCGGAC | 0.2913 | 0 |
| TCGGAG | 0.4219 | 0 |
| TCGGAT | 0.2913 | 0 |
| TCGGCA | 0.7818 | 0 |
| TCGGCC | 0.2913 | 0 |
| TCGGCG | 0.2913 | 0 |
| TCGGCT | 1 | 1 |
| TCGGGA | 0.1974 | 0 |
| TCGGGC | 0.1489 | 0 |
| TCGGGG | -0.3027 | 0 |
| TCGGGT | -0.112 | 0 |
| TCGGTA | 0 | 0 |
| TCGGTC | 0 | 0 |
| TCGGTG | 0.112 | 0 |
| TCGGTT | -0.1651 | 0 |
| TCGTAA | 0.112 | 0 |
| TCGTAC | -0.1489 | 0 |
| TCGTAG | -0.1651 | 0 |
| TCGTAT | -0.4219 | 0 |
| TCGTCA | 1 | 1 |
| TCGTCC | 0.1974 | 0 |
| TCGTCG | 1 | 1 |
| TCGTCT | 0.2913 | 0 |
| TCGTGA | 0.7818 | 0 |
| TCGTGC | 0.112 | 0 |
| TCGTGG | 0.1651 | 0 |
| TCGTGT | -0.1974 | 0 |
| TCGTTA | -0.7668 | 0 |
| TCGTTC | -1 | 1 |
| TCGTTG | -0.3027 | 0 |
| TCGTTT | -0.9468 | 0 |
| TCTAAA | -0.1651 | 0 |
| TCTAAC | 0.112 | 0 |
| TCTAAG | 0 | 0 |
| TCTAAT | -0.6351 | 0 |
| TCTACA | 0.4219 | 0 |
| TCTACC | 0 | 0 |
| TCTACG | 0.112 | 0 |
| TCTACT | -0.1974 | 0 |
| TCTAGA | 0.2449 | 0 |
| TCTAGC | -0.112 | 0 |
| TCTAGG | -1 | 1 |
| TCTAGT | -0.7818 | 0 |
| TCTATA | -0.8854 | 0 |
| TCTATC | -0.2913 | 0 |
| TCTATG | -0.7818 | 0 |
| TCTATT | -1 | 1 |
| TCTCAA | 0.112 | 0 |
| TCTCAC | 0.04996 | 0 |
| TCTCAG | 0 | 0 |
| TCTCAT | -0.4621 | 0 |
| TCTCCA | 0.4219 | 0 |
| TCTCCC | 0.04996 | 0 |
| TCTCCG | 0.04996 | 0 |
| TCTCCT | 0.3364 | 0 |
| TCTCGA | 0.3027 | 0 |
| TCTCGC | 0 | 0 |
| TCTCGG | 0.112 | 0 |
| TCTCGT | -0.1489 | 0 |
| TCTCTA | -1 | 1 |
| TCTCTC | -0.4777 | 0 |
| TCTCTG | 0.2153 | 0 |
| TCTCTT | -1 | 1 |
| TCTGAA | 1 | 1 |
| TCTGAC | 1 | 1 |
| TCTGAG | 0.9468 | 0 |
| TCTGAT | 0.3364 | 0 |
| TCTGCA | 1 | 1 |
| TCTGCC | 0.978 | 0 |
| TCTGCG | 0.6351 | 0 |
| TCTGCT | 1 | 1 |
| TCTGGA | 1 | 1 |
| TCTGGC | 0.8854 | 0 |
| TCTGGG | -0.2153 | 0 |
| TCTGGT | 0.2449 | 0 |
| TCTGTA | -0.3364 | 0 |
| TCTGTC | -0.1651 | 0 |
| TCTGTG | 0.2449 | 0 |
| TCTGTT | -1 | 1 |
| TCTTAA | -0.4355 | 0 |
| TCTTAC | -0.112 | 0 |
| TCTTAG | -0.8854 | 0 |
| TCTTAT | -1 | 1 |
| TCTTCA | 1 | 1 |
| TCTTCC | 0.112 | 0 |
| TCTTCG | 0.7818 | 0 |
| TCTTCT | -0.2153 | 0 |
| TCTTGA | 0.664 | 0 |
| TCTTGC | 0.04996 | 0 |
| TCTTGG | -0.1651 | 0 |
| TCTTGT | -0.5459 | 0 |
| TCTTTA | -1 | 1 |
| TCTTTC | -0.9468 | 0 |
| TCTTTG | -0.7818 | 0 |
| TCTTTT | -1 | 1 |
| TGAAAA | 0.9468 | 0 |
| TGAAAC | 1 | 1 |
| TGAAAG | 1 | 1 |
| TGAAAT | 0.4136 | 0 |
| TGAACA | 1 | 1 |
| TGAACC | 1 | 1 |
| TGAACG | 0.9973 | 0 |
| TGAACT | 0.9712 | 0 |
| TGAAGA | 1 | 1 |
| TGAAGC | 1 | 1 |
| TGAAGG | 1 | 1 |
| TGAAGT | 1 | 1 |
| TGAATA | -0.2449 | 0 |
| TGAATC | 0.8854 | 0 |
| TGAATG | 0.3364 | 0 |
| TGAATT | -0.112 | 0 |
| TGACAA | 0.8854 | 0 |
| TGACAC | 0.4621 | 0 |
| TGACAG | 0.6351 | 0 |
| TGACAT | 0.4219 | 0 |
| TGACCA | 1 | 1 |
| TGACCC | 0.9919 | 0 |
| TGACCG | 0.978 | 0 |
| TGACCT | 1 | 1 |
| TGACGA | 1 | 1 |
| TGACGC | 0.6351 | 0 |
| TGACGG | 0.7818 | 0 |
| TGACGT | 1 | 1 |
| TGACTA | 0.1651 | 0 |
| TGACTC | 0.2913 | 0 |
| TGACTG | 1 | 1 |
| TGACTT | 0.3027 | 0 |
| TGAGAA | 1 | 1 |
| TGAGAC | 0.978 | 0 |
| TGAGAG | 0.7668 | 0 |
| TGAGAT | 0.664 | 0 |
| TGAGCA | 1 | 1 |
| TGAGCC | 0.7818 | 0 |
| TGAGCG | 0.2913 | 0 |
| TGAGCT | 0.978 | 0 |
| TGAGGA | 1 | 1 |
| TGAGGC | 1 | 1 |
| TGAGGG | 0.3746 | 0 |
| TGAGGT | 0.6351 | 0 |
| TGAGTA | 0.3027 | 0 |
| TGAGTC | 0.1489 | 0 |
| TGAGTG | -0.1651 | 0 |
| TGAGTT | -0.2913 | 0 |
| TGATAA | -0.1651 | 0 |
| TGATAC | 0 | 0 |
| TGATAG | -0.3027 | 0 |
| TGATAT | -0.3364 | 0 |
| TGATCA | 0.9468 | 0 |
| TGATCC | 0.5459 | 0 |
| TGATCG | 0.04996 | 0 |
| TGATCT | 0.6351 | 0 |
| TGATGA | 1 | 1 |
| TGATGC | 0.537 | 0 |
| TGATGG | -0.3364 | 0 |
| TGATGT | 0.3746 | 0 |
| TGATTA | -0.664 | 0 |
| TGATTC | 0.1974 | 0 |
| TGATTG | -0.112 | 0 |
| TGATTT | -0.6351 | 0 |
| TGCAAA | 0.3364 | 0 |
| TGCAAC | 1 | 1 |
| TGCAAG | 0.8854 | 0 |
| TGCAAT | 0.112 | 0 |
| TGCACA | 0.1974 | 0 |
| TGCACC | 0.4621 | 0 |
| TGCACG | 0.1489 | 0 |
| TGCACT | 0.6351 | 0 |
| TGCAGA | 1 | 1 |
| TGCAGC | 1 | 1 |
| TGCAGG | 0.7668 | 0 |
| TGCAGT | 0.664 | 0 |
| TGCATA | -1 | 1 |
| TGCATC | 0.3364 | 0 |
| TGCATG | -0.4219 | 0 |
| TGCATT | -0.2449 | 0 |
| TGCCAA | 0.04996 | 0 |
| TGCCAC | 0.2913 | 0 |
| TGCCAG | 0.1489 | 0 |
| TGCCAT | 0.112 | 0 |
| TGCCCA | 0.2913 | 0 |
| TGCCCC | 0.1489 | 0 |
| TGCCCG | 0.2913 | 0 |
| TGCCCT | 1 | 1 |
| TGCCGA | 0.6351 | 0 |
| TGCCGC | 0.1489 | 0 |
| TGCCGG | 0.112 | 0 |
| TGCCGT | 0.3027 | 0 |
| TGCCTA | -0.04996 | 0 |
| TGCCTC | 0.1489 | 0 |
| TGCCTG | 0.5459 | 0 |
| TGCCTT | 0.1651 | 0 |
| TGCGAA | 0.7818 | 0 |
| TGCGAC | 1 | 1 |
| TGCGAG | 0.112 | 0 |
| TGCGAT | 0.1489 | 0 |
| TGCGCA | 0.2913 | 0 |
| TGCGCC | 0.04996 | 0 |
| TGCGCG | 0.04996 | 0 |
| TGCGCT | 0.2913 | 0 |
| TGCGGA | 1 | 1 |
| TGCGGC | 0.9468 | 0 |
| TGCGGG | 0.1974 | 0 |
| TGCGGT | 0.1974 | 0 |
| TGCGTA | -0.112 | 0 |
| TGCGTC | 0.04996 | 0 |
| TGCGTG | -0.04996 | 0 |
| TGCGTT | -0.2913 | 0 |
| TGCTAA | 0 | 0 |
| TGCTAC | 0.2913 | 0 |
| TGCTAG | -0.4621 | 0 |
| TGCTAT | 0 | 0 |
| TGCTCA | 0.3027 | 0 |
| TGCTCC | 0.3027 | 0 |
| TGCTCG | 0.2913 | 0 |
| TGCTCT | 1 | 1 |
| TGCTGA | 1 | 1 |
| TGCTGC | 1 | 1 |
| TGCTGG | 0.7668 | 0 |
| TGCTGT | 1 | 1 |
| TGCTTA | -0.664 | 0 |
| TGCTTC | -0.112 | 0 |
| TGCTTG | -0.1489 | 0 |
| TGCTTT | -0.4355 | 0 |
| TGGAAA | 1 | 1 |
| TGGAAC | 1 | 1 |
| TGGAAG | 1 | 1 |
| TGGAAT | 1 | 1 |
| TGGACA | 1 | 1 |
| TGGACC | 1 | 1 |
| TGGACG | 1 | 1 |
| TGGACT | 1 | 1 |
| TGGAGA | 1 | 1 |
| TGGAGC | 1 | 1 |
| TGGAGG | 1 | 1 |
| TGGAGT | 0.9081 | 0 |
| TGGATA | 0.5503 | 0 |
| TGGATC | 1 | 1 |
| TGGATG | 1 | 1 |
| TGGATT | 0.7243 | 0 |
| TGGCAA | 0.112 | 0 |
| TGGCAC | 0.112 | 0 |
| TGGCAG | 1 | 1 |
| TGGCAT | -0.2449 | 0 |
| TGGCCA | 0.4621 | 0 |
| TGGCCC | 0.4621 | 0 |
| TGGCCG | 1 | 1 |
| TGGCCT | 0.7818 | 0 |
| TGGCGA | 0.664 | 0 |
| TGGCGC | 0.4621 | 0 |
| TGGCGG | 1 | 1 |
| TGGCGT | 0.3027 | 0 |
| TGGCTA | 0.112 | 0 |
| TGGCTC | 0.4621 | 0 |
| TGGCTG | 1 | 1 |
| TGGCTT | -0.2449 | 0 |
| TGGGAA | 0.1651 | 0 |
| TGGGAC | 0.1974 | 0 |
| TGGGAG | -1 | 1 |
| TGGGAT | -0.2449 | 0 |
| TGGGCA | 0.1974 | 0 |
| TGGGCC | 0.1489 | 0 |
| TGGGCG | 0 | 0 |
| TGGGCT | 0.1974 | 0 |
| TGGGGA | -1 | 1 |
| TGGGGC | -0.3746 | 0 |
| TGGGGG | -1 | 1 |
| TGGGGT | -1 | 1 |
| TGGGTA | -0.9919 | 0 |
| TGGGTC | -0.1974 | 0 |
| TGGGTG | -1 | 1 |
| TGGGTT | -0.9992 | 0 |
| TGGTAA | -0.537 | 0 |
| TGGTAC | 0 | 0 |
| TGGTAG | -1 | 1 |
| TGGTAT | -0.8483 | 0 |
| TGGTCA | 0.3364 | 0 |
| TGGTCC | 1 | 1 |
| TGGTCG | 0.3364 | 0 |
| TGGTCT | 0.3027 | 0 |
| TGGTGA | 1 | 1 |
| TGGTGC | 0.4219 | 0 |
| TGGTGG | -1 | 1 |
| TGGTGT | -0.4621 | 0 |
| TGGTTA | -1 | 1 |
| TGGTTC | -0.537 | 0 |
| TGGTTG | -0.8617 | 0 |
| TGGTTT | -1 | 1 |
| TGTAAA | -1 | 1 |
| TGTAAC | -0.2153 | 0 |
| TGTAAG | -0.7243 | 0 |
| TGTAAT | -1 | 1 |
| TGTACA | 0 | 0 |
| TGTACC | 0 | 0 |
| TGTACG | 0 | 0 |
| TGTACT | -0.2913 | 0 |
| TGTAGA | 1 | 1 |
| TGTAGC | 0.3746 | 0 |
| TGTAGG | -1 | 1 |
| TGTAGT | -1 | 1 |
| TGTATA | -1 | 1 |
| TGTATC | -0.664 | 0 |
| TGTATG | -1 | 1 |
| TGTATT | -1 | 1 |
| TGTCAA | -0.2153 | 0 |
| TGTCAC | -0.1489 | 0 |
| TGTCAG | -0.1974 | 0 |
| TGTCAT | -1 | 1 |
| TGTCCA | 0.3027 | 0 |
| TGTCCC | 0 | 0 |
| TGTCCG | 0.1651 | 0 |
| TGTCCT | 1 | 1 |
| TGTCGA | 0.8854 | 0 |
| TGTCGC | 0.4621 | 0 |
| TGTCGG | 0.1974 | 0 |
| TGTCGT | -0.3364 | 0 |
| TGTCTA | -0.7818 | 0 |
| TGTCTC | -0.4621 | 0 |
| TGTCTG | -0.2449 | 0 |
| TGTCTT | -1 | 1 |
| TGTGAA | 0.8005 | 0 |
| TGTGAC | 0.6351 | 0 |
| TGTGAG | 0.2449 | 0 |
| TGTGAT | -0.3027 | 0 |
| TGTGCA | 1 | 1 |
| TGTGCC | 0.4219 | 0 |
| TGTGCG | 1 | 1 |
| TGTGCT | 1 | 1 |
| TGTGGA | 1 | 1 |
| TGTGGC | 1 | 1 |
| TGTGGG | -0.3799 | 0 |
| TGTGGT | 0 | 0 |
| TGTGTA | -0.7243 | 0 |
| TGTGTC | -0.3364 | 0 |
| TGTGTG | -0.6815 | 0 |
| TGTGTT | -1 | 1 |
| TGTTAA | -1 | 1 |
| TGTTAC | -0.9468 | 0 |
| TGTTAG | -1 | 1 |
| TGTTAT | -1 | 1 |
| TGTTCA | -1 | 1 |
| TGTTCC | -1 | 1 |
| TGTTCG | -0.7668 | 0 |
| TGTTCT | -0.7818 | 0 |
| TGTTGA | 0.2636 | 0 |
| TGTTGC | -0.1651 | 0 |
| TGTTGG | -0.7668 | 0 |
| TGTTGT | -0.7668 | 0 |
| TGTTTA | -1 | 1 |
| TGTTTC | -1 | 1 |
| TGTTTG | -1 | 1 |
| TGTTTT | -1 | 1 |
| TTAAAA | -1 | 1 |
| TTAAAC | -0.3364 | 0 |
| TTAAAG | -0.2913 | 0 |
| TTAAAT | -1 | 1 |
| TTAACA | -1 | 1 |
| TTAACC | -0.2153 | 0 |
| TTAACG | -0.3027 | 0 |
| TTAACT | -1 | 1 |
| TTAAGA | 0.2153 | 0 |
| TTAAGC | 0 | 0 |
| TTAAGG | -0.3746 | 0 |
| TTAAGT | -0.8483 | 0 |
| TTAATA | -1 | 1 |
| TTAATC | -0.6351 | 0 |
| TTAATG | -1 | 1 |
| TTAATT | -1 | 1 |
| TTACAA | -0.2449 | 0 |
| TTACAC | -0.04996 | 0 |
| TTACAG | 0 | 0 |
| TTACAT | -0.664 | 0 |
| TTACCA | -0.1974 | 0 |
| TTACCC | -0.04996 | 0 |
| TTACCG | -0.04996 | 0 |
| TTACCT | -0.1651 | 0 |
| TTACGA | 0.1974 | 0 |
| TTACGC | -0.1489 | 0 |
| TTACGG | -0.1489 | 0 |
| TTACGT | -0.3027 | 0 |
| TTACTA | -1 | 1 |
| TTACTC | -0.1974 | 0 |
| TTACTG | -0.5459 | 0 |
| TTACTT | -0.978 | 0 |
| TTAGAA | 0.3364 | 0 |
| TTAGAC | 0 | 0 |
| TTAGAG | -0.1974 | 0 |
| TTAGAT | -1 | 1 |
| TTAGCA | 0 | 0 |
| TTAGCC | 0 | 0 |
| TTAGCG | -0.1489 | 0 |
| TTAGCT | -0.4219 | 0 |
| TTAGGA | -0.493 | 0 |
| TTAGGC | -1 | 1 |
| TTAGGG | -1 | 1 |
| TTAGGT | -1 | 1 |
| TTAGTA | -1 | 1 |
| TTAGTC | -0.8854 | 0 |
| TTAGTG | -1 | 1 |
| TTAGTT | -1 | 1 |
| TTATAA | -1 | 1 |
| TTATAC | -0.6351 | 0 |
| TTATAG | -0.9973 | 0 |
| TTATAT | -1 | 1 |
| TTATCA | -0.3027 | 0 |
| TTATCC | -0.04996 | 0 |
| TTATCG | -0.1489 | 0 |
| TTATCT | -0.9468 | 0 |
| TTATGA | -0.7243 | 0 |
| TTATGC | -1 | 1 |
| TTATGG | -1 | 1 |
| TTATGT | -1 | 1 |
| TTATTA | -1 | 1 |
| TTATTC | -0.9919 | 0 |
| TTATTG | -1 | 1 |
| TTATTT | -1 | 1 |
| TTCAAA | 0.2153 | 0 |
| TTCAAC | 0.8483 | 0 |
| TTCAAG | 0.6351 | 0 |
| TTCAAT | -0.2449 | 0 |
| TTCACA | -0.1651 | 0 |
| TTCACC | 0.1974 | 0 |
| TTCACG | 0.112 | 0 |
| TTCACT | -0.3027 | 0 |
| TTCAGA | 1 | 1 |
| TTCAGC | 1 | 1 |
| TTCAGG | 0.4355 | 0 |
| TTCAGT | 0 | 0 |
| TTCATA | -1 | 1 |
| TTCATC | -0.2913 | 0 |
| TTCATG | -1 | 1 |
| TTCATT | -1 | 1 |
| TTCCAA | 0.2913 | 0 |
| TTCCAC | 0.2913 | 0 |
| TTCCAG | 0 | 0 |
| TTCCAT | -0.3027 | 0 |
| TTCCCA | 0 | 0 |
| TTCCCC | 0.05411 | 0 |
| TTCCCG | 0 | 0 |
| TTCCCT | -0.3027 | 0 |
| TTCCGA | 0 | 0 |
| TTCCGC | 0 | 0 |
| TTCCGG | 0.2153 | 0 |
| TTCCGT | -1 | 1 |
| TTCCTA | -0.8483 | 0 |
| TTCCTC | 1 | 1 |
| TTCCTG | -1 | 1 |
| TTCCTT | -1 | 1 |
| TTCGAA | 1 | 1 |
| TTCGAC | 0.8854 | 0 |
| TTCGAG | 0.4219 | 0 |
| TTCGAT | 0 | 0 |
| TTCGCA | 0 | 0 |
| TTCGCC | 0 | 0 |
| TTCGCG | 0 | 0 |
| TTCGCT | -0.2913 | 0 |
| TTCGGA | 0.3027 | 0 |
| TTCGGC | 0.3027 | 0 |
| TTCGGG | -0.1974 | 0 |
| TTCGGT | -0.3027 | 0 |
| TTCGTA | -1 | 1 |
| TTCGTC | -0.2153 | 0 |
| TTCGTG | -0.8854 | 0 |
| TTCGTT | -1 | 1 |
| TTCTAA | -0.664 | 0 |
| TTCTAC | -0.4621 | 0 |
| TTCTAG | -1 | 1 |
| TTCTAT | -1 | 1 |
| TTCTCA | -0.3027 | 0 |
| TTCTCC | -0.112 | 0 |
| TTCTCG | -0.1974 | 0 |
| TTCTCT | -1 | 1 |
| TTCTGA | 0.3746 | 0 |
| TTCTGC | 0.4621 | 0 |
| TTCTGG | -0.3364 | 0 |
| TTCTGT | -1 | 1 |
| TTCTTA | -1 | 1 |
| TTCTTC | -0.2913 | 0 |
| TTCTTG | -0.978 | 0 |
| TTCTTT | -1 | 1 |
| TTGAAA | 0.8617 | 0 |
| TTGAAC | 1 | 1 |
| TTGAAG | 1 | 1 |
| TTGAAT | 0.3746 | 0 |
| TTGACA | 0.2449 | 0 |
| TTGACC | 1 | 1 |
| TTGACG | 0.9468 | 0 |
| TTGACT | 0.2449 | 0 |
| TTGAGA | 0.9468 | 0 |
| TTGAGC | 0.9919 | 0 |
| TTGAGG | 1 | 1 |
| TTGAGT | 0.1651 | 0 |
| TTGATA | -0.6351 | 0 |
| TTGATC | 0.3027 | 0 |
| TTGATG | 0.2636 | 0 |
| TTGATT | -0.4621 | 0 |
| TTGCAA | 0.2913 | 0 |
| TTGCAC | 0.1489 | 0 |
| TTGCAG | 0.8854 | 0 |
| TTGCAT | -0.3027 | 0 |
| TTGCCA | 0.1489 | 0 |
| TTGCCC | 0.2913 | 0 |
| TTGCCG | 0.04996 | 0 |
| TTGCCT | -0.04996 | 0 |
| TTGCGA | 1 | 1 |
| TTGCGC | 0.04996 | 0 |
| TTGCGG | 0.4219 | 0 |
| TTGCGT | -0.1974 | 0 |
| TTGCTA | -0.3027 | 0 |
| TTGCTC | 0 | 0 |
| TTGCTG | 0.3746 | 0 |
| TTGCTT | -0.6351 | 0 |
| TTGGAA | 1 | 1 |
| TTGGAC | 1 | 1 |
| TTGGAG | 1 | 1 |
| TTGGAT | 1 | 1 |
| TTGGCA | 0.112 | 0 |
| TTGGCC | 0.4621 | 0 |
| TTGGCG | 0.3027 | 0 |
| TTGGCT | 0.3027 | 0 |
| TTGGGA | -0.3746 | 0 |
| TTGGGC | -0.4219 | 0 |
| TTGGGG | -1 | 1 |
| TTGGGT | -0.9468 | 0 |
| TTGGTA | -0.664 | 0 |
| TTGGTC | 0.1974 | 0 |
| TTGGTG | -0.2913 | 0 |
| TTGGTT | -0.8483 | 0 |
| TTGTAA | -0.8483 | 0 |
| TTGTAC | -0.2449 | 0 |
| TTGTAG | -0.6469 | 0 |
| TTGTAT | -1 | 1 |
| TTGTCA | -0.3027 | 0 |
| TTGTCC | 0.1974 | 0 |
| TTGTCG | 0.1974 | 0 |
| TTGTCT | -0.8854 | 0 |
| TTGTGA | 0 | 0 |
| TTGTGC | 0.2153 | 0 |
| TTGTGG | -0.3799 | 0 |
| TTGTGT | -1 | 1 |
| TTGTTA | -1 | 1 |
| TTGTTC | -1 | 1 |
| TTGTTG | -1 | 1 |
| TTGTTT | -1 | 1 |
| TTTAAA | -1 | 1 |
| TTTAAC | -1 | 1 |
| TTTAAG | -0.8483 | 0 |
| TTTAAT | -1 | 1 |
| TTTACA | -1 | 1 |
| TTTACC | -1 | 1 |
| TTTACG | -0.7818 | 0 |
| TTTACT | -1 | 1 |
| TTTAGA | -0.5503 | 0 |
| TTTAGC | -0.5459 | 0 |
| TTTAGG | -1 | 1 |
| TTTAGT | -1 | 1 |
| TTTATA | -1 | 1 |
| TTTATC | -0.9992 | 0 |
| TTTATG | -1 | 1 |
| TTTATT | -1 | 1 |
| TTTCAA | -0.4219 | 0 |
| TTTCAC | -0.3027 | 0 |
| TTTCAG | 0.2153 | 0 |
| TTTCAT | -1 | 1 |
| TTTCCA | -0.6351 | 0 |
| TTTCCC | -0.4942 | 0 |
| TTTCCG | -0.4621 | 0 |
| TTTCCT | -1 | 1 |
| TTTCGA | 0.2449 | 0 |
| TTTCGC | -0.1489 | 0 |
| TTTCGG | -0.4621 | 0 |
| TTTCGT | -1 | 1 |
| TTTCTA | -1 | 1 |
| TTTCTC | -1 | 1 |
| TTTCTG | -0.9992 | 0 |
| TTTCTT | -1 | 1 |
| TTTGAA | 0.6351 | 0 |
| TTTGAC | 0.3027 | 0 |
| TTTGAG | 0.2449 | 0 |
| TTTGAT | -0.5459 | 0 |
| TTTGCA | -0.2913 | 0 |
| TTTGCC | -0.1489 | 0 |
| TTTGCG | 0 | 0 |
| TTTGCT | -1 | 1 |
| TTTGGA | 1 | 1 |
| TTTGGC | -0.2449 | 0 |
| TTTGGG | -1 | 1 |
| TTTGGT | -0.9074 | 0 |
| TTTGTA | -1 | 1 |
| TTTGTC | -0.9468 | 0 |
| TTTGTG | -0.9919 | 0 |
| TTTGTT | -1 | 1 |
| TTTTAA | -1 | 1 |
| TTTTAC | -0.9973 | 0 |
| TTTTAG | -1 | 1 |
| TTTTAT | -1 | 1 |
| TTTTCA | -1 | 1 |
| TTTTCC | -0.9081 | 0 |
| TTTTCG | -0.8854 | 0 |
| TTTTCT | -1 | 1 |
| TTTTGA | -0.664 | 0 |
| TTTTGC | -0.8854 | 0 |
| TTTTGG | -1 | 1 |
| TTTTGT | -1 | 1 |
| TTTTTA | -1 | 1 |
| TTTTTC | -1 | 1 |
| TTTTTG | -1 | 1 |
| TTTTTT | -1 | 1 |
